# Zinc‐Promoted ZnMe/ZnPh Exchange in Eight‐Coordinate [Ru(PPh_3_)_2_(ZnMe)_4_H_2_]

**DOI:** 10.1002/anie.202117495

**Published:** 2022-03-14

**Authors:** Lia Sotorrios, Fedor M. Miloserdov, Anne‐Frédérique Pécharman, John P. Lowe, Stuart A. Macgregor, Mary F. Mahon, Michael K. Whittlesey

**Affiliations:** ^1^ Institute of Chemical Sciences Heriot-Watt University Edinburgh EH14 4AS UK; ^2^ Department of Chemistry University of Bath Bath BA2 3QD UK; ^3^ Present address: Laboratory of Organic Chemistry Wageningen University Stippeneng 4 Wageningen 6708 WE The Netherlands

**Keywords:** Density Functional Calculations, Heterometallic Complexes, Hydrides, Ruthenium, Zinc

## Abstract

The syntheses, reactivity and electronic structure analyses of [Ru(PPh_3_)_2_(ZnMe)_4_H_2_], **1 a**, and [Ru(PPh_3_)_2_(ZnPh)_4_H_2_], **2 b**, are reported. **1 a** exhibits an 8‐coordinate Ru centre with axial phosphines and a symmetrical (2 : 2) arrangement of ZnMe ligands in the equatorial plane. The ZnMe ligands in **1 a** undergo facile, sequential exchange with ZnPh_2_ to give **2 b**, which shows a 3 : 1 arrangement of ZnPh ligands. Both **1 a** and **2 b** exist in equilibrium with their respective 3 : 1 and 2 : 2 isomers. Mechanisms for ZnMe/ZnPh exchange and isomerisation are proposed using DFT calculations. The relationships of these {Ru(ZnR)_4_H_2_} species to isoelectronic Group 8 transition metal polyhydrides and related Schlenk equilibria in the Negishi reaction are discussed.

## Graphical abstract

Reaction of the 8‐coordinate RuZn_4_ heterobimetallic complex [Ru(PPh_3_)_2_(ZnMe)_4_H_2_] with ZnPh_2_ leads to complete ZnMe/ZnPh exchange to form [Ru(PPh_3_)_2_(ZnPh)_4_H_2_]. Both species exhibit equilibria in solution with their alternative 3 : 1 and 2 : 2 isomers and DFT calculations define facile isomerisation and hydride‐assisted ZnMe/ZnPh exchange pathways.

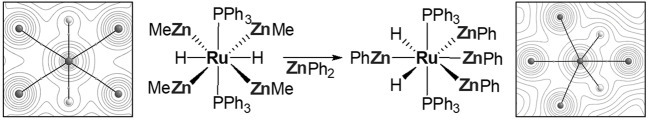

The chemistry of transition metal (TM)–main group metal (MGM) heterobimetallic complexes has undergone a renaissance in recent years due to the ability of such species to bring about the activation of element–element bonds.[^1^, ^2^] Of the many synthetic routes to TM–MGM complexes, we have focussed on the reactions of TM‐H precursors with MGM–alkyl reagents. The resultant elimination of an alkane not only provides a driving force for the process but can also result in the formation of “dual unsaturated” heterobimetallics, in which both the TM and MGM centres are coordinatively unsaturated. In such cases, both the TM and MGM are in principle available for small molecule activation. Scheme 1 shows examples of Ru‐MGM complexes that show such reactivity. The RuZn complexes **A**–**D** add H_2_ across the Ru−Zn bonds,^[3]^ while **C** also cleaved the C−H bond in PhC≡CH.^[3b]^ Intramolecular reactions are also possible: **D** forms via Zn‐promoted reductive coupling between the hydride and cyclometallated phosphine in **E**,^[3d]^ while the reaction of **F** with CO induces Me transfer across the Ru−In bond.^[4]^

**Scheme 1:**
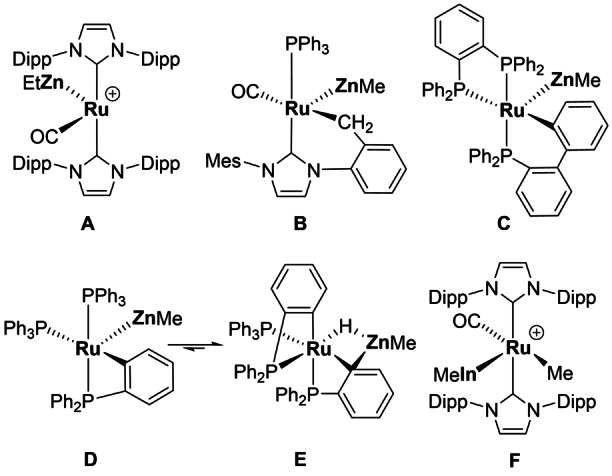
Dual unsaturated Ru–MGM complexes (Dipp=2,6‐^i^Pr_2_C_6_H_3_; Mes=2,4,6‐Me_3_C_6_H_2_): **A**,^[3a]^ **B**,^[3c]^ **C**,^[3b]^ **D**/**E** ^[3d]^ and **F**.^[4]^

In the cases of **A**, **D** and **F**, computational studies have shown that the unsaturated Ru centre is the initial site of reactivity with small molecules (e.g. H_2_, CO), with the MGM subsequently acting as a hydride or methyl acceptor. We now report the novel RuZn_4_ complex, [Ru(PPh_3_)_2_(ZnMe)_4_H_2_] (**1 a**), in which we show that the peripheral ZnR ligands can also be the centre of reactivity (Scheme 2). Thus, **1 a** is able to activate the Zn−C bond in ZnPh_2_ at room temperature to form the fully ZnMe/ZnPh‐exchanged product [Ru(PPh_3_)_2_(ZnPh)_4_H_2_] **2 b**. DFT studies show that the initial approach of ZnPh_2_ is facilitated by a hydride ligand that then enables Ph group transfer onto an adjacent ZnMe centre and thus, upon ZnMePh loss, a net ZnMe/ZnPh exchange.

**Scheme 2:**
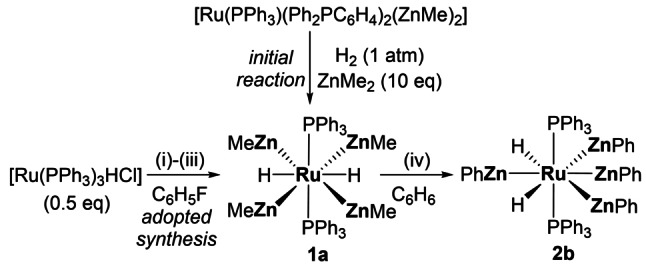
Formation of [Ru(PPh_3_)_2_(ZnMe)_4_H_2_] **1 a** and [Ru(PPh_3_)_2_(ZnPh)_4_H_2_] **2 b**. Reaction conditions: i) LiCH_2_TMS (1 equiv); ii) ZnMe_2_ (10 equiv), [Ru(PPh_3_)_3_HCl] (0.5 equiv); iii) H_2_ (1 atm); iv) ZnPh_2_ (2.5 equiv).

In contrast to the clean activation of H_2_ by complexes **A**–**D**, we recently showed that exposure of [Ru(PPh_3_)(Ph_2_PC_6_H_4_)_2_(ZnMe)_2_] to H_2_ gave an inseparable mixture of species.^[5]^ When the reaction was repeated in the presence of 10 equiv of ZnMe_2_, [Ru(PPh_3_)_2_(ZnMe)_4_H_2_] (**1 a**) was formed as the major metal‐containing product, albeit over ca. 3 weeks at room temperature. A much faster (1 day), one‐pot route involved the sequential treatment of [Ru(PPh_3_)_3_HCl] with LiCH_2_TMS, ZnMe_2_ and H_2_ (Scheme 2), to give **1 a** in 60 % yield (Supporting Information).

The product exhibited a symmetrical structure (Figure 1a) with four ZnMe groups in the equatorial plane (Ru−Zn=2.4564(3)–2.4664(2) Å) and two axial PPh_3_ groups (Ru−P=2.3209(5), 2.3210(5) Å).^[6]^ A pair of ZnMe ligands lie either side of the Ru (a 2 : 2 arrangement, vide infra) separated by two, trans disposed hydride ligands. The high symmetry afforded just five resonances in the ^1^H NMR spectrum; of most note was a triplet at *δ*=−8.55 ppm and a singlet at *δ*=−0.47 ppm (relative ratio of 2 : 12) for the two hydrides and four ZnMe groups respectively.

**Figure 1:**
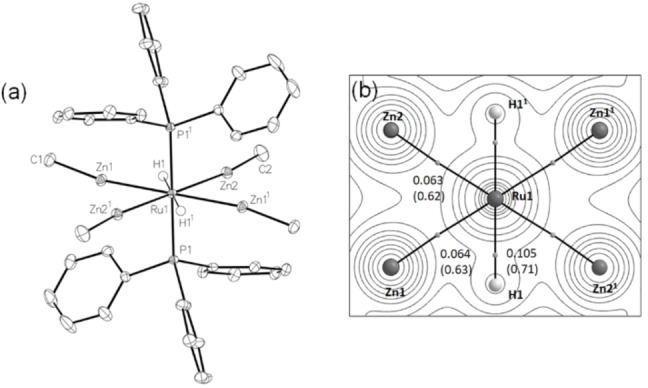
a) Molecular structure of [Ru(PPh_3_)_2_(ZnMe)_4_H_2_] (**1 a**). Thermal ellipsoids at 30 %. Labels superscripted with “1” are related to those in the asymmetric unit by the 1−*x*, 1−*y*, 1−*z* symmetry operation. b) Molecular graph for **1 a** with electron density contours plotted in the RuZn1H1 plane. Bond critical points (BCPs) are shown as grey spheres along with electron densities (*ρ*(*r*), a.u.) and delocalisation indices (in parenthesis). Out‐of‐plane phosphine ligands are omitted for clarity.

The topology of the electron density in the equatorial {RuZn_4_H_2_} plane of **1 a** taken from a QTAIM study shows the presence of Ru−Zn and Ru−H bond paths (Figure 1b). The Ru−Zn bond critical point (BCP) metrics are typical for direct Ru−Zn bonds,^[5]^ while the Ru−H BCP data are consistent with terminal Ru–hydrides. The computed Ru−H distances are 1.70 Å, as expected for a *trans*‐HRuH moiety.^[7]^ In contrast, the computed Zn⋅⋅⋅H distances (2.09 Å) are long and the Zn⋅⋅⋅Zn distances (2.71 Å) are beyond the commonly used limit denoting Zn−Zn bonding (2.68 Å).^[8]^ Accordingly, no Zn⋅⋅⋅Zn or Zn⋅⋅⋅H bond paths are seen. Evidence for some Zn⋅⋅⋅Zn and Zn⋅⋅⋅H interactions is seen in the associated delocalisation indices (DI Zn1|Zn2=0.26; Zn1|H1=0.19) and supported by ETS‐NOCV analyses and non‐covalent interaction (NCI) plots (Figure S34). However, these features are weak and the Ru centre in **1 a** is thus best described as 8‐coordinate with a hexagonal {RuZn_4_H_2_} arrangement in the equatorial plane.

We were surprised to observe no substitution of the PPh_3_ ligands in **1 a** by PCy_3_ or CO, nor insertion of CO_2_ or PhC≡CH into the Ru−H bonds. The typical reactivity of Ru phosphine hydride complexes^[9]^ thus appears to be shut down. In contrast, unexpected, facile displacement of the ZnMe ligands was observed. Treatment of **1 a** with ZnPh_2_ (2.5 equiv) led to complete exchange of ZnMe for ZnPh (<1 h, RT) to yield [Ru(PPh_3_)_2_(ZnPh)_4_H_2_] (**2 b**, Scheme 2). NMR monitoring suggested that in early stages of the reaction, mixed Ru−ZnMe/ZnPh species were formed, which upon repeated application of vacuum to remove volatile Zn species, transformed completely through to **2 b** (72 % yield).

The X‐ray crystal structure of **2 b** ^[6]^ showed a less symmetrical 3 : 1 arrangement of the ZnPh ligands (Figure 2a), with one of the ZnPh ligands located on one side of the Ru centre between the two hydrides. The Ru−Zn1 bond length (2.4342(3) Å) was shorter than the Ru−Zn2/Zn4 (2.4430(3) Å, 2.4490(3) Å) and Ru−Zn3 (2.4789(3) Å) distances.^[10]^ ZnMe exchange in **1 a** was also possible with MeLi, with the product formed, [Ru(PPh_3_)_2_(ZnMe)_3_{Li(OEt_2_)}H_2_] (**3**), also exhibiting a 3 : 1 arrangement.^[6]^

**Figure 2:**
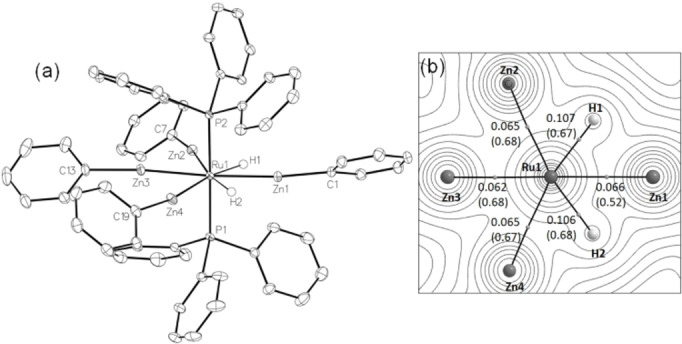
a) Molecular structure of [Ru(PPh_3_)_2_(ZnPh)_4_H_2_] (**2 b**). Thermal ellipsoids at 30 %. Solvent omitted for clarity. b) Molecular graph for **2 b** with electron density contours plotted in the RuZn1H1 plane. Bond critical points (BCPs) are shown as grey spheres along with electron densities (*ρ*(*r*), a.u.) and delocalisation indices (in parenthesis). Out‐of‐plane phosphine ligands are omitted for clarity.

QTAIM analysis of **2 b** again indicates four Ru−Zn bond paths and thus an 8‐coordinate Ru centre with a near‐planar equatorial {RuZn_4_H_2_} moiety (Figure 2b). Both the Ru−Zn and Ru−H bond paths show similar BCP *ρ*(*r*) values to those in **1 a** and the computed Ru−H distances are again 1.70 Å. We have previously found delocalisation indices to be more discriminating for Ru−Zn bonding^[5]^ and the significantly lower value computed for Ru−Zn1 suggests this Ru−Zn interaction is weakest.^[11]^ QTAIM, ETS‐NOCV and NCI analyses again indicate that any Zn⋅⋅⋅Zn and Zn⋅⋅⋅H interactions in **2 b** are weak (Supporting Information). The latter are most evident with Zn1 where the computed Zn1⋅⋅⋅H distances average 1.97 Å. The geometries of {L_*n*_Ru(H)_2_ZnR} moieties are sensitive to the coordination environment at Ru with the hydride ligands readily moving between terminal and bridging character.[^3a^, ^5^] In this case terminal hydride character dominates, consistent with an 8‐coordinate Ru centre in **2 b**.

Given the different arrangements in **1 a** and **2 b**, both compounds were examined by VT NMR spectroscopy and shown to be in equilibrium with the corresponding isomers **1 b** and **2 a** (Supporting Information). At ca. 190 K (toluene), the hydride signal of **1 a** (*δ*=−8.31 ppm) was present in a 3.2 : 1 ratio (2.7 : 1 ratio in THF) with a second hydride resonance (*δ*=−8.38 ppm) assigned to isomer **1 b**, which also showed three new ZnMe signals at *δ*=−0.24 ppm, −0.30 ppm and −0.44 ppm (relative ratio 2 : 1 : 1), consistent with a 3 : 1 arrangement of ZnMe ligands. At the same low temperature, complex **2 b** was present in a 3.2 : 1 ratio with isomer **2 a** (THF).

DFT calculations^[12]^ were performed to model the **1 a→1 b** and **2 b→2 a** isomerisations as well as the facile ZnMe/ZnPh exchange observed with **1 a**. Isomerisation proceeds in a single step in which one ZnR group moves out of the equatorial plane to allow an adjacent hydride ligand to move over the Ru−Zn connectivity, with shortened Zn⋅⋅⋅H distances of 1.84 Å computed in the transition states (Scheme 3). The calculated free energy barriers are 14.7 kcal mol^−1^ for **1** (relative to **1 a**) and 16.9 kcal mol^−1^ for **2** (relative to **2 b**), higher than those determined experimentally, but still consistent with a facile process at 298 K (Table 1).^[13]^

**Scheme 3:**
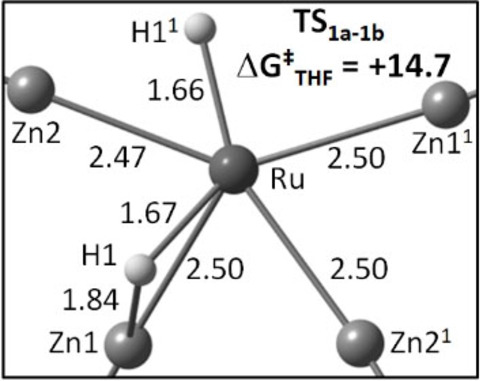
The computed isomerisation transition state, **TS_1a_**_–**1b**_, with key distances in Å; only the RuZn_4_H_2_ core is shown with axial PPh_3_ ligands removed for clarity.

**Table 1:** Experimental and computed activation barriers for isomerisation of **1 a** to **1 b** and **2 b** to **2 a**.

	Δ*H* ^≠^/Δ*G* ^≠^ [298 K, kcal mol^−1^]	
	Experiment	Computed
**1 a→1 b** (THF) **1 a→1 b** (toluene)	10.9±0.1/9.3±0.6 13.5±0.5/9.6±0.8	13.6/14.7
**2 b→2 a** (THF)	12.8±0.4/9.7±0.8	16.2/16.9

Figure 3 shows the computed profile for the first ZnMe/ZnPh substitution in **1 a**. The initial approach of ZnPh_2_ is aided by one of the hydride ligands via **TS1_Me/Ph_** (+7.4 kcal mol^−1^; Zn^2^⋅⋅⋅H^1^=1.99 Å) and leads to **Int1_Me/Ph_** at +5.5 kcal mol^−1^. Computed QTAIM charges indicate nucleophilic hydride ligands in **1 a** (*q*_H_=−0.29). In **Int1_Me/Ph_** the Ru⋅⋅⋅Zn^2^ distance shortens to 2.87 Å while the Ru−Zn^1^ distance elongates by ca. 0.3 Å to 2.77 Å. Incipient Ph group transfer to Zn^1^ is also evident (Zn^2^⋅⋅⋅Ph=2.11 Å; Zn^1^⋅⋅⋅Ph=2.26 Å) and this is completed via **TS2_Me/Ph_** at +11.5 kcal mol^−1^ along with concomitant shortening of the Ru−Zn^2^ distance (2.56 Å) and expulsion of ZnMePh (Ru⋅⋅⋅Zn^1^=3.68 Å). Throughout this process the remaining Ru−H (ca. 1.70 Å) and Ru−Zn distances (ca. 2.50 Å) are largely unaffected. This first ZnMe/ZnPh exchange proceeds with a low overall barrier of 11.5 kcal mol^−1^ and is exergonic, forming the mixed [Ru(PPh_3_)_2_(ZnMe)_3_(ZnPh)H_2_] species, **Int2_Me/Ph_**, at −2.6 kcal mol^−1^. An alternative pathway in which ZnPh_2_ approaches between two ZnMe ligands was assessed and involved a larger barrier of 17.6 kcal mol^−1^ due to a lack of stabilisation of ZnPh_2_ by a hydride ligand. The subsequent Ph group transfer to form ZnMePh does feature interaction with a hydride and so has a lower transition state at 8.2 kcal mol^−1^ (Figure S25). The three subsequent ZnMe/ZnPh exchange processes required for formation of **2 a** were exergonic by 5.7 kcal mol^−1^, 2.5 kcal mol^−1^ and 2.4 kcal mol^−1^ respectively. The full profile for the final ZnMe/ZnPh exchange in [Ru(PPh_3_)_2_(ZnMe)(ZnPh)_3_H_2_] was computed and gave an overall barrier of 15.4 kcal mol^−1^. This final step involves ZnMe_2_ loss and was modelled with MeZnPh as the Ph source to reflect the 2.5 excess of ZnPh_2_ used experimentally (Figure S27). These ZnMe/ZnPh exchange processes are therefore not significantly affected by the nature of the ZnR groups present. The 4‐fold ZnMe/ZnPh exchange upon reaction of **1 a** with ZnPh_2_ to form **2 a** is therefore both thermodynamically favourable and kinetically accessible, and, along with the final isomerisation of **2 a** (Δ*G* ^≠^=14.5 kcal mol^−1^ in toluene), should proceed readily to form **2 b** as the experimentally observed product.

**Figure 3:**
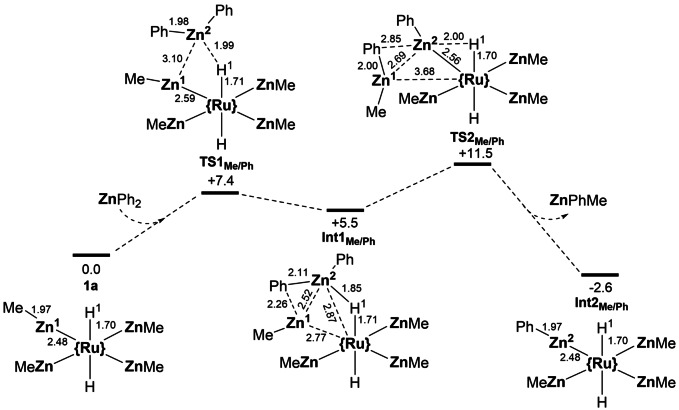
Computed free energy profile (ωB97X‐D (toluene)/def2TZVP//BP86/SDD(Ru, Zn, P, with polarisation on P), 6‐31G**, kcal mol^−1^) for the reaction of **1 a** with ZnPh_2_ to give the single ZnMe/ZnPh exchanged intermediate **Int2_Me/Ph_**. {Ru}={*trans*‐Ru(PPh_3_)_2_} with PPh_3_ ligands omitted throughout for clarity. Selected distances in Å.

In summary, we have prepared two novel 8‐coordinate RuZn_4_ complexes, the 2 : 2 complex [Ru(PPh_3_)_2_(ZnMe)_4_H_2_] (**1 a**) and the 3 : 1 complex [Ru(PPh_3_)_2_(ZnPh)_4_H_2_] (**2 b**). **1 a** and **2 b** exist in equilibrium at low temperature with the alternative 3 : 1 (**1 b**) and 2 : 2 (**2 a**) forms respectively. The reaction of **1 a** with ZnPh_2_ leads to exchange of all four ZnMe ligands to form **2 b**. Computational studies define a series of low energy ZnMe/ZnPh exchange processes in which the initial approach of the Lewis acidic ZnPh_2_ is facilitated by an electron‐rich hydride ligand.

These RuZn_4_ complexes add to the range of TM–MGM heterobimetallic complexes with hydride ligands that have been shown to access unusual coordination numbers/geometries; in particular, the hexagonal planar {RuZn_4_H_2_} moieties in this study resemble the proposed hexagonal planar {PdMg_3_H_3_} coordination geometry of the Pd centre in [PdH_3_{Mg(nacnac)}_3_].^[14]^ The isolobality of {ZnR} with a H atom has also been noted.^[8]^ In this context, the Ru−ZnR and Ru−H bonds in **1 a** and **2 b** suggest a “hexahydride” geometry, in contrast to [Ru(PR_3_)_2_H_6_] species that exist as [Ru(PR_3_)_2_(η^2^‐H_2_)_2_H_2_] (R=Cy, Cyp, ^i^Pr).^[15]^ The one Os congener, [Os(P^i^Pr_2_Ph)_2_H_6_],^[16]^ is a hexahydride, but with a very different distorted dodecahedral arrangement of the 8 ligands around the central metal. σ‐Zincane complexes have been reported[^17^, ^18^] in which the Zn−H distances vary from 1.5–1.8 Å. The only stationary points located in our [Ru(PPh_3_)_2_(ZnR)_4_H_2_] system that approached these structures were the **1 a**↔**1 b** and **2 a**↔**2 b** isomerisation transition states, with Zn⋅⋅⋅H distances of 1.84 Å, but these were computed to be 13–17 kcal mol^−1^ above the all‐terminal isomers. Also relevant is [Ru(PCy_3_)_2_(ZnMe)_2_(μ_2_‐H)_4_] reported by Fischer,^[10]^ in which the bridging hydrides have average Zn–H distances of 1.78 Å.

The clean ZnMe/ZnPh exchange in **1 a** also provides a rare, well‐defined example where the reactivity of the heterobimetallic species is centred on the MGM. Such reactivity could be of broader relevance, for example, to the understanding of the complexities of the Negishi reaction, where Pd–Zn heterobimetallic intermediates are proposed to form in the presence of an excess of a ZnX_2_ reagent.^[19]^ The involvement of alkyl/aryl coupling partners in the Negishi reaction also renders the ZnX_2_ species subject to Schlenk equilibria^[20]^ and the ZnMe/ZnPh exchange reaction reported here can be viewed in this light, where the ZnPh_2_ reagent is redistributed between {ZnMe} and {L_*n*_Ru} moieties.

## Conflict of interest

The authors declare no conflict of interest.

## Supporting information

As a service to our authors and readers, this journal provides supporting information supplied by the authors. Such materials are peer reviewed and may be re‐organized for online delivery, but are not copy‐edited or typeset. Technical support issues arising from supporting information (other than missing files) should be addressed to the authors.

Supporting Information

Supporting Information

Supporting Information

## Data Availability

The data that support the findings of this study are available in the supplementary material of this article.
