# Supplementary material for: Zinc‐Promoted ZnMe/ZnPh Exchange in Eight‐Coordinate [Ru(PPh3)2(ZnMe)4H2]
Source: Angew Chem Int Ed Engl. 2022 Mar 14;61(19):e202117495. doi: 10.1002/anie.202117495 (PMC9311408; doi:10.1002/anie.202117495)

checkCIF/PLATON report

You have not supplied any structure factors. As a result the full set of tests cannot be run.

THIS REPORT IS FOR GUIDANCE ONLY. IF USED AS PART OF A REVIEW PROCEDURE FOR PUBLICATION, IT SHOULD NOT REPLACE THE EXPERTISE OF AN EXPERIENCED CRYSTALLOGRAPHIC REFEREE.

No syntax errors found. CIF dictionary Interpreting this report

Datablock: e19mkw48

```
Bond precision:   C-C = 0.0030 A          Wavelength=0.71073

Cell:           a=9.3754(4)         b=10.1136(5)       c=11.4001(5)
                alpha=106.799(4)    beta=109.907(4)    gamma=94.877(4)
Temperature:    150 K
```

	Calculated	Reported
Volume	952.57(9)	952.57(8)
Space group	P -1	P -1
Hall group	-P 1	-P 1
Moiety formula	C40 H44 P2 Ru Zn4	C40 H44 P2 Ru Zn4
Sum formula	C40 H44 P2 Ru Zn4	C40 H44 P2 Ru Zn4
Mr	949.32	949.24
Dx,g cm-3	1.655	1.655
Z	1	1
Mu (mm-1)	2.980	2.980
F000	478.0	478.0
F000'	478.16	
h,k,lmax	13,14,16	12,14,16
Nref	5690	4787
Tmin,Tmax	0.463,0.564	0.712,1.000
Tmin'	0.123	

```
Correction method= # Reported T Limits: Tmin=0.712 Tmax=1.000
AbsCorr = MULTI-SCAN

Data completeness= 0.841           Theta(max)= 30.247

R(reflections)= 0.0215( 4357)                    wR2(reflections)=
                                                 0.0512( 4787)
S = 1.044                  Npar= 220
```

The following ALERTS were generated. Each ALERT has the format
test-name_ALERT_alert-type_alert-level.
Click on the hyperlinks for more details of the test.

Alert level G

```
PLAT012_ALERT_1_G No       _shelx_res_checksum Found in CIF ......     Please Check
PLAT063_ALERT_4_G Crystal Size Possibly too Large for Beam Size ..       0.70 mm
PLAT154_ALERT_1_G The s.u.'s on the Cell Angles are Equal ..(Note)      0.004 Degree
PLAT232_ALERT_2_G Hirshfeld Test Diff (M-X)  Ru1      --P1       .        6.0 s.u.
PLAT303_ALERT_2_G Full Occupancy Atom H1        with # Connections       3.00 Check
PLAT941_ALERT_3_G Average HKL Measurement Multiplicity ...........        1.8 Low
```

0 **ALERT level A** = Most likely a serious problem - resolve or explain
0 **ALERT level B** = A potentially serious problem, consider carefully
0 **ALERT level C** = Check. Ensure it is not caused by an omission or oversight
6 **ALERT level G** = General information/check it is not something unexpected

2 ALERT type 1 CIF construction/syntax error, inconsistent or missing data
2 ALERT type 2 Indicator that the structure model may be wrong or deficient
1 ALERT type 3 Indicator that the structure quality may be low
1 ALERT type 4 Improvement, methodology, query or suggestion
0 ALERT type 5 Informative message, check

Datablock: s19mkw50

Bond precision: C-C = 0.0043 A Wavelength=1.54184

Cell:	a=12.7497(3)	b=15.0545(3)	c=15.7170(3)
	alpha=94.169(2)	beta=95.178(2)	gamma=110.978(2)
Temperature:	150 K		

	Calculated	Reported
Volume	2787.33(11)	2787.33(11)
Space group	P -1	P -1
Hall group	-P 1	-P 1
Moiety formula	C60 H52 P2 Ru Zn4, C6 H6	C60 H52 P2 Ru Zn4, C6 H6
Sum formula	C66 H58 P2 Ru Zn4	C66 H58 P2 Ru Zn4
Mr	1275.70	1275.61
Dx,g cm-3	1.520	1.520
Z	2	2
Mu (mm-1)	4.907	4.907
F000	1296.0	1296.0
F000'	1287.14	
h,k,lmax	15,18,19	15,18,19
Nref	11237	11147
Tmin,Tmax	0.562,0.656	0.911,1.000
Tmin'	0.445	

Correction method= # Reported T Limits: Tmin=0.911 Tmax=1.000
AbsCorr = MULTI-SCAN

Data completeness= 0.992 Theta(max)= 73.535

R(reflections)= 0.0319(10920) wR2(reflections)= 0.0858(11147)

S = 1.075 Npar= 666

The following ALERTS were generated. Each ALERT has the format
test-name_ALERT_alert-type_alert-level.
Click on the hyperlinks for more details of the test.

Alert level C

PLAT243_ALERT_4_C High 'Solvent' Ueq as Compared to Neighbors of C64 Check

---

Alert level G

PLAT012_ALERT_1_G No _shelx_res_checksum Found in CIF ...... Please Check
PLAT154_ALERT_1_G The s.u.'s on the Cell Angles are Equal ..(Note) 0.002 Degree
PLAT303_ALERT_2_G Full Occupancy Atom H1 with # Connections 3.00 Check
PLAT303_ALERT_2_G Full Occupancy Atom H2 with # Connections 3.00 Check
PLAT720_ALERT_4_G Number of Unusual/Non-Standard Labels .... 1 Note
PLAT790_ALERT_4_G Centre of Gravity not Within Unit Cell: Resd. # 2 Note
C6 H6
PLAT933_ALERT_2_G Number of OMIT Records in Embedded .res File ... 2 Note
PLAT941_ALERT_3_G Average HKL Measurement Multiplicity .... 3.2 Low

---

0 **ALERT level A** = Most likely a serious problem - resolve or explain
0 **ALERT level B** = A potentially serious problem, consider carefully

```
   1 ALERT level C = Check. Ensure it is not caused by an omission or oversight
   8 ALERT level G = General information/check it is not something unexpected

   2 ALERT type 1 CIF construction/syntax error, inconsistent or missing data
   3 ALERT type 2 Indicator that the structure model may be wrong or deficient
   1 ALERT type 3 Indicator that the structure quality may be low
   3 ALERT type 4 Improvement, methodology, query or suggestion
   0 ALERT type 5 Informative message, check
```

Datablock: s19mkw34

```
Bond precision:    C-C = 0.0034 A            Wavelength=0.71073

Cell:              a=11.5032(3)     b=16.8019(3)         c=11.1990(3)
                   alpha=90         beta=116.007(3)      gamma=90
Temperature:       150 K

                 Calculated                    Reported
Volume           1945.32(9)                    1945.32(9)
Space group      P 21/c                        P 1 21/c 1
Hall group       -P 2ybc                       -P 2ybc
Moiety formula   C40 H44 P2 Ru Zn4             C40 H44 P2 Ru Zn4
Sum formula      C40 H44 P2 Ru Zn4             C40 H44 P2 Ru Zn4
Mr               949.32                        949.24
Dx,g cm-3        1.621                         1.621
Z                2                             2
Mu (mm-1)        2.919                         2.919
F000             956.0                         956.0
F000'            956.33
h,k,lmax         15,23,15                      15,22,14
Nref             5342                          4613
Tmin,Tmax        0.491,0.649                   0.892,1.000
Tmin'            0.462

Correction method= # Reported T Limits: Tmin=0.892 Tmax=1.000
AbsCorr = MULTI-SCAN

Data completeness= 0.864            Theta(max)= 29.335

                                                         wR2(reflections)=
R(reflections)= 0.0241( 4190)                            0.0597( 4613)

S = 1.051                  Npar= 220
```

The following ALERTS were generated. Each ALERT has the format
test-name_ALERT_alert-type_alert-level.
Click on the hyperlinks for more details of the test.

Alert level C

```
PLAT220_ALERT_2_C NonSolvent   Resd 1  C   Ueq(max)/Ueq(min) Range        3.6 Ratio
PLAT241_ALERT_2_C High   'MainMol' Ueq as Compared to Neighbors of        C17 Check
```

Alert level G

```
PLAT012_ALERT_1_G No       _shelx_res_checksum Found in CIF ......     Please Check
PLAT153_ALERT_1_G The s.u.'s on the Cell Axes   are Equal ..(Note)     0.0003 Ang.
PLAT303_ALERT_2_G Full Occupancy Atom H1        with # Connections       3.00 Check
PLAT933_ALERT_2_G Number of OMIT Records in Embedded .res File ...          1 Note
PLAT941_ALERT_3_G Average HKL Measurement Multiplicity ..........         3.4 Low
```

0 **ALERT level A** = Most likely a serious problem - resolve or explain
0 **ALERT level B** = A potentially serious problem, consider carefully
2 **ALERT level C** = Check. Ensure it is not caused by an omission or oversight
5 **ALERT level G** = General information/check it is not something unexpected

2 ALERT type 1 CIF construction/syntax error, inconsistent or missing data
4 ALERT type 2 Indicator that the structure model may be wrong or deficient
1 ALERT type 3 Indicator that the structure quality may be low
0 ALERT type 4 Improvement, methodology, query or suggestion
0 ALERT type 5 Informative message, check

Datablock: e19mkw50

Bond precision:	C-C = 0.0026 A		Wavelength=0.71073
Cell:	a=12.8703(2)	b=18.7512(2)	c=18.4990(2)
	alpha=90	beta=108.503(1)	gamma=90
Temperature:	150 K		

	Calculated	Reported
Volume	4233.65(10)	4233.65(9)
Space group	P 21/c	P 1 21/c 1
Hall group	-P 2ybc	-P 2ybc
Moiety formula	C43 H51 Li O P2 Ru Zn3	C43 H51 Li O P2 Ru Zn3
Sum formula	C43 H51 Li O P2 Ru Zn3	C43 H51 Li O P2 Ru Zn3
Mr	949.96	949.90
Dx,g cm-3	1.490	1.490
Z	4	4
Mu (mm-1)	2.135	2.135
F000	1936.0	1936.0
F000’	1935.57	
h,k,lmax	18,26,26	18,26,25
Nref	12522	11178
Tmin,Tmax	0.459,0.756	0.733,1.000
Tmin’	0.450	

Correction method= # Reported T Limits: Tmin=0.733 Tmax=1.000
AbsCorr = MULTI-SCAN

Data completeness= 0.893 Theta(max)= 30.156

R(reflections)= 0.0223(10018) wR2(reflections)= 0.0515(11178)

S = 1.029 Npar= 473

The following ALERTS were generated. Each ALERT has the format
test-name_ALERT_alert-type_alert-level.
Click on the hyperlinks for more details of the test.

Alert level C

PLAT220_ALERT_2_C NonSolvent Resd 1 C Ueq(max)/Ueq(min) Range 3.6 Ratio

---

Alert level G

PLAT012_ALERT_1_G No _shelx_res_checksum Found in CIF ...... Please Check
PLAT232_ALERT_2_G Hirshfeld Test Diff (M-X) Ru1 --P1 . 8.3 s.u.
PLAT232_ALERT_2_G Hirshfeld Test Diff (M-X) Ru1 --P2 . 5.7 s.u.
PLAT303_ALERT_2_G Full Occupancy Atom H1 with # Connections 2.00 Check
PLAT303_ALERT_2_G Full Occupancy Atom H2 with # Connections 2.00 Check
PLAT941_ALERT_3_G Average HKL Measurement Multiplicity .... 4.2 Low

---

0 **ALERT level A** = Most likely a serious problem - resolve or explain
0 **ALERT level B** = A potentially serious problem, consider carefully
1 **ALERT level C** = Check. Ensure it is not caused by an omission or oversight
6 **ALERT level G** = General information/check it is not something unexpected

```
1 ALERT type 1 CIF construction/syntax error, inconsistent or missing data
5 ALERT type 2 Indicator that the structure model may be wrong or deficient
1 ALERT type 3 Indicator that the structure quality may be low
0 ALERT type 4 Improvement, methodology, query or suggestion
0 ALERT type 5 Informative message, check
```

Datablock: s20mkw12

Bond precision: C-C = 0.0070 A   Wavelength=1.54184

Cell:	a=16.0723(2)	b=13.1885(1)	c=21.5154(2)
	alpha=90	beta=90.501(1)	gamma=90
Temperature:	240 K		

	Calculated	Reported
Volume	4560.44(8)	4560.44(8)
Space group	C 2/c	C 1 2/c 1
Hall group	-C 2yc	-C 2yc
Moiety formula	C39 H39 P2 Ru Zn3, C8 H16 Li O2	C47 H55 Li O2 P2 Ru Zn3
Sum formula	C47 H55 Li O2 P2 Ru Zn3	C47 H55 Li O2 P2 Ru Zn3
Mr	1018.03	1017.97
Dx,g cm-3	1.483	1.483
Z	4	4
Mu (mm-1)	5.351	5.351
F000	2080.0	2080.0
F000'	2067.60	
h,k,lmax	19,16,26	19,16,26
Nref	4566	4546
Tmin,Tmax	0.298,0.305	0.500,1.000
Tmin'	0.191	

Correction method= # Reported T Limits: Tmin=0.500 Tmax=1.000
AbsCorr = MULTI-SCAN

Data completeness= 0.996   Theta(max)= 73.185

R(reflections)= 0.0442(4376)   wR2(reflections)= 0.1115(4546)

S = 1.033   Npar= 380

The following ALERTS were generated. Each ALERT has the format
test-name_ALERT_alert-type_alert-level.
Click on the hyperlinks for more details of the test.

Alert level C

```
CRYSC01_ALERT_1_C The word below has not been recognised as a standard
            identifier.
            yellowish
PLAT220_ALERT_2_C NonSolvent   Resd 1  C   Ueq(max)/Ueq(min) Range        3.1 Ratio
PLAT242_ALERT_2_C Low    'MainMol' Ueq as Compared to Neighbors of        Ru1 Check
PLAT250_ALERT_2_C Large U3/U1 Ratio for Average U(i,j) Tensor ....        2.4 Note
PLAT260_ALERT_2_C Large Average Ueq of Residue Including        O1      0.116 Check
PLAT332_ALERT_2_C Large Phenyl C-C Range     C9        -C14      .       0.16 Ang.
PLAT332_ALERT_2_C Large Phenyl C-C Range     C9        -C14A     .       0.16 Ang.
```

Alert level G

```
PLAT002_ALERT_2_G Number of Distance or Angle Restraints on AtSite         32 Note
PLAT003_ALERT_2_G Number of Uiso or Uij Restrained non-H Atoms ...         10 Report
PLAT042_ALERT_1_G Calc. and Reported Moiety Formula Strings Differ     Please Check
PLAT083_ALERT_2_G SHELXL Second Parameter in WGHT  Unusually Large      18.78 Why ?
PLAT142_ALERT_4_G s.u. on b - Axis Small or Missing .............    0.00010 Ang.
PLAT143_ALERT_4_G s.u. on c - Axis Small or Missing .............    0.00020 Ang.
PLAT176_ALERT_4_G The CIF-Embedded .res File Contains SADI Records         32 Report
PLAT178_ALERT_4_G The CIF-Embedded .res File Contains SIMU Records          3 Report
PLAT300_ALERT_4_G Atom Site Occupancy of C10       Constrained at       0.65 Check
PLAT300_ALERT_4_G Atom Site Occupancy of C11       Constrained at       0.65 Check
PLAT300_ALERT_4_G Atom Site Occupancy of C12       Constrained at       0.65 Check
PLAT300_ALERT_4_G Atom Site Occupancy of C13       Constrained at       0.65 Check
PLAT300_ALERT_4_G Atom Site Occupancy of C14       Constrained at       0.65 Check
PLAT300_ALERT_4_G Atom Site Occupancy of C16       Constrained at       0.65 Check
PLAT300_ALERT_4_G Atom Site Occupancy of C17       Constrained at       0.65 Check
PLAT300_ALERT_4_G Atom Site Occupancy of C18       Constrained at       0.65 Check
PLAT300_ALERT_4_G Atom Site Occupancy of C19       Constrained at       0.65 Check
PLAT300_ALERT_4_G Atom Site Occupancy of C20       Constrained at       0.65 Check
PLAT300_ALERT_4_G Atom Site Occupancy of C10A      Constrained at       0.35 Check
PLAT300_ALERT_4_G Atom Site Occupancy of C11A      Constrained at       0.35 Check
PLAT300_ALERT_4_G Atom Site Occupancy of C12A      Constrained at       0.35 Check
PLAT300_ALERT_4_G Atom Site Occupancy of C13A      Constrained at       0.35 Check
PLAT300_ALERT_4_G Atom Site Occupancy of C14A      Constrained at       0.35 Check
PLAT300_ALERT_4_G Atom Site Occupancy of C16A      Constrained at       0.35 Check
PLAT300_ALERT_4_G Atom Site Occupancy of C17A      Constrained at       0.35 Check
PLAT300_ALERT_4_G Atom Site Occupancy of C18A      Constrained at       0.35 Check
PLAT300_ALERT_4_G Atom Site Occupancy of C19A      Constrained at       0.35 Check
PLAT300_ALERT_4_G Atom Site Occupancy of C20A      Constrained at       0.35 Check
PLAT300_ALERT_4_G Atom Site Occupancy of H1A       Constrained at        0.5 Check
PLAT300_ALERT_4_G Atom Site Occupancy of H1B       Constrained at        0.5 Check
PLAT300_ALERT_4_G Atom Site Occupancy of H1C       Constrained at        0.5 Check
PLAT300_ALERT_4_G Atom Site Occupancy of H10       Constrained at       0.65 Check
PLAT300_ALERT_4_G Atom Site Occupancy of H11       Constrained at       0.65 Check
PLAT300_ALERT_4_G Atom Site Occupancy of H12       Constrained at       0.65 Check
PLAT300_ALERT_4_G Atom Site Occupancy of H13       Constrained at       0.65 Check
PLAT300_ALERT_4_G Atom Site Occupancy of H14       Constrained at       0.65 Check
PLAT300_ALERT_4_G Atom Site Occupancy of H16       Constrained at       0.65 Check
PLAT300_ALERT_4_G Atom Site Occupancy of H17       Constrained at       0.65 Check
PLAT300_ALERT_4_G Atom Site Occupancy of H18       Constrained at       0.65 Check
```

```
PLAT300_ALERT_4_G Atom Site Occupancy of H19        Constrained at       0.65 Check
PLAT300_ALERT_4_G Atom Site Occupancy of H20        Constrained at       0.65 Check
PLAT300_ALERT_4_G Atom Site Occupancy of H10A       Constrained at       0.35 Check
PLAT300_ALERT_4_G Atom Site Occupancy of H11A       Constrained at       0.35 Check
PLAT300_ALERT_4_G Atom Site Occupancy of H12A       Constrained at       0.35 Check
PLAT300_ALERT_4_G Atom Site Occupancy of H13A       Constrained at       0.35 Check
PLAT300_ALERT_4_G Atom Site Occupancy of H14A       Constrained at       0.35 Check
PLAT300_ALERT_4_G Atom Site Occupancy of H16A       Constrained at       0.35 Check
PLAT300_ALERT_4_G Atom Site Occupancy of H17A       Constrained at       0.35 Check
PLAT300_ALERT_4_G Atom Site Occupancy of H18A       Constrained at       0.35 Check
PLAT300_ALERT_4_G Atom Site Occupancy of H19A       Constrained at       0.35 Check
PLAT300_ALERT_4_G Atom Site Occupancy of H20A       Constrained at       0.35 Check
PLAT300_ALERT_4_G Atom Site Occupancy of O1         Constrained at        0.5 Check
PLAT300_ALERT_4_G Atom Site Occupancy of O1A        Constrained at        0.5 Check
PLAT300_ALERT_4_G Atom Site Occupancy of C21        Constrained at        0.5 Check
PLAT300_ALERT_4_G Atom Site Occupancy of C21A       Constrained at        0.5 Check
PLAT300_ALERT_4_G Atom Site Occupancy of C22        Constrained at        0.5 Check
PLAT300_ALERT_4_G Atom Site Occupancy of C22A       Constrained at        0.5 Check
PLAT300_ALERT_4_G Atom Site Occupancy of C23        Constrained at        0.5 Check
PLAT300_ALERT_4_G Atom Site Occupancy of C23A       Constrained at        0.5 Check
PLAT300_ALERT_4_G Atom Site Occupancy of C24        Constrained at        0.5 Check
PLAT300_ALERT_4_G Atom Site Occupancy of C24A       Constrained at        0.5 Check
PLAT300_ALERT_4_G Atom Site Occupancy of H21A       Constrained at        0.5 Check
PLAT300_ALERT_4_G Atom Site Occupancy of H21B       Constrained at        0.5 Check
PLAT300_ALERT_4_G Atom Site Occupancy of H21C       Constrained at        0.5 Check
PLAT300_ALERT_4_G Atom Site Occupancy of H21D       Constrained at        0.5 Check
PLAT300_ALERT_4_G Atom Site Occupancy of H22A       Constrained at        0.5 Check
PLAT300_ALERT_4_G Atom Site Occupancy of H22B       Constrained at        0.5 Check
PLAT300_ALERT_4_G Atom Site Occupancy of H22C       Constrained at        0.5 Check
PLAT300_ALERT_4_G Atom Site Occupancy of H22D       Constrained at        0.5 Check
PLAT300_ALERT_4_G Atom Site Occupancy of H23A       Constrained at        0.5 Check
PLAT300_ALERT_4_G Atom Site Occupancy of H23B       Constrained at        0.5 Check
PLAT300_ALERT_4_G Atom Site Occupancy of H23C       Constrained at        0.5 Check
PLAT300_ALERT_4_G Atom Site Occupancy of H23D       Constrained at        0.5 Check
PLAT300_ALERT_4_G Atom Site Occupancy of H24A       Constrained at        0.5 Check
PLAT300_ALERT_4_G Atom Site Occupancy of H24B       Constrained at        0.5 Check
PLAT300_ALERT_4_G Atom Site Occupancy of H24C       Constrained at        0.5 Check
PLAT300_ALERT_4_G Atom Site Occupancy of H24D       Constrained at        0.5 Check
PLAT301_ALERT_3_G Main Residue  Disorder .............(Resd  1  )        44% Note
PLAT302_ALERT_4_G Anion/Solvent/Minor-Residue Disorder (Resd  2  )        91% Note
PLAT789_ALERT_4_G Atoms with Negative _atom_site_disorder_group  #          3 Check
PLAT790_ALERT_4_G Centre of Gravity not Within Unit Cell: Resd.  #          2 Note
              C8 H16 Li O2
PLAT811_ALERT_5_G No ADDSYM Analysis: Too Many Excluded Atoms ....          ! Info
PLAT860_ALERT_3_G Number of Least-Squares Restraints ............          74 Note
```

---

0 **ALERT level A** = Most likely a serious problem - resolve or explain
0 **ALERT level B** = A potentially serious problem, consider carefully
7 **ALERT level C** = Check. Ensure it is not caused by an omission or oversight
83 **ALERT level G** = General information/check it is not something unexpected

2 ALERT type 1 CIF construction/syntax error, inconsistent or missing data
9 ALERT type 2 Indicator that the structure model may be wrong or deficient
2 ALERT type 3 Indicator that the structure quality may be low
76 ALERT type 4 Improvement, methodology, query or suggestion
1 ALERT type 5 Informative message, check

It is advisable to attempt to resolve as many as possible of the alerts in all categories. Often the minor alerts point to easily fixed oversights, errors and omissions in your CIF or refinement strategy, so attention to these fine details can be worthwhile. In order to resolve some of the more serious problems it may be necessary to carry out additional measurements or structure refinements. However, the purpose of your study may justify the reported deviations and the more serious of these should normally be commented upon in the discussion or experimental section of a paper or in the "special_details" fields of the CIF. checkCIF was carefully designed to identify outliers and unusual parameters, but every test has its limitations and alerts that are not important in a particular case may appear. Conversely, the absence of alerts does not guarantee there are no aspects of the results needing attention. It is up to the individual to critically assess their own results and, if necessary, seek expert advice.

Publication of your CIF in IUCr journals

A basic structural check has been run on your CIF. These basic checks will be run on all CIFs submitted for publication in IUCr journals (*Acta Crystallographica*, *Journal of Applied Crystallography*, *Journal of Synchrotron Radiation*); however, if you intend to submit to *Acta Crystallographica Section C* or *E* or *IUCrData*, you should make sure that full publication checks are run on the final version of your CIF prior to submission.

Publication of your CIF in other journals

Please refer to the *Notes for Authors* of the relevant journal for any special instructions relating to CIF submission.

PLATON version of 13/07/2021; check.def file version of 13/07/2021

Datablock e19mkw48 - ellipsoid plot

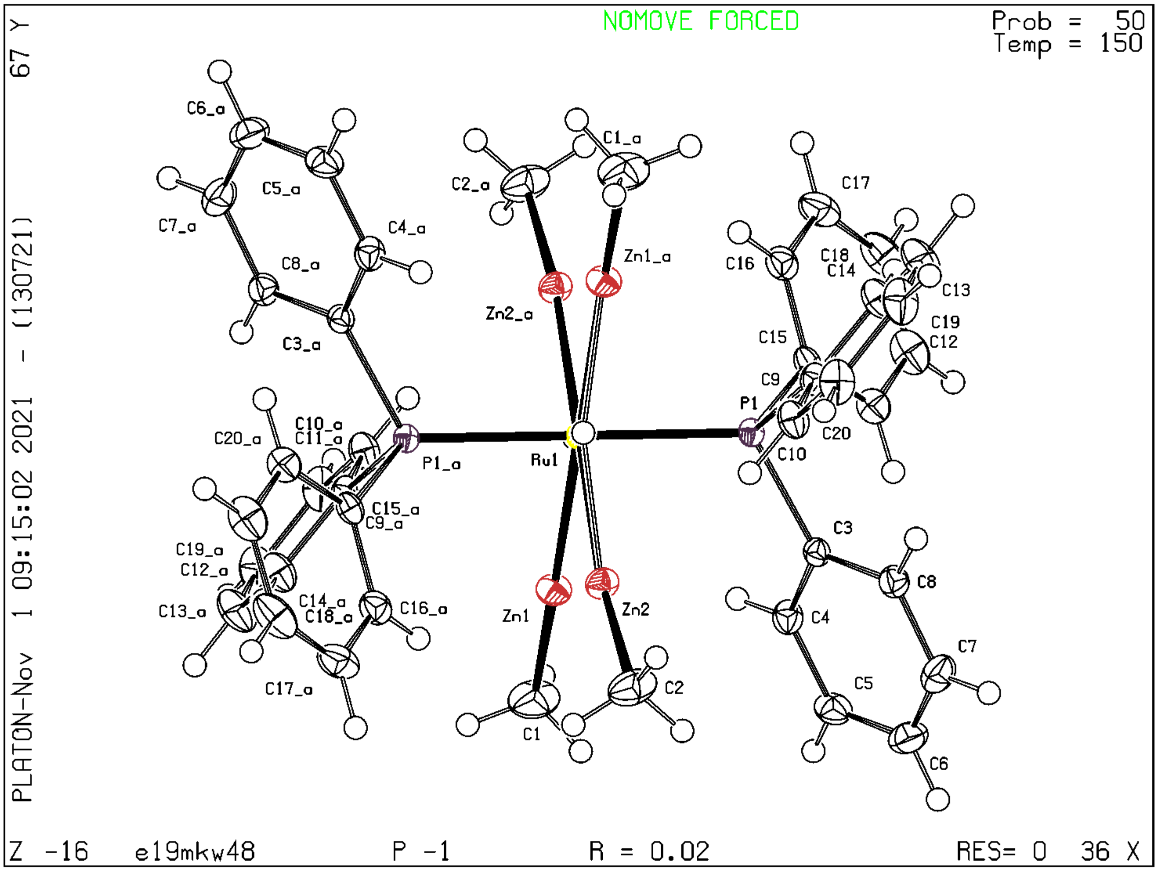

Datablock s19mkw50 - ellipsoid plot

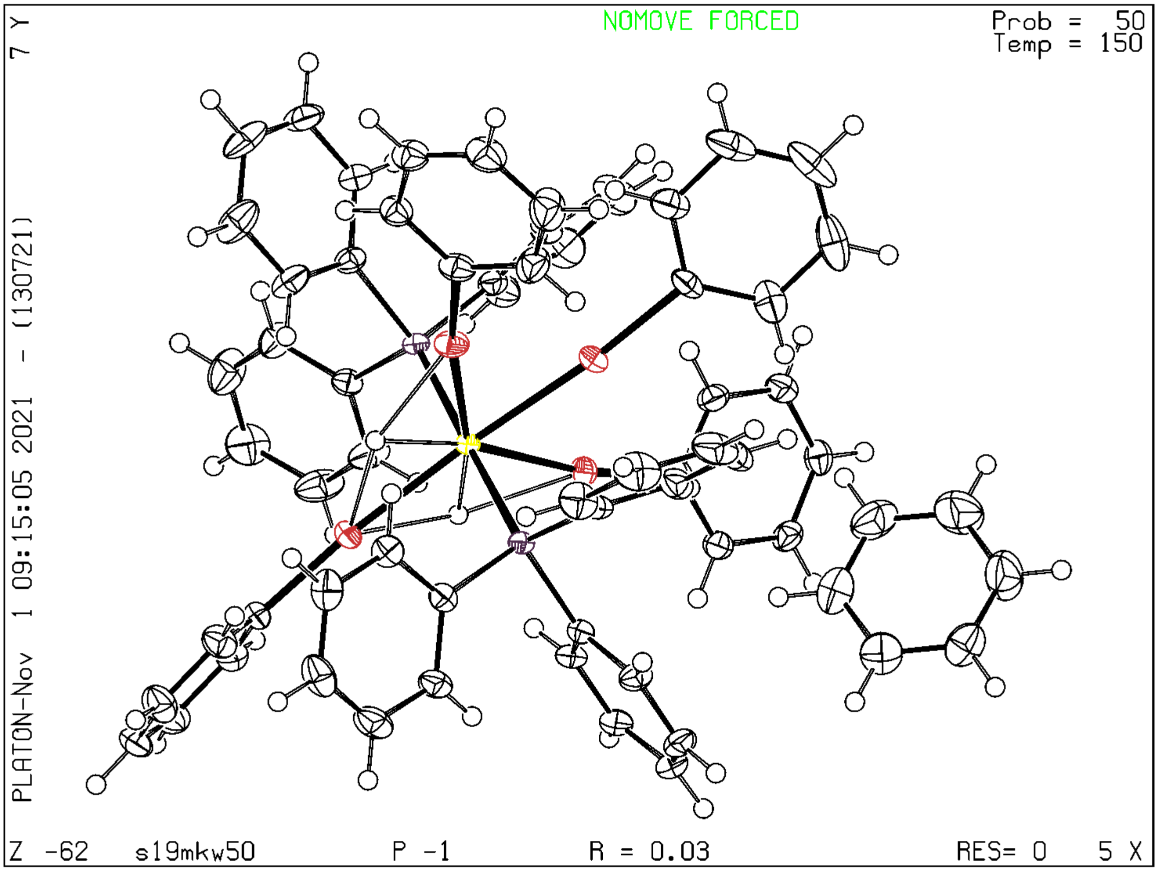

Datablock s19mkw34 - ellipsoid plot

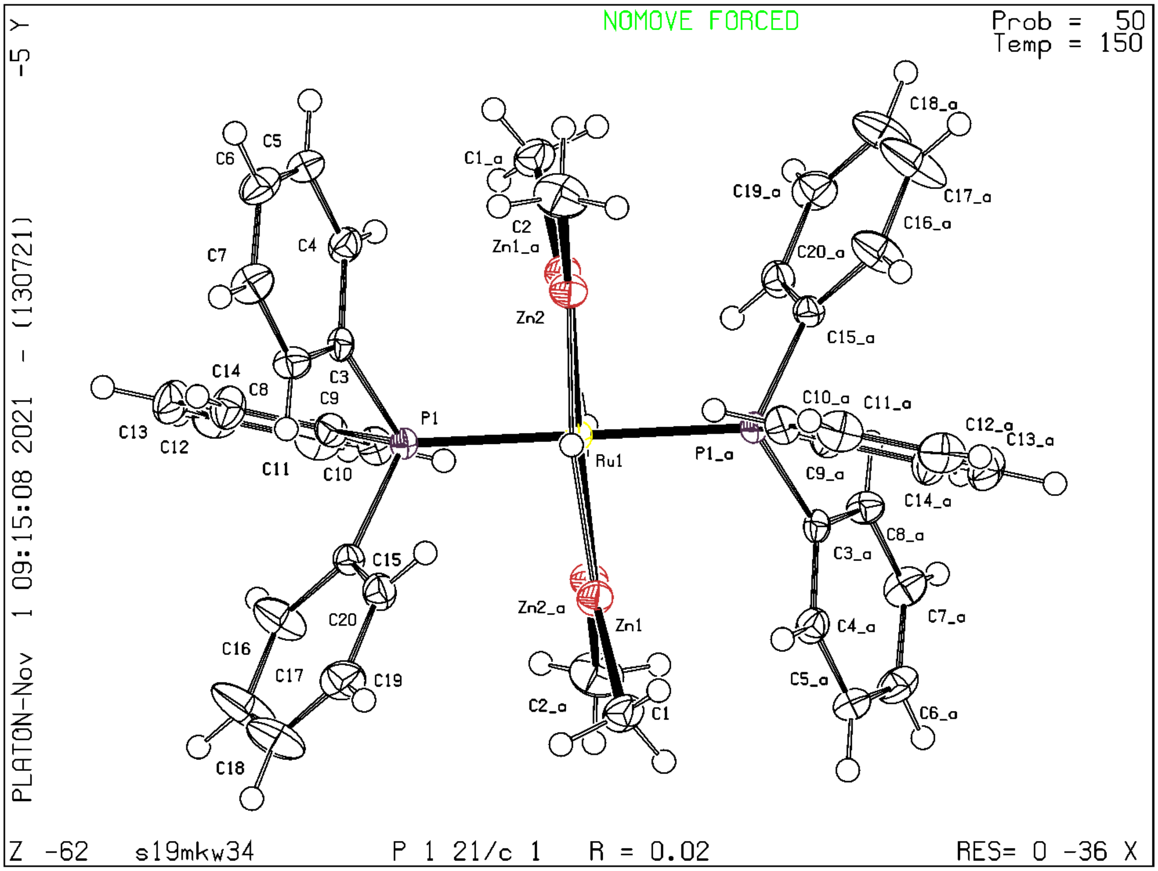

Datablock e19mkw50 - ellipsoid plot

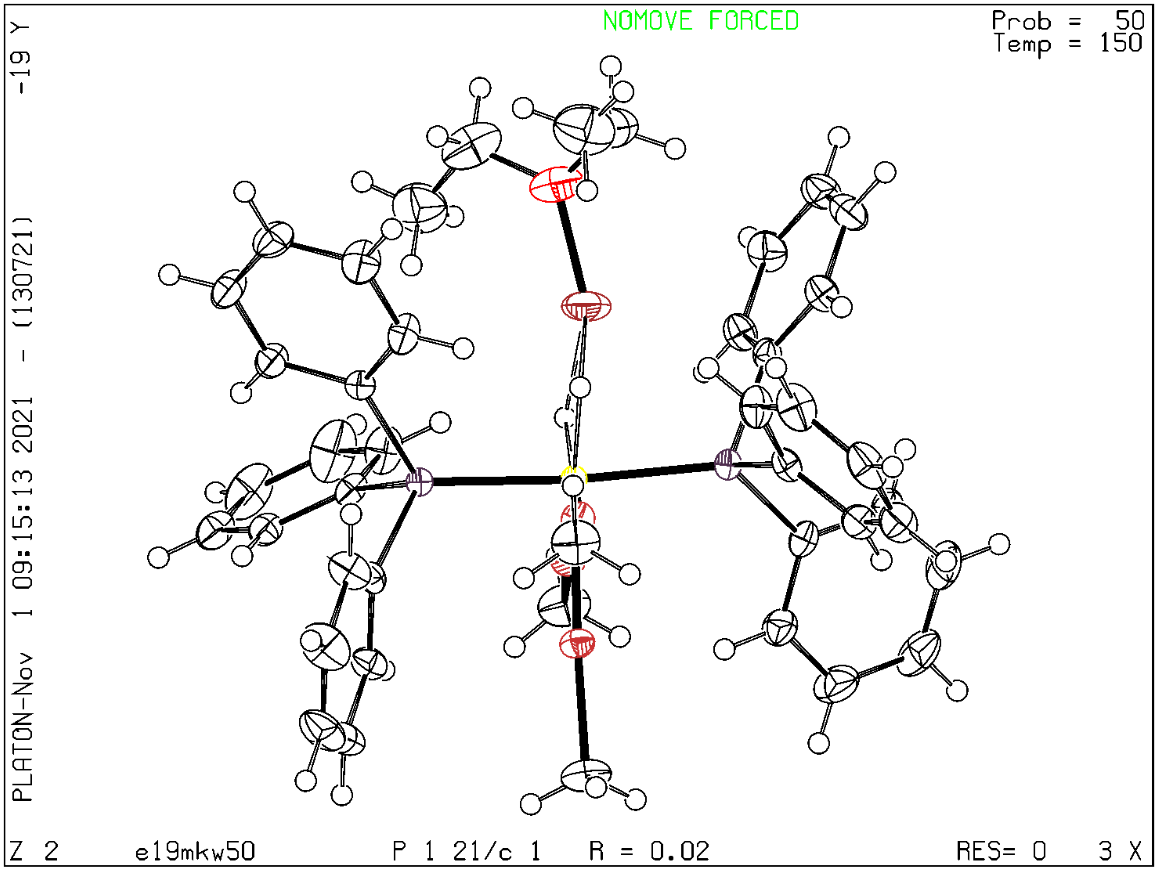

Datablock s20mkw12 - ellipsoid plot

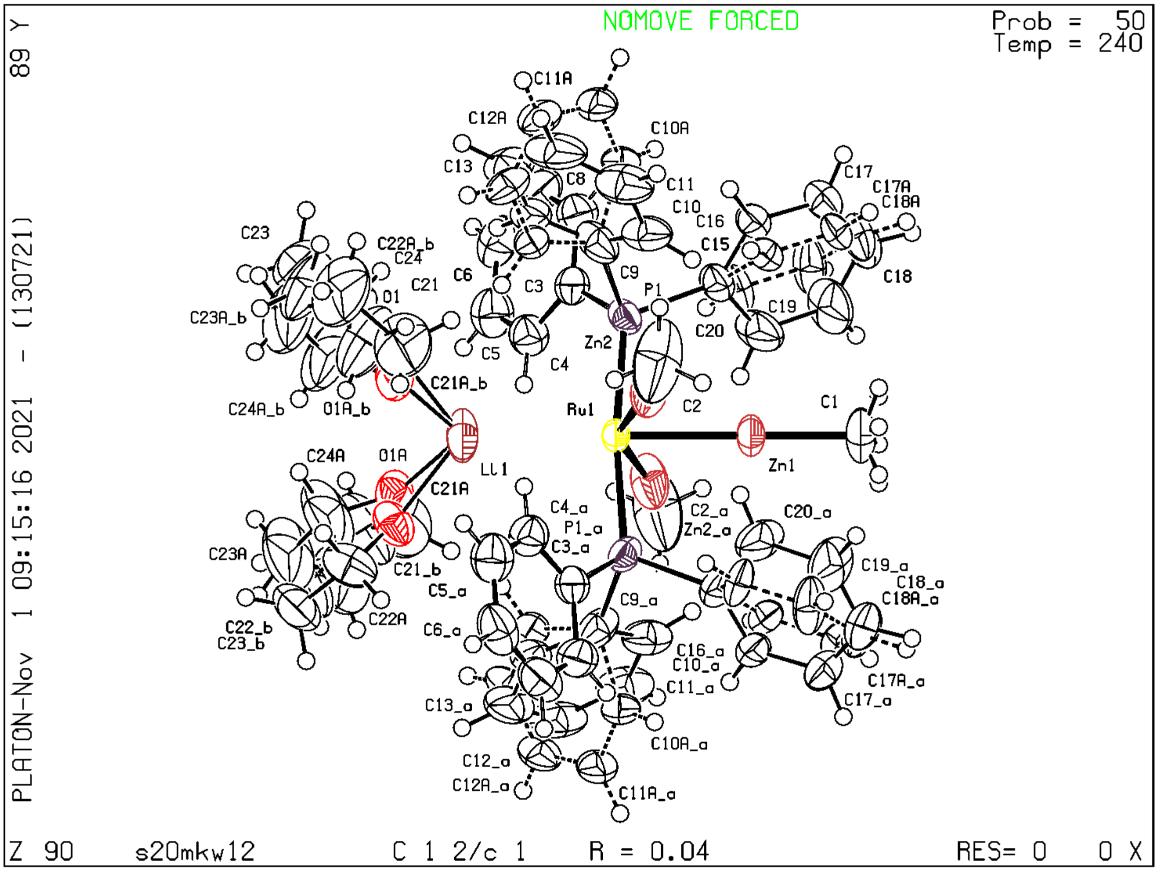

Supplement: Supplementary file 2 — Supporting Information [file ANIE-61-0-s002.pdf]
