# Supplementary material for: Zinc‐Promoted ZnMe/ZnPh Exchange in Eight‐Coordinate [Ru(PPh3)2(ZnMe)4H2]
Source: Angew Chem Int Ed Engl. 2022 Mar 14;61(19):e202117495. doi: 10.1002/anie.202117495 (PMC9311408; doi:10.1002/anie.202117495)
Supplement: Supplementary file 3 — Supporting Information [file ANIE-61-0-s001.pdf]

Angewandte Chemie
Eine Zeitschrift der Gesellschaft Deutscher Chemiker

Supporting Information

Zinc-Promoted ZnMe/ZnPh Exchange in Eight-Coordinate $[Ru(PPh_3)_2(ZnMe)_4H_2]$

L. Sotorrios, F. M. Miloserdov, A.-F. Pécharman, J. P. Lowe, S. A. Macgregor*, M. F. Mahon*, M. K. Whittlesey**

SUPPORTING INFORMATION

S-1 Experimental Procedures and Characterisation S2
S-2 Crystallographic Details..S31
S-3 Computational Details..S36
S-4 References……………………………………………………………………..S70

S-1 Experimental Procedures and Characterisation

General Comments. All manipulations were carried out at room temperature under argon using standard Schlenk, high vacuum and glovebox techniques using dry and degassed solvents unless otherwise stated. C_6H_5F was additionally dried over $LiAlH_4$ before use. [D_6]-benzene, [D_8]-THF and [D_8]-toluene were vacuum transferred from potassium. NMR spectra were recorded at 298 K (unless otherwise stated) on Bruker Avance 400 and 500 MHz or Agilent ProPlus 500 MHz NMR spectrometers and referenced as follows: [D_6]-benzene (^1H, δ = 7.16 ppm; ^{13}C, δ = 128.0 ppm), [D_8]-THF (^1H, δ = 3.58 ppm; ^{13}C, δ = 67.6 ppm), [D_8]-toluene (^1H, δ = 2.09). $^{31}P\{^1H\}$ spectra were referenced externally to 85% H_3PO_4 (δ = 0.0 ppm). Elemental analyses were performed by Elemental Microanalysis Ltd, Okehampton, Devon, U.K. [$Ru(PPh_3)_3HCl$]·toluene and [$Ru(PPh_3)(Ph_2PC_6H_4)_2(ZnMe)_2$] were prepared according to literature methods.[1,2] Prior to use, [$Ru(PPh_3)_3HCl$]·toluene was dried under high vacuum and ground to a fine powder affording a material with *ca.* 1 molecule of toluene per Ru (^1H NMR analysis). $LiCH_2TMS$ was used as a colourless solid and obtained by cooling a commercial 1.0 M solution in pentane at -32 °C, separating the resulting colourless crystals by decantation and drying under vacuum.

[$Ru(PPh_3)_2(ZnMe)_4H_2$] (1a).‡ To an agitated suspension of [$Ru(PPh_3)_3HCl$]·toluene (305 mg, 0.30 mmol) in C_6H_5F (3.0 mL) was added $LiCH_2TMS$ (57 mg, 0.60 mmol). After 5 min, all of the purple starting material had dissolved to give an orange suspension, which was treated with $ZnMe_2$ (3 mL of 2.0 M toluene solution, 6.0 mmol)§ before a second portion of [$Ru(PPh_3)_3HCl$]·toluene (300 mg, 0.30 mmol) was added at stirring. The resulting red solution was degassed (3 x freeze-pump-thaw cycles) and placed under 1 atm H_2 while frozen.$ The reaction mixture was allowed to melt and then vigorously agitated while opened to an H_2 atmosphere and warmed to room temperature over ca 10 min. It was then stirred intensively for 30 min, yielding a yellow precipitate in the process. After stirring for an

additional 1.5 h at room temperature, the reaction mixture was cooled at 0 ºC (for ca 1 h) and the yellow solid isolated by cannula filtration. The solid was dissolved in 12 mL of hot C_6H_5F and filtered. The residual LiCl was washed with C_6H_5F (1 mL) and the filtrate and washings combined and left to crystallise at room temperature (for ca. 3 h), and then at -35 ºC for ca. 48 h. The yellow crystalline product was isolated while cold, washed with hexane (2 x 2 mL) and dried under vacuum. Yield: 355 mg (62%). ^1H NMR (400 MHz, [D$_6$]-benzene): δ = 7.88-7.80 (m, 12H, PPh_3), 7.08-7.02 (m, 12H, PPh_3), 6.95 (t, J = 7.2 Hz, 6H, PPh_3), -0.47 (s, 12H, $ZnCH_3$), -8.55 (t, ^2J(H,P) = 13.7 Hz, 2H, RuH) ppm; $^{31}P\{^1H\}$ NMR (162 MHz, [D$_6$]-benzene): δ = 57.7 (s) ppm; $^{13}C\{^1H\}$ NMR (101 MHz, [D$_6$]-benzene): δ = 144.8 (virtual triplet (vt), J = 21 Hz, *ipso*-C PPh_3), 132.5 (vt, J = 6 Hz, *ortho*-C PPh_3), 129.4 (s, *para*-C PPh_3), 128.9 (vt, J = 4 Hz, *meta*-C PPh_3), 4.7 (br s, $ZnCH_3$; confirmed by HSQC) ppm; elemental analysis (%) calcd for $C_{40}H_{44}P_2RuZn_4$: C 50.61, H 4.67; found: C 50.86, H 4.64.

‡The scheme below is intended to provide insight on the synthetic procedure. The 0.5 equiv of [Ru(PPh_3)$_3$HCl]·toluene used initially reacts with $LiCH_2TMS$ to afford [Ru(PPh_3)($C_6H_4PPh_2$)$_2$H][Li][3] (plus $SiMe_4$). The second 0.5 equiv of [Ru(PPh_3)$_3$HCl]·toluene reacts with some of the $ZnMe_2$ to afford [Ru(PPh_3)($C_6H_4PPh_2$)$_2$H(ZnMe)],[3] releasing ZnMeCl which can react with [Ru(PPh_3)($C_6H_4PPh_2$)$_2$H][Li] to eliminate LiCl, allowing complete conversion of all the Ru to [Ru(PPh_3)($C_6H_4PPh_2$)$_2$H(ZnMe)]. In the presence of **both** $ZnMe_2$ and H_2 (see $ below), this readily yields **1a**.

Steps **1** and **2**:

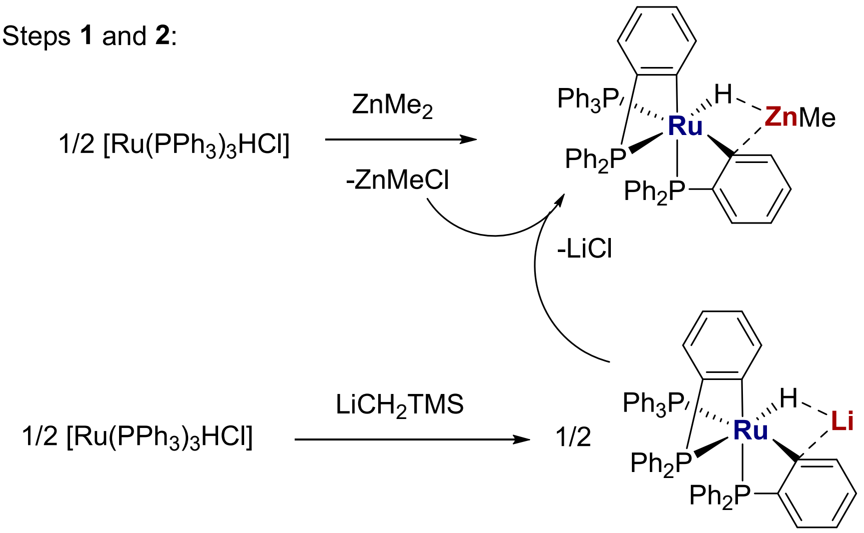

§ The excess of $ZnMe_2$ and its high concentration are required to prevent the formation of $[Ru(PPh_3)_3H_3(ZnMe)]$,[3] which could not be further converted into **1a** upon treatment with excess of $ZnMe_2$.

$ It is important to add H_2 within 1 h after addition of $ZnMe_2$. A longer delay can lead to the formation of significant quantities of $[Ru(PPh_3)(Ph_2PC_6H_4)_2(ZnMe)_2]$.[2] Although this can be transformed into **1a** upon addition of H_2 and excess of $ZnMe_2$, it is a much slower process. Thus, reaction of $[Ru(PPh_3)(Ph_2PC_6H_4)_2(ZnMe)_2]$ (11 mg, 0.01 mmol) with H_2 (1 atm) at 80 °C for 4 h with subsequent addition of $ZnMe_2$ (50 µL of 2.0 M toluene solution, 0.1 mmol) gave **1a** in ca. 60% NMR yield over 3 weeks at room temperature.

$[Ru(PPh_3)_2(ZnPh)_4H_2]$ (2b). To an agitated solution of **1a** (95 mg, 0.1 mmol) in C_6H_6 (3 mL) was added $ZnPh_2$ (55 mg, 0.25 mmol, 2.5 equiv). After 30 min, the reaction mixture was reduced to dryness (to remove eliminated $ZnMe_2$) and the residue redissolved in C_6H_6 (4 mL). Reduction to dryness again, followed by redissolution in 4 mL C_6H_6 was then followed by filtration of the benzene solution through a pad of Celite®. The pad was washed with benzene (1 mL), the filtrate and benzene washings combined, treated with hexane (8 mL) and left to crystallise at room temperature. The pale yellow crystalline product was separated by decantation, washed with hexane (2 x 1 mL) and dried under vacuum. Yield: 89 mg (72%). ^1H NMR (500 MHz, [D8]-THF): δ = 7.79-7.74 (m, 12H, PPh_3), 7.27-7.19 (m,

18H, PPh_3), 7.01-6.93 (m, 12H, ZnPh) 6.89-6.83 (m, 8H, ZnPh), -8.24 (t, ^2J(H,P) = 13.4 Hz, 2H, RuH) ppm; $^{31}P\{^1H\}$ NMR (202 MHz, [D_8]-THF): δ = 53.4 (s) ppm; $^{13}C\{^1H\}$ NMR (126 MHz, [D_8]-THF): δ 161.9 (*ipso*-C ZnPh; assignment confirmed by HMBC correlation to ^1H signals at δ = -8.24 and 6.88 ppm), 145.6 (vt, *J* = 21 Hz, *ipso*-C PPh_3), 136.2 (s, ZnPh), 133.2 (vt, *J* = 6 Hz, *ortho*-C PPh_3), 130.3 (s, Ph), 129.8 (vt, *J* = 4 Hz, *meta*-C PPh_3), 127.5 (s, ZnPh), 127.2 (s, ZnPh) ppm; elemental analysis (%) calcd for $C_{60}H_{52}P_2RuZn_4{\cdot}0.5C_6H_6$: C 61.19, H 4.48; found: C 61.39, H 4.57.

[Ru(PPh_3)$_2$(ZnMe)$_3$\{Li(OEt_2)\}H_2] (3). MeLi (150 µL of 2.0 M Et_2O solution, 0.3 mmol) was added to an agitated suspension of **1a** (95 mg, 0.1 mmol) in Et_2O (2 mL). The resulting orange suspension was stirred for 5 min. Hexane (3 mL) was added and the sample then left to crystallise at room temperature (1 h), and then at -32 °C (20 h). The resulting yellow crystalline product was separated by decantation, washed with hexane (2 x 2 mL) and dried under vacuum. Yield: 78 mg (84%). Due to the poor solubility of the compound in [D_6]-benzene, NMR characterisation was performed in 1:2 [D_6]-benzene/Et_2O mixture (referenced to [D_5]-benzene at δ = 7.16 ppm). Although yellow in the solid state, dissolution in [D_6]-benzene/Et_2O afforded an orange solution. ^1H NMR (400 MHz, 1:2 [D_6]-benzene/Et_2O): δ = 7.84-7.77 (m, 12H, PPh_3), 7.12-7.00 (m, 18H, PPh_3), -1.00 (s, 9H, ZnMe), -10.69 (br t, ^2J(H,P) = 17.0 Hz, 2H, RuH) ppm; $^{31}P\{^1H\}$ NMR (202 MHz, 1:2 [D_6]-benzene/Et_2O): δ = 66.9 (s) ppm; $^7Li\{^1H\}$ NMR (194 MHz, 1:2 [D_6]-benzene/Et_2O): δ = 1.7 (br s) ppm; elemental analysis (%) calcd for $C_{39}H_{41}P_2RuZn_3Li$: C 53.48, H 4.72; found: C 53.57, H 4.99. Crystallisation of **3** from THF-hexane gave orange block crystals of [Ru(PPh_3)$_2$(ZnMe)$_3$\{Li(THF)$_2$\}H_2], which were analysed by X-ray crystallography (Figure S24).

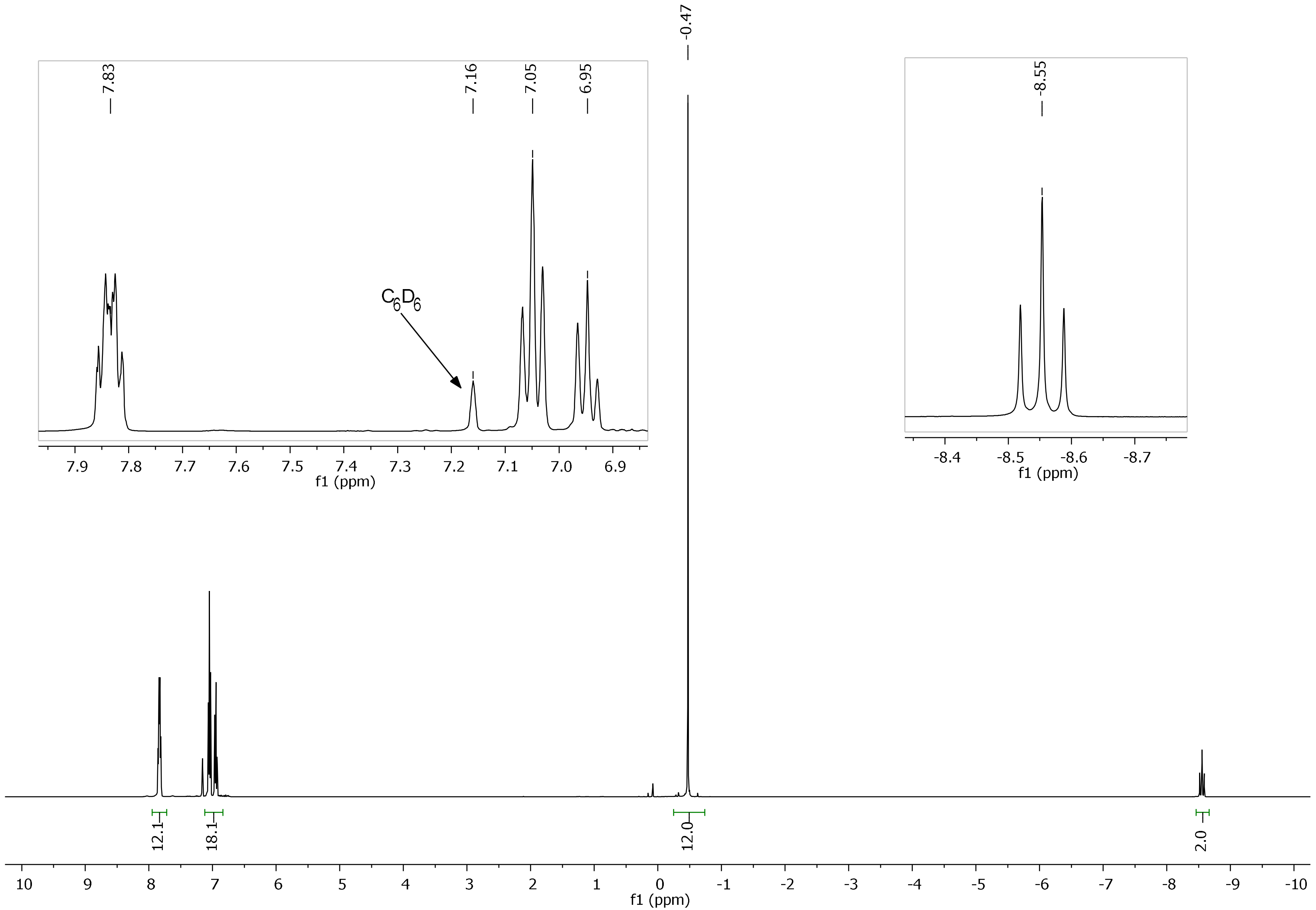

Figure S1. ^{1}H NMR spectrum (400 MHz, [D_6]-benzene, 298 K) of [$Ru(PPh_3)_2(ZnMe)_4H_2$] (**1a**).

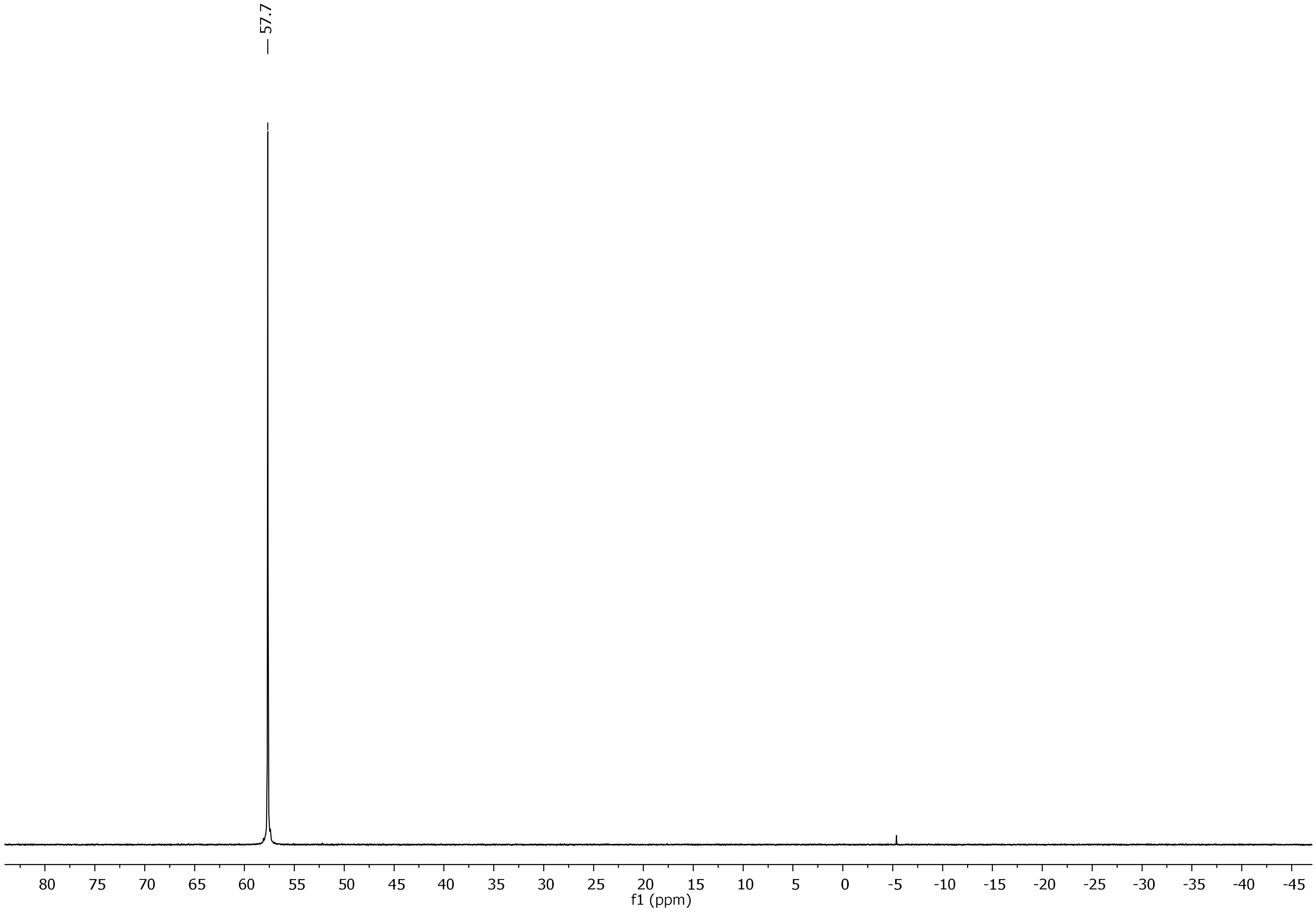

Figure S2. $^{31}P\{^1H\}$ NMR spectrum (162 MHz, [D_6]-benzene, 298 K) of [$Ru(PPh_3)_2(ZnMe)_4H_2$] (**1a**).

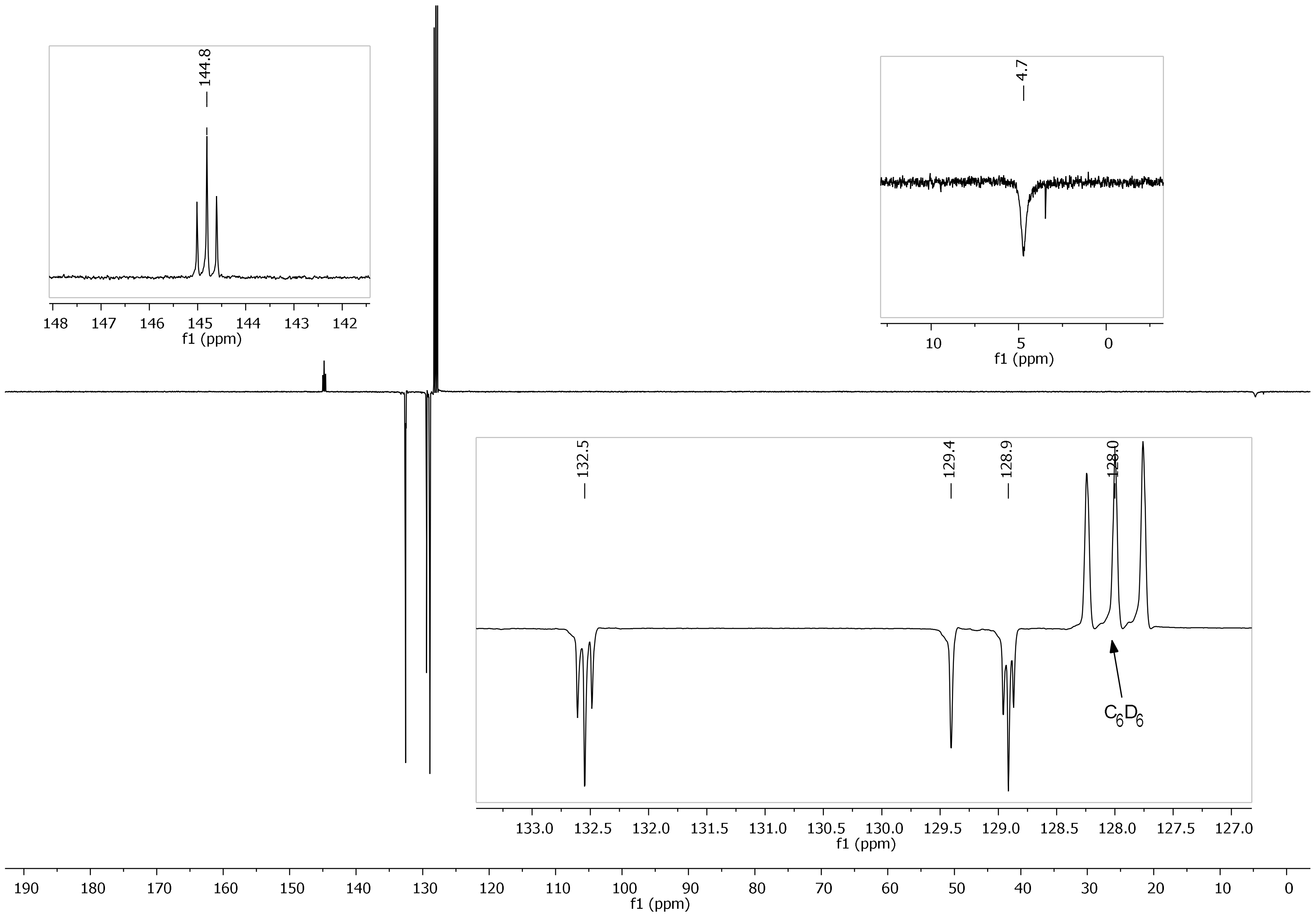

Figure S3. $^{13}C\{^1H\}$ DEPTQ NMR spectrum (101 MHz, [D$_6$]-benzene, 298 K) of [Ru(PPh$_3$)$_2$(ZnMe)$_4$H$_2$] (**1a**).

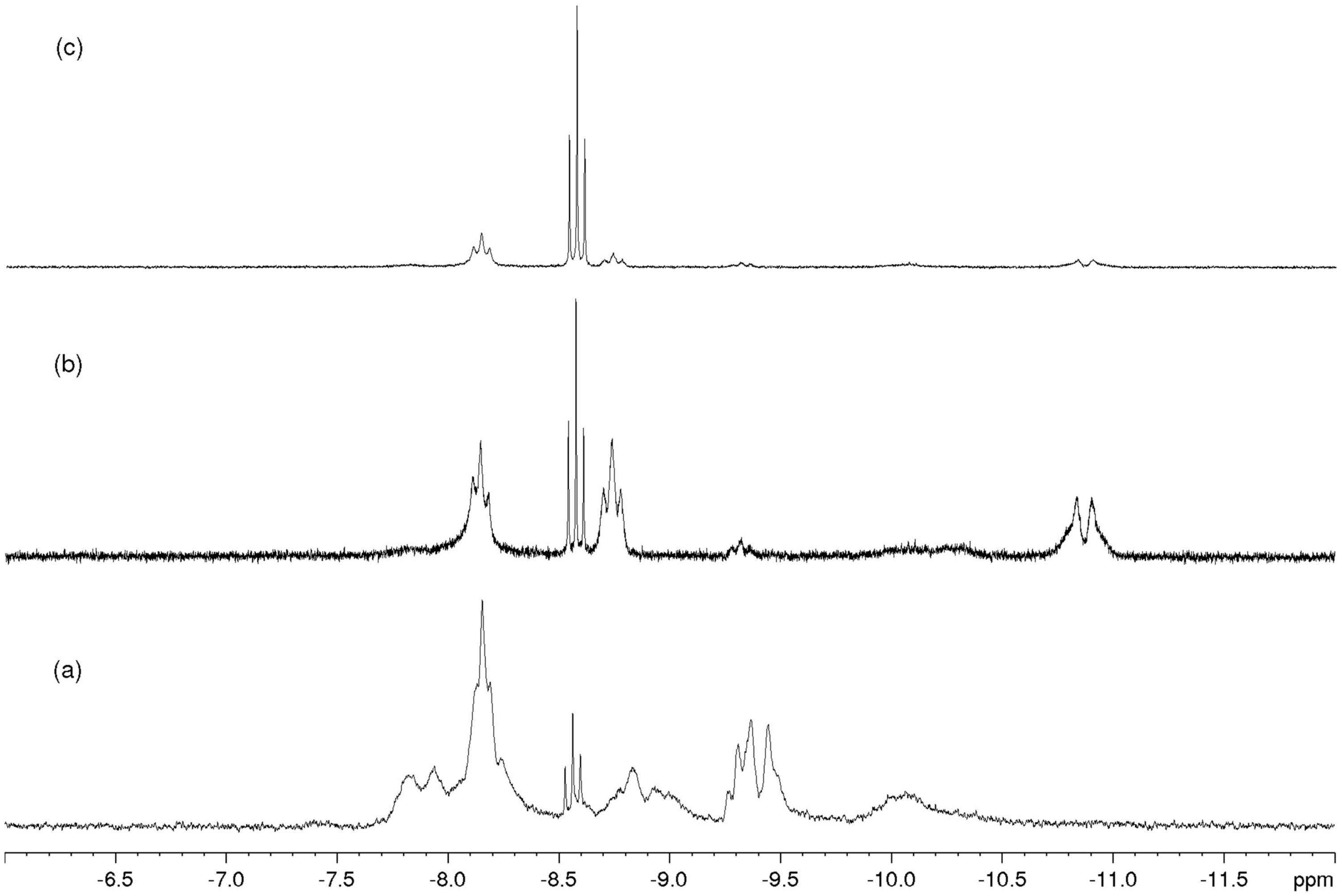

Figure S4. Hydride region of the ^{1}H NMR spectra (400 MHz, [D_6]-benzene, 298 K) from reaction of (a) [Ru(PPh_3)($Ph_2PC_6H_4$)$_2$(ZnMe)$_2$] with H_2 (1 atm) to afford multiple products versus formation of [Ru(PPh_3)$_2$(ZnMe)$_4$$H_2$] (**1a**) upon addition of H_2 (1 atm) and 10 eq $ZnMe_2$ to [Ru(PPh_3)($Ph_2PC_6H_4$)$_2$(ZnMe)$_2$] after (b) ca. 20 h and (c) 3 weeks.

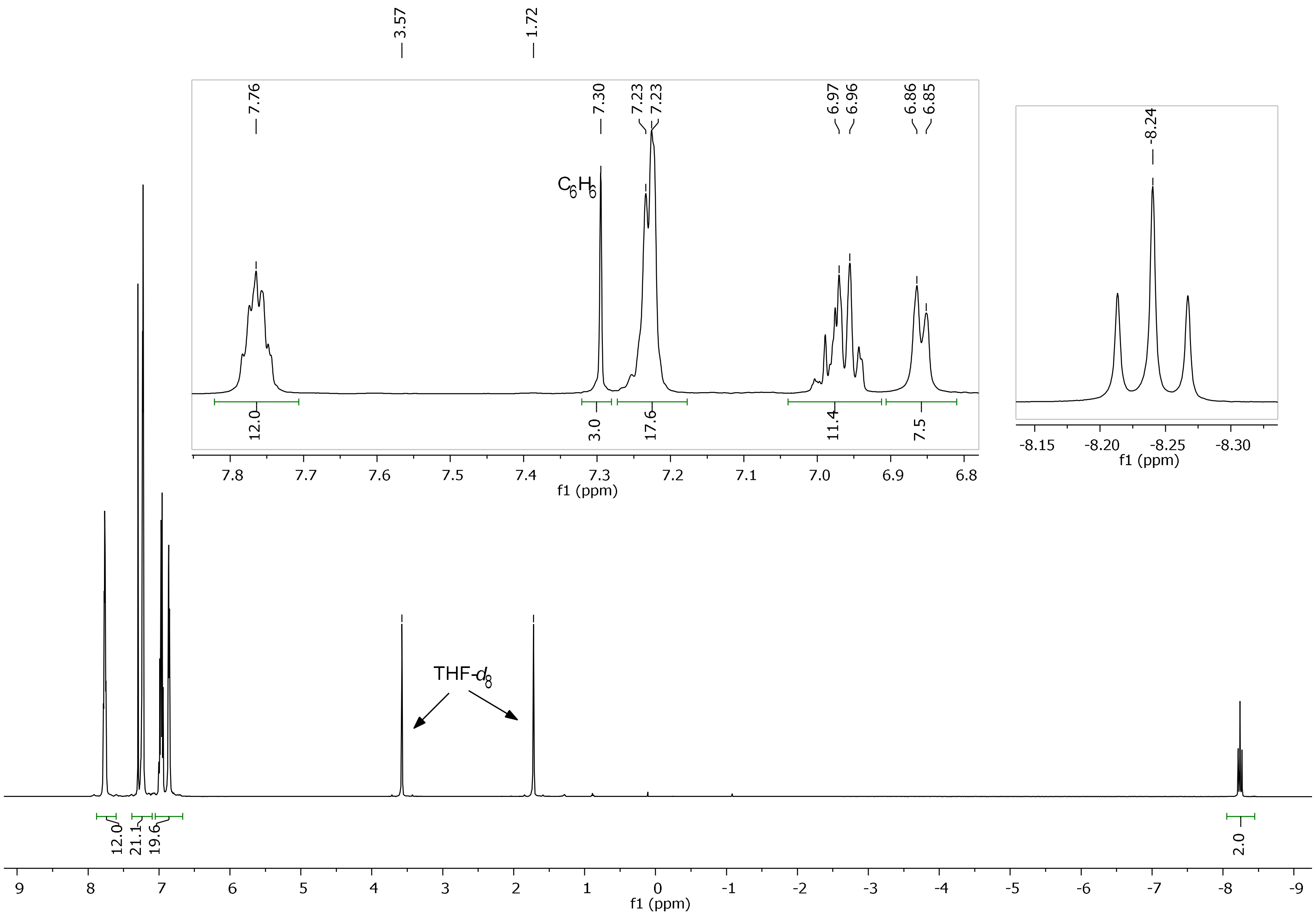

Figure S5. ^{1}H NMR spectrum (500 MHz, [D_8]-THF, 298 K) of [$Ru(PPh_3)_2(ZnPh)_4H_2$] (**2b**).

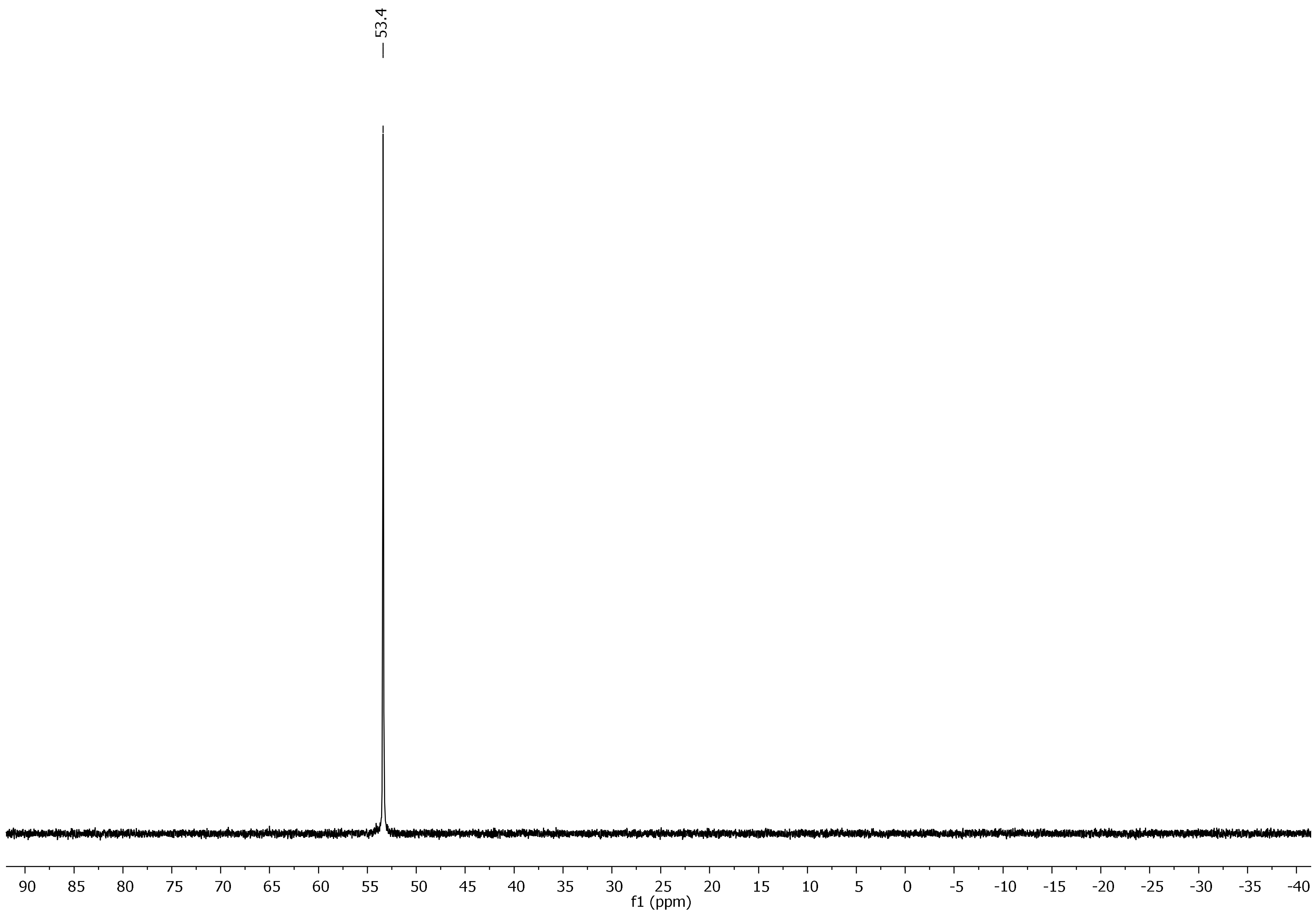

Figure S6. $^{31}P\{^1H\}$ NMR spectrum (202 MHz, [D$_8$]-THF, 298 K) of [Ru(PPh$_3$)$_2$(ZnPh)$_4$H$_2$] (**2b**).

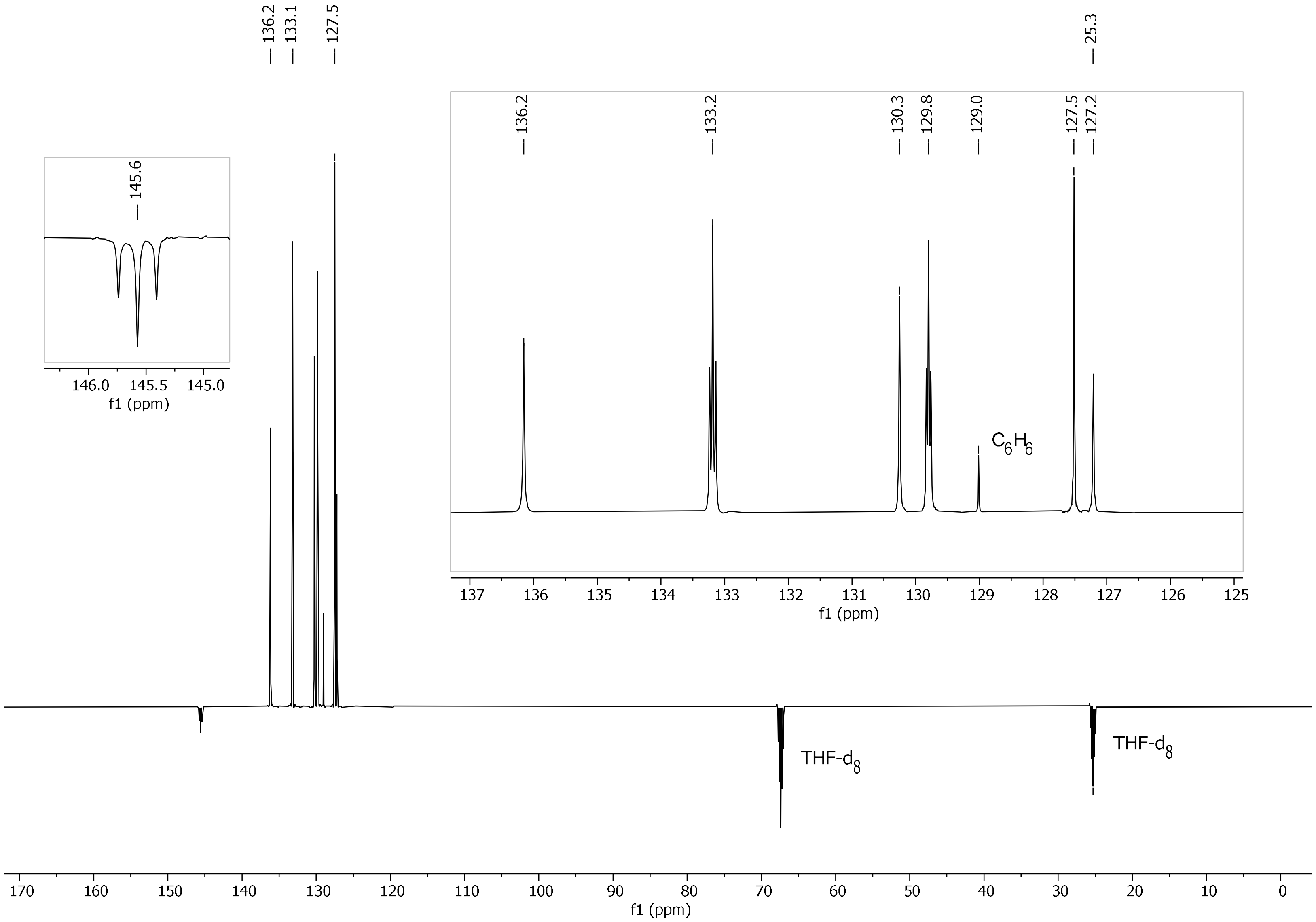

Figure S7. $^{13}C\{^{1}H\}$ PENDANT NMR spectrum (126 MHz, [D_8]-THF, 298 K) of [$Ru(PPh_3)_2(ZnPh)_4H_2$] (**2b**).

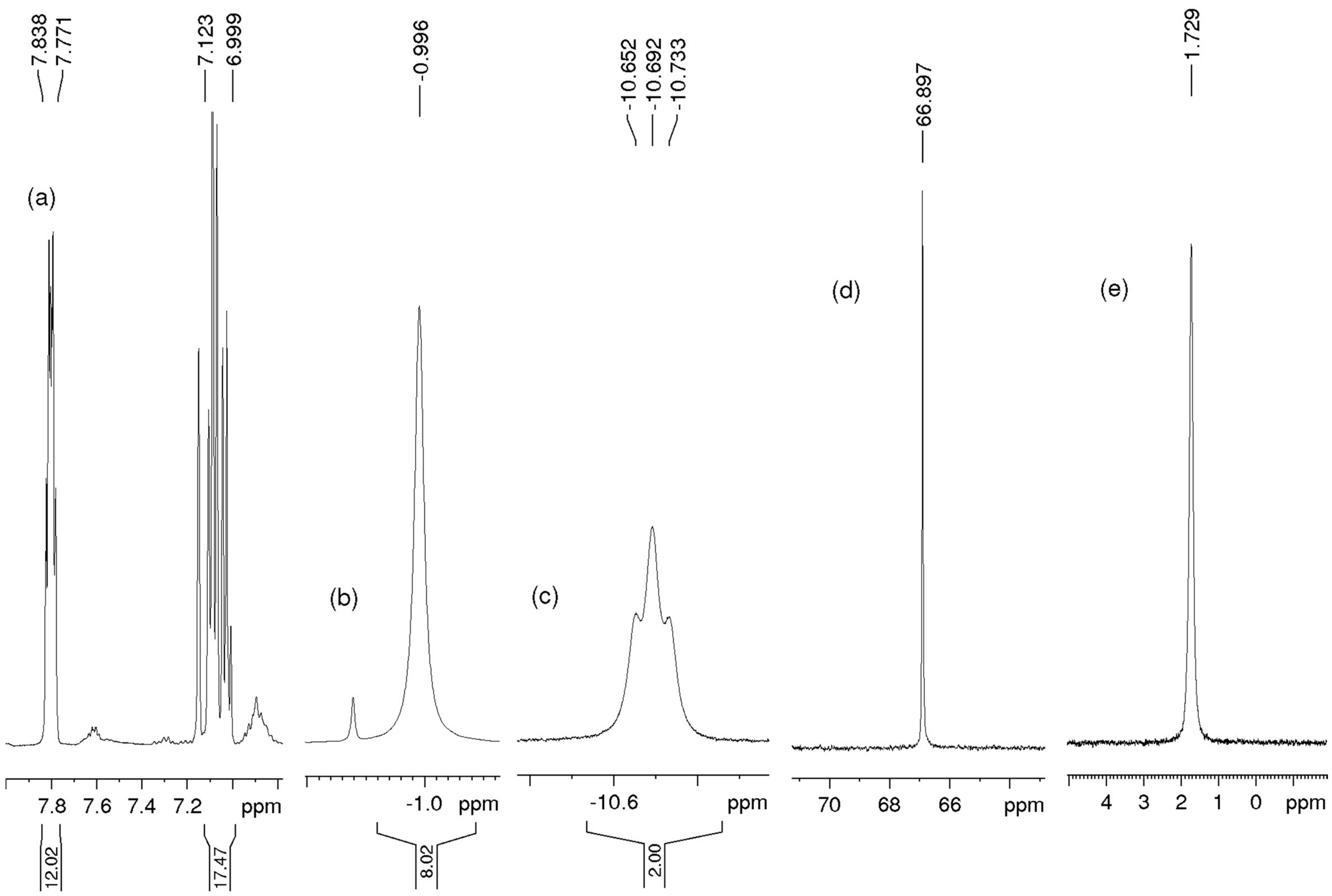

Figure S8. Selected (a-c) ^{1}H (400 MHz), (d) $^{31}P\{^{1}H\}$ (202 MHz) and (e) $^{7}Li\{^{1}H\}$ (194 MHz) NMR spectra (1:2 [D$_6$]-benzene:Et$_2$O, 298 K) of [Ru(PPh$_3$)$_2$(ZnMe)$_3${Li(OEt$_2$)}H$_2$] (**3**).

Variable temperature NMR study of 1a and 2b. 1a (10 mg, 0.01 mmol) was dissolved in [D_8]-toluene or [D_8]-THF (0.5 mL). Variable temperature NMR analysis showed an exchange process between two isomers **1a** and **1b**, with **1a** being the major isomer (at 193 K, ca. 2.7:1 in [D_8]-THF; at 199 K, ca. 3.2:1 in [D_8]-toluene). Similar measurements on **2b** (12 mg, 0.01 mmol) showed the presence of isomers **2a** and **2b**; the latter was identified as the major isomer (at 193 K, ca. 1:3.2 ratio in [D_8]-THF). The rate constants for exchange were obtained from individual fitting of $^{31}P\{^1H\}$ NMR spectra over the temperature range 298-223 K using g-NMR (version 3.6.5). See Figures S14, S17 and S20. Values of $\Delta H^‡$, $\Delta S^‡$ and $\Delta G^‡$ derived from the slopes and intercepts are summarised in Table S1.

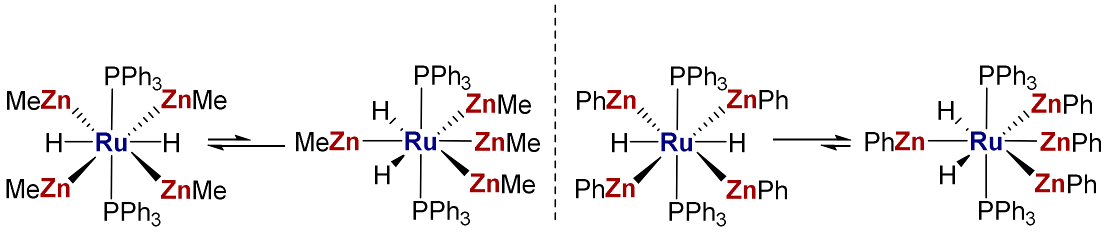

1a (major): Selected ^1H NMR (400 MHz, [D_8]-toluene, 190 K): δ = -0.37 (s, 12H, $ZnCH_3$), -8.31 (t, ^2J(H,P) = 14.4 Hz, 2H, RuH) ppm. Selected ^1H NMR (400 MHz, [D_8]-THF, 179 K): δ = -1.16 (s, $ZnCH_3$)*, -8.66 (t, ^2J(H,P) = 14.4 Hz, 2H, RuH) ppm; $^{31}P\{^1H\}$ NMR (162 MHz, [D_8]-toluene, 190 K): δ = 57.1 (s) ppm; $^{31}P\{^1H\}$ NMR (162 MHz, [D_8]-THF, 179 K): δ = 56.8 (s) ppm. *Overlaps with signal of **1b**.

1b (minor): Selected ^1H NMR (400 MHz, [D_8]-toluene, 190 K): δ = -0.24 (s, 6H, $ZnCH_3$), -0.30 (s, 3H, $ZnCH_3$), -0.44 (s, 3H, $ZnCH_3$), -8.38 (t, ^2J(H,P) = 13.0 Hz, 2H, RuH) ppm. Selected ^1H NMR (400 MHz, [D_8]-THF, 179 K): δ = -1.07 (br s, 3H, $ZnCH_3$), -1.16 (s, $ZnCH_3$)*, -1.27 (br s, 3H, $ZnCH_3$), -8.98 (br t, ^2J(H,P) = 13.0 Hz, 2H, RuH) ppm; $^{31}P\{^1H\}$ NMR (162 MHz, [D_8]-toluene, 190 K): δ = 57.8 (s) ppm; $^{31}P\{^1H\}$ NMR (162 MHz, [D_8]-THF, 179 K): δ = 58.2 (s) ppm. *Overlaps with signal of **1a**.

2a (minor): Selected ^{1}H NMR (400 MHz, [D$_8$]-THF, 193 K): δ = -7.99 (t, ^2J(H,P) = 14.2 Hz, 2H, RuH) ppm. Selected ^{1}H NMR (500 MHz, [D$_8$]-THF, 233 K): δ = -8.09 (br t, ^2J(H,P) = 13.6 Hz, 2H, RuH) ppm; ^{31}P{^{1}H} NMR (162 MHz, [D$_8$]-THF, 193 K): δ = 55.2 (s) ppm; Selected ^{13}C{^{1}H} NMR (126 MHz, [D$_8$]-THF, 233 K): δ = 161.7 (s, confirmed by HMBC, C_{ipso} of ZnPh), 146.3 (vt, J = 21 Hz, C_{ipso} of PPh_3) ppm.

2b (major): Selected ^{1}H NMR (400 MHz, [D$_8$]-THF, 193 K): δ = -8.18 (t, ^2J(H,P) = 13.3 Hz, 2H, RuH) ppm. Selected ^{1}H NMR (500 MHz, [D$_8$]-THF, 233 K): δ = -8.19 (br t, ^2J(H,P) = 13.2 Hz, 2H, RuH) ppm; ^{31}P{^{1}H} NMR (162 MHz, [D$_8$]-THF, 193 K): δ = 55.9 (s) ppm; Selected ^{13}C{^{1}H} NMR (126 MHz, [D$_8$]-THF, 233 K): δ = 166.2 (observed in HMBC, C_{ipso} of ZnPh), 162.4 (observed in HMBC, C_{ipso} of ZnPh), 158.2 (observed in HMBC, C_{ipso} of ZnPh), 145.7 (vt, J = 20 Hz, C_{ipso} of PPh_3) ppm.

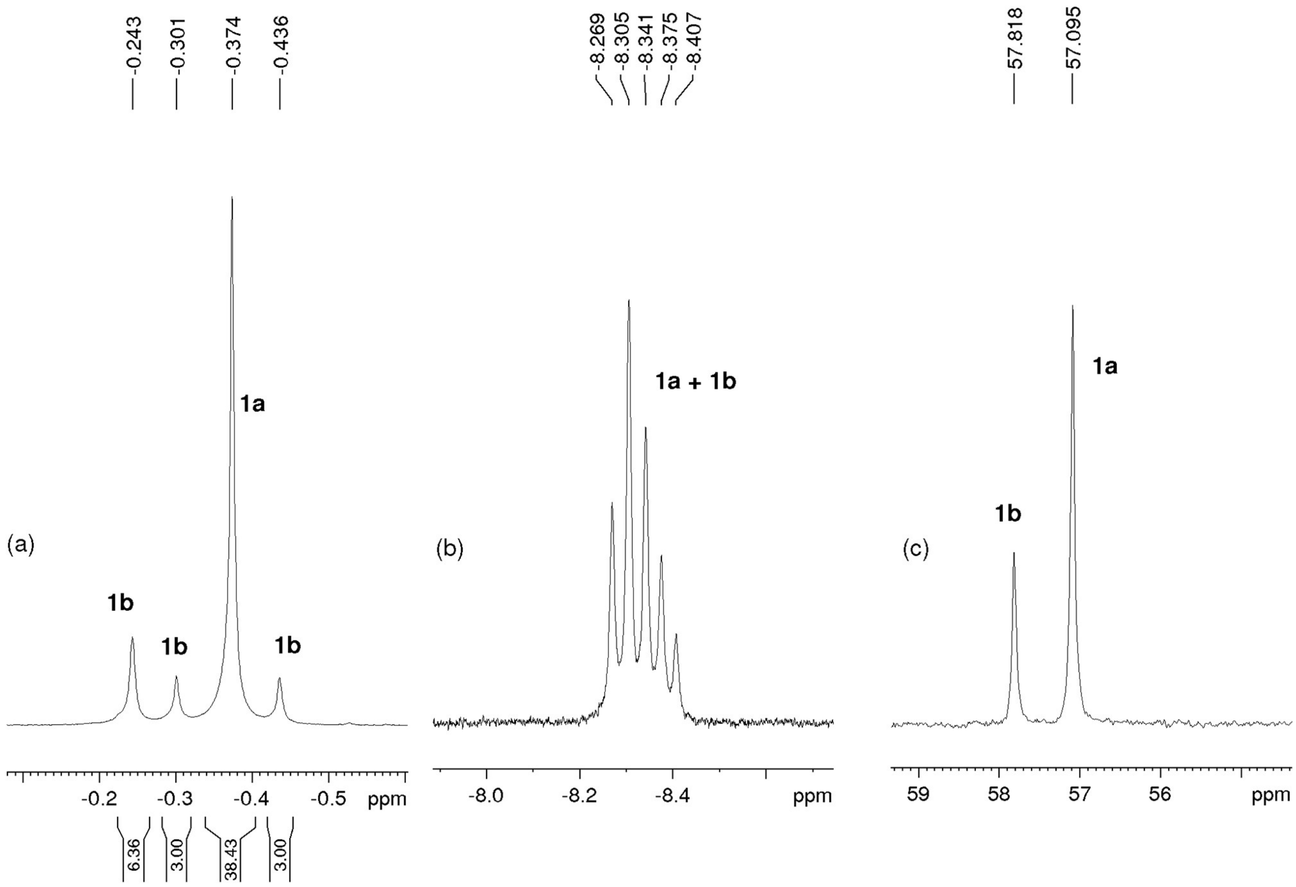

Figure S9. (a) ZnMe and (b) RuH regions of the ^{1}H NMR spectrum (400 MHz, [D$_8$]-toluene, 190 K) and (c) ^{31}P{^{1}H} NMR spectrum (162 MHz, [D$_8$]-toluene, 190 K) of **1a** and **1b**.

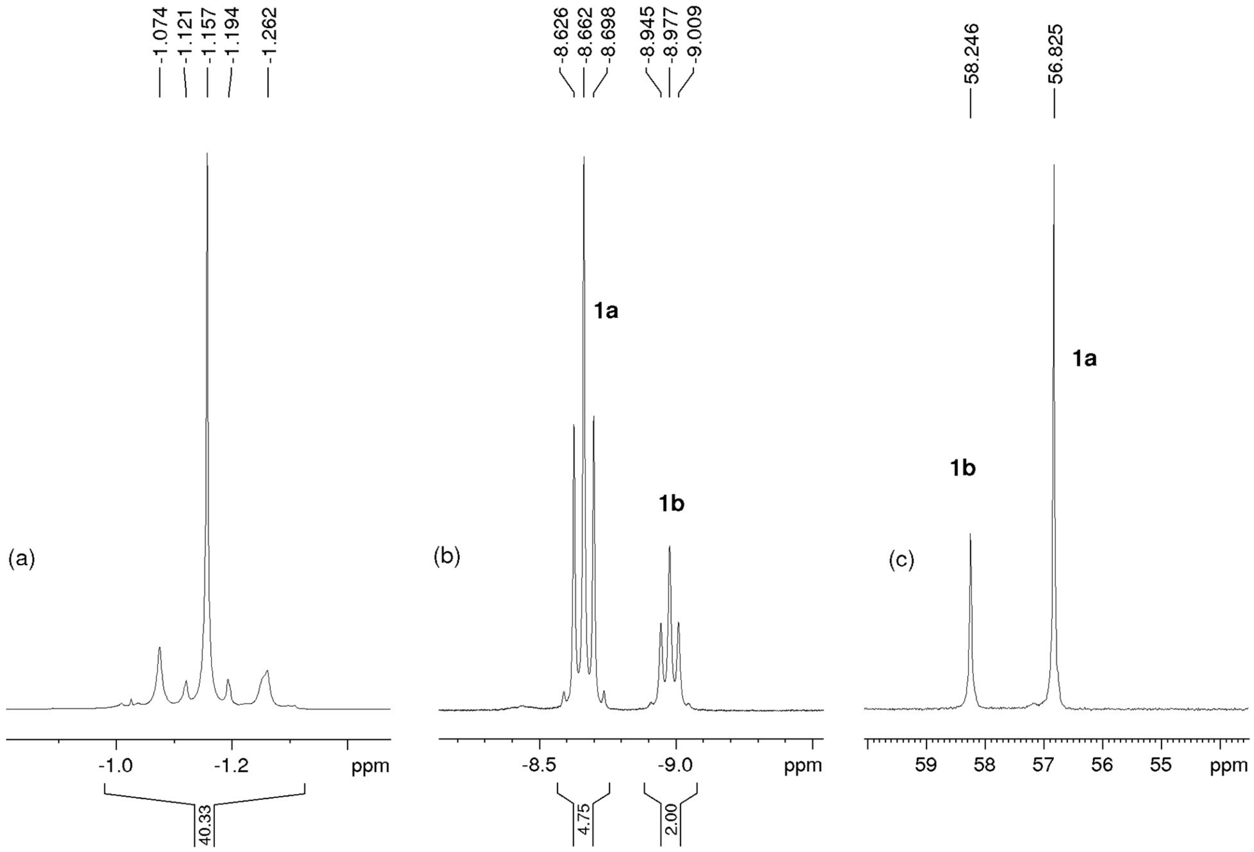

Figure S10. (a) ZnMe and (b) RuH regions of the ^{1}H NMR spectrum (400 MHz, [D8]-THF, 178 K) and (c) $^{31}P\{^{1}H\}$ NMR spectrum (162 MHz, [D8]-THF, 178 K) of **1a** and **1b**.

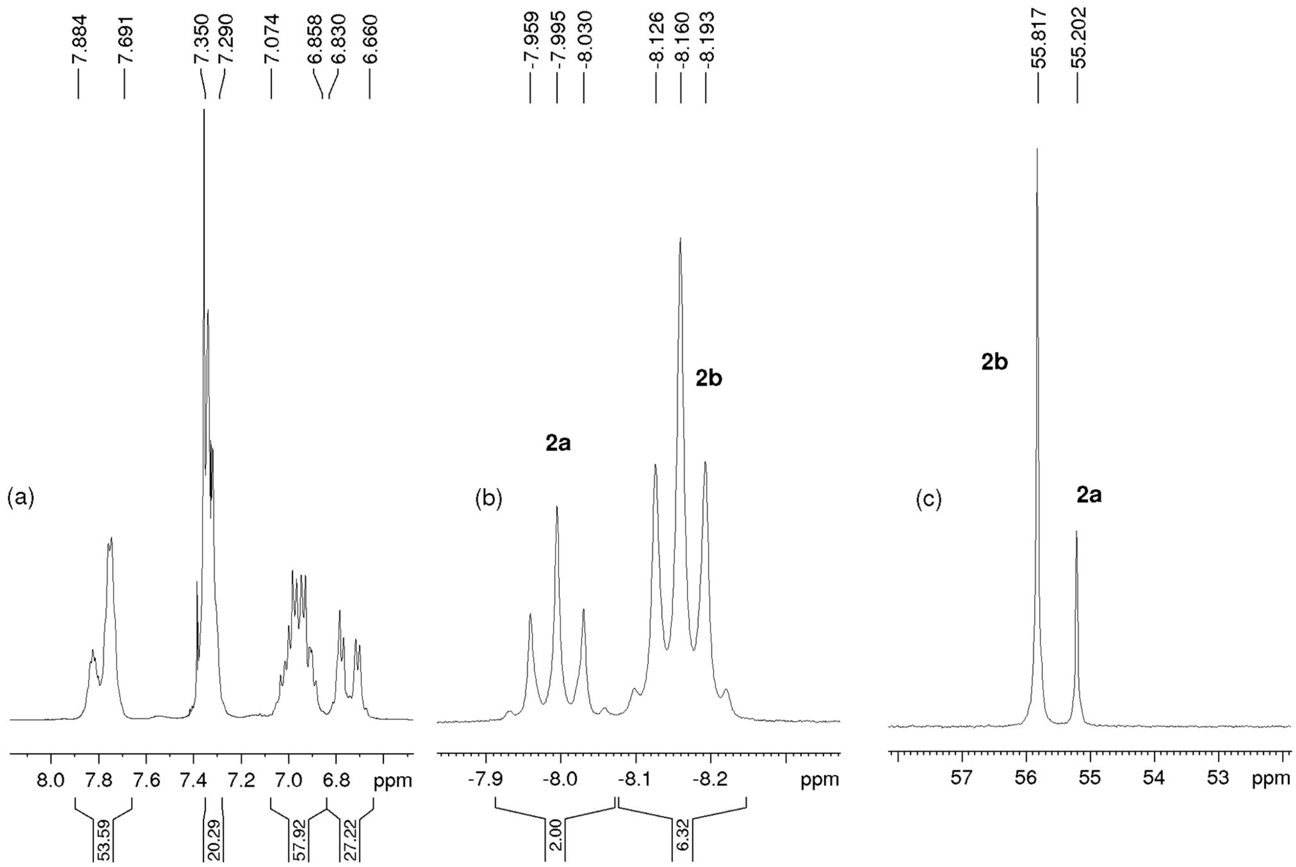

Figure S11. (a) ZnPh and (b) RuH regions of the ^{1}H NMR spectrum (400 MHz, [D8]-THF, 193 K) and (c) $^{31}P\{^{1}H\}$ NMR spectrum (162 MHz, [D8]-THF, 192 K) of **2a** and **2b**.

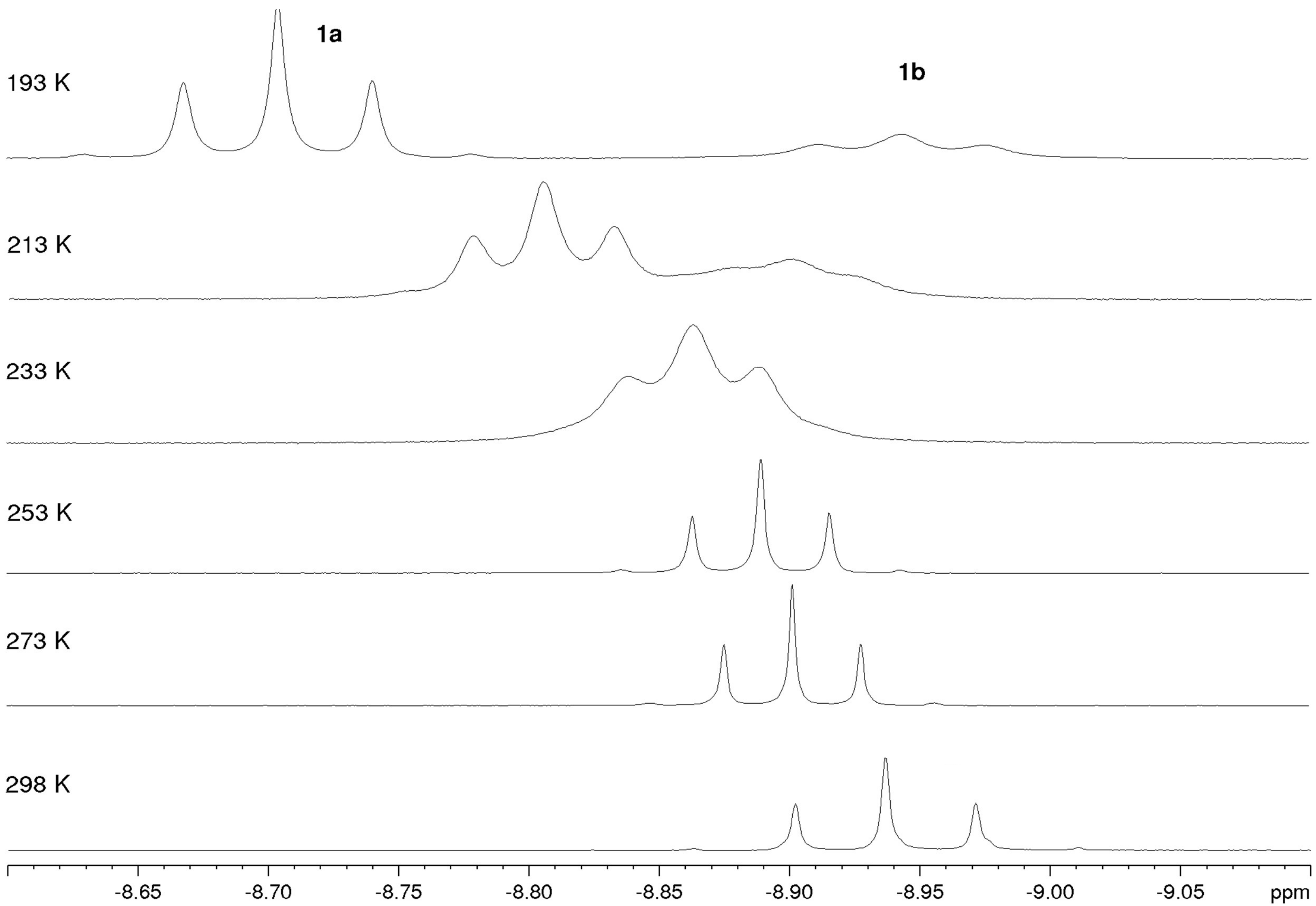

Figure S12. Ru-H region of the variable temperature ^{1}H NMR spectra (400 MHz, [D_8]-THF) of **1a** and **1b**.

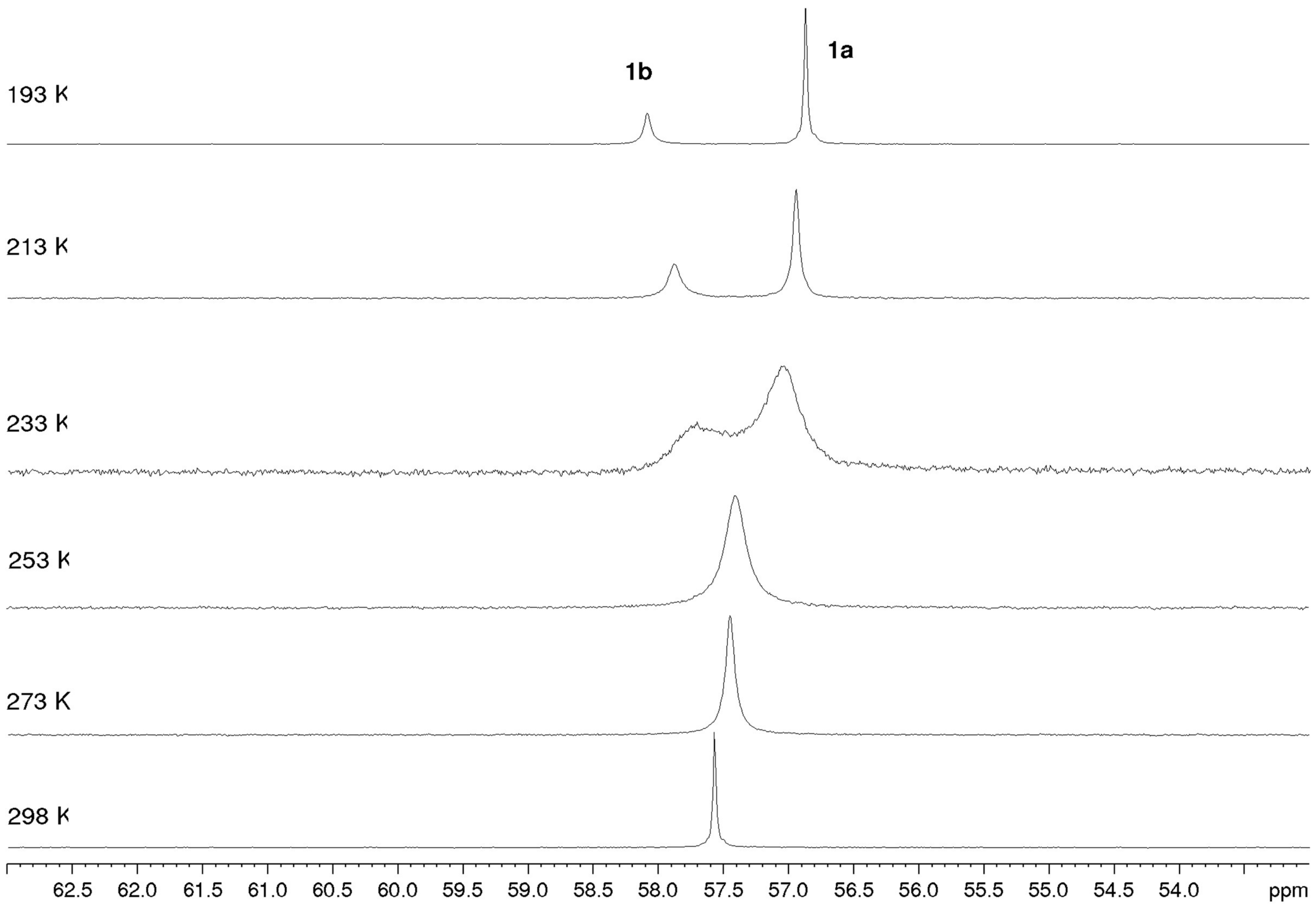

Figure S13. Variable temperature $^{31}P\{^{1}H\}$ NMR spectra (162 MHz, [D$_8$]-THF) of **1a** and **1b**.

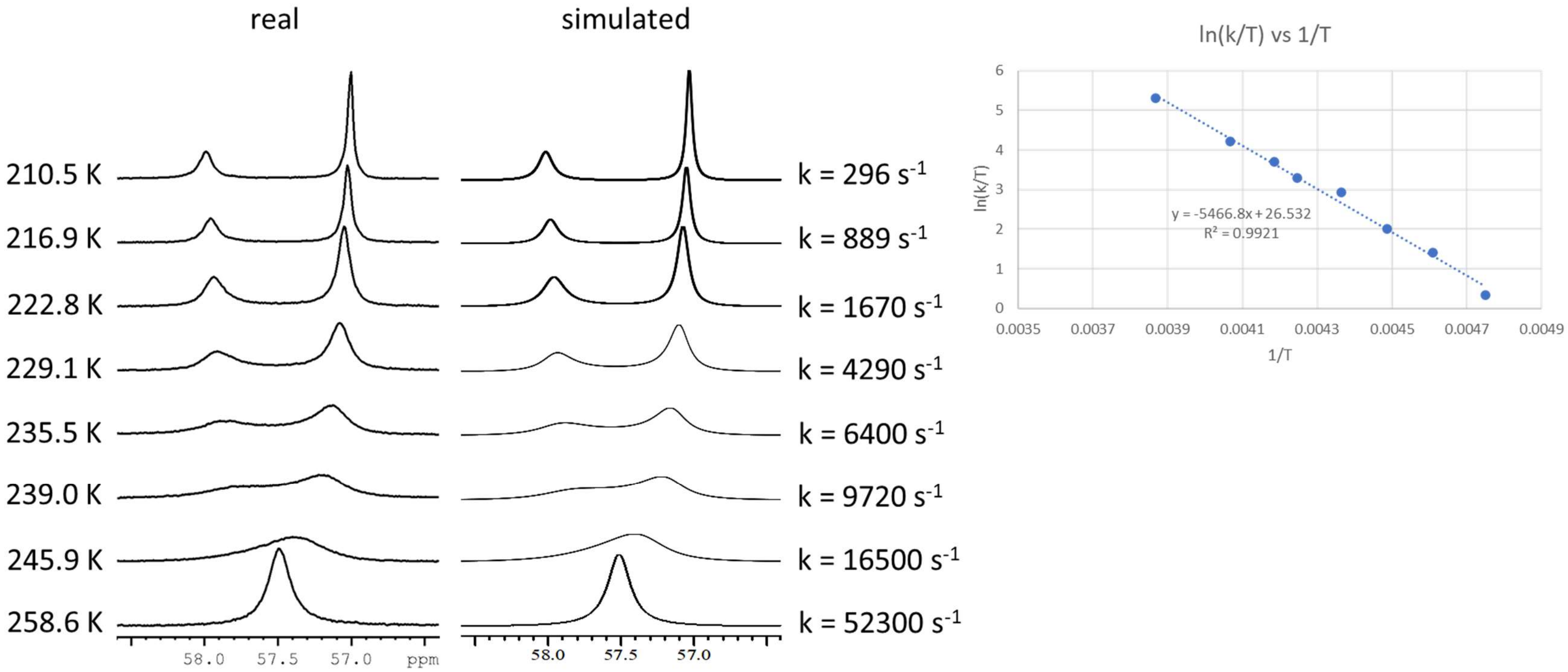

Figure S14. Experimental and simulated variable temperature $^{31}P\{^{1}H\}$ NMR spectra of **1** in [D8]-THF showing the presence of **1a** and **1b** (see Figure S13). Rate constants, obtained from individual fits, are given alongside the corresponding Eyring plot.

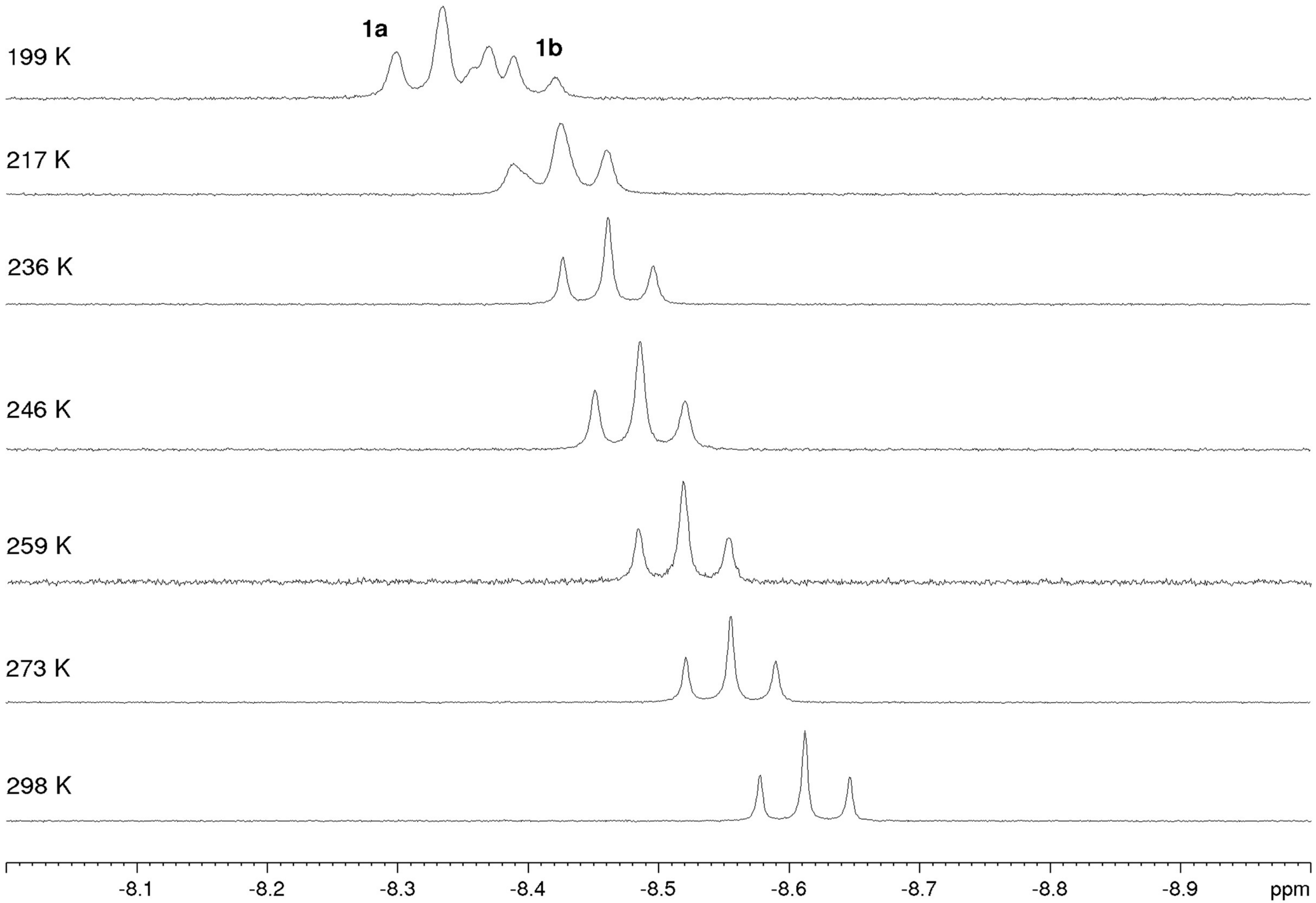

Figure S15. Ru-H region of the variable temperature ^{1}H NMR spectra (400 MHz, [D$_8$]-toluene) of **1a** and **1b**.

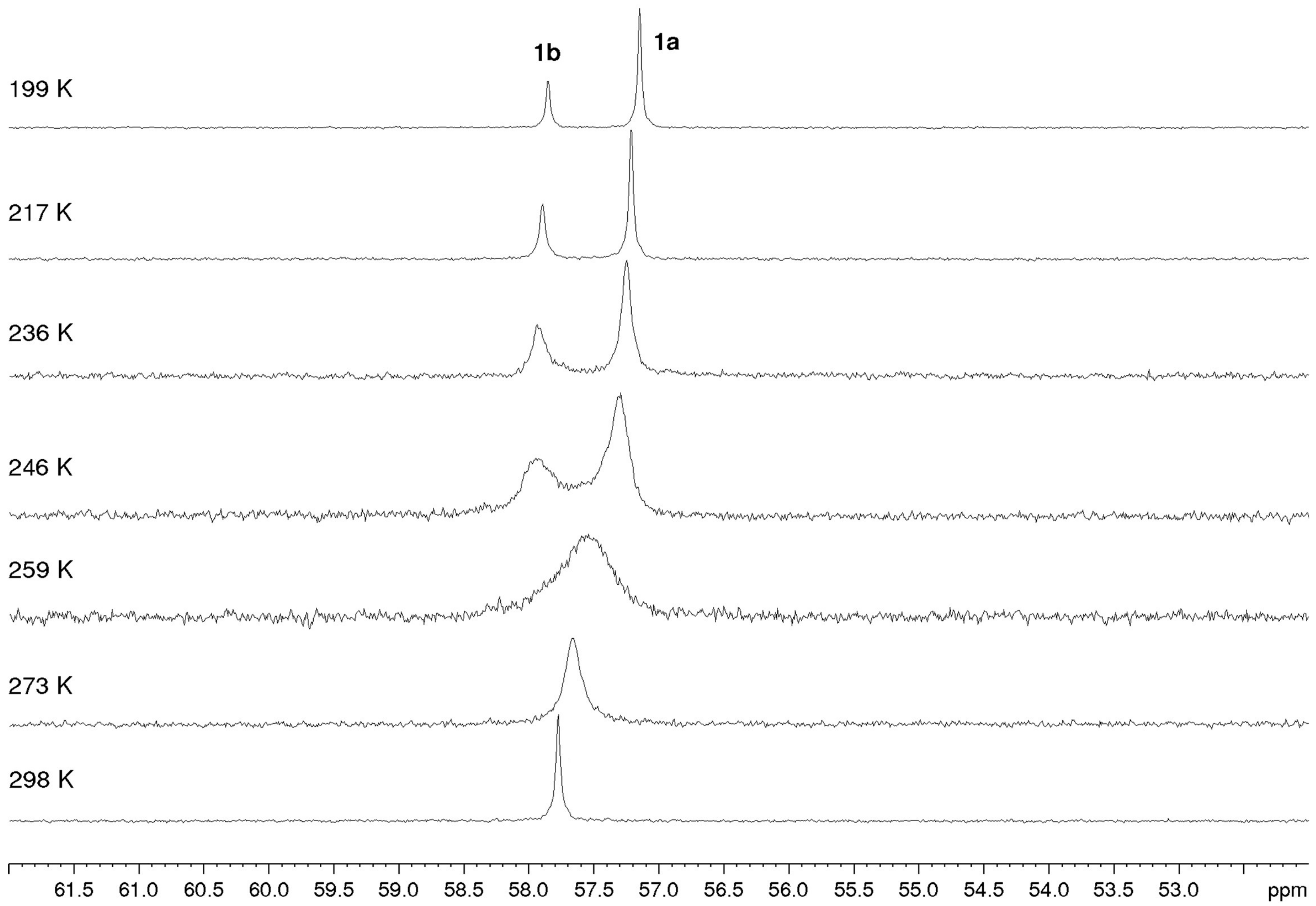

Figure S16. Variable temperature $^{31}P\{^{1}H\}$ NMR spectra (162 MHz, [D_8]-toluene) of **1a** and **1b**.

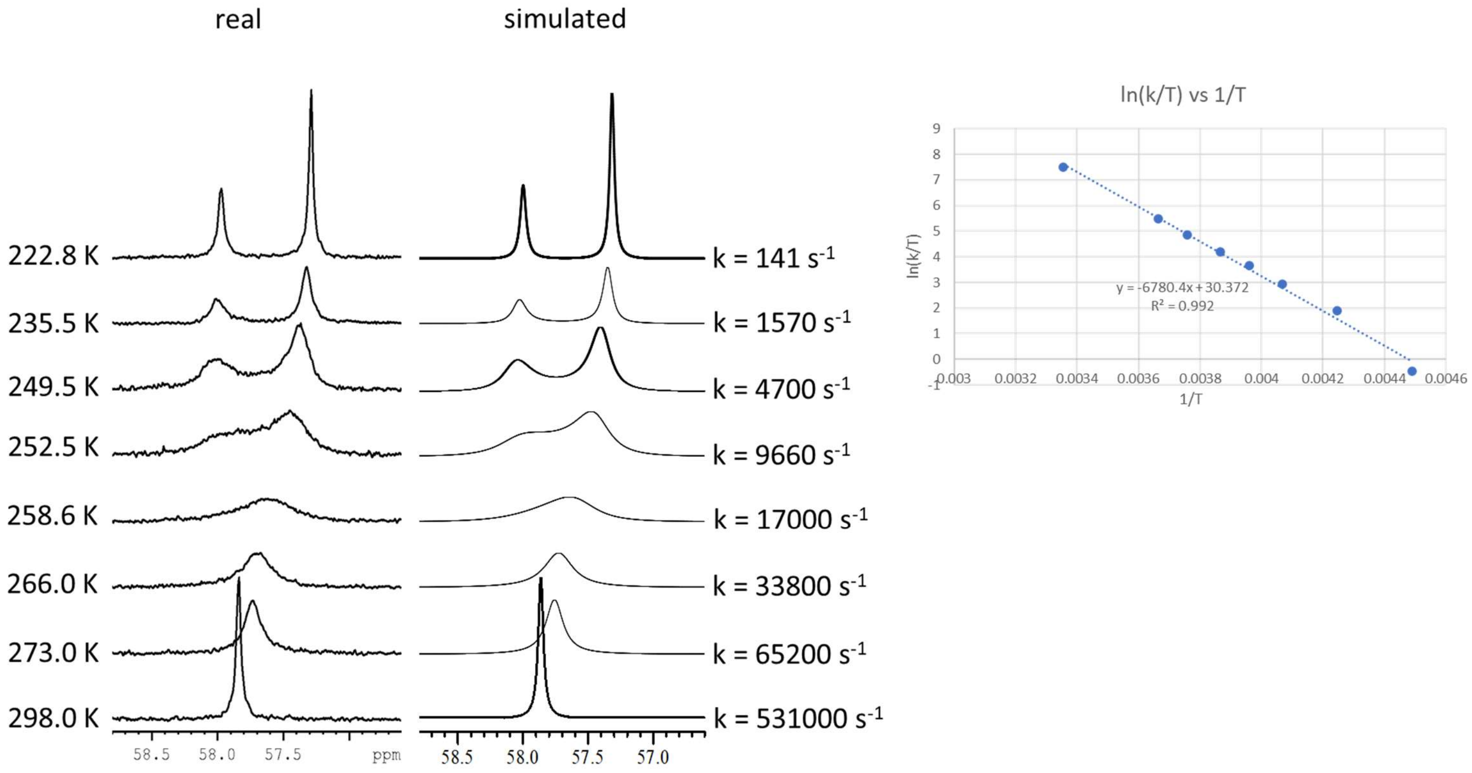

Figure S17. Experimental and simulated variable temperature $^{31}P\{^{1}H\}$ NMR spectra of **1** in [D8]-toluene showing the presence of **1a** and **1b** (see Figure S16). Rate constants obtained from individual fits are given, alongside the corresponding Eyring plot.

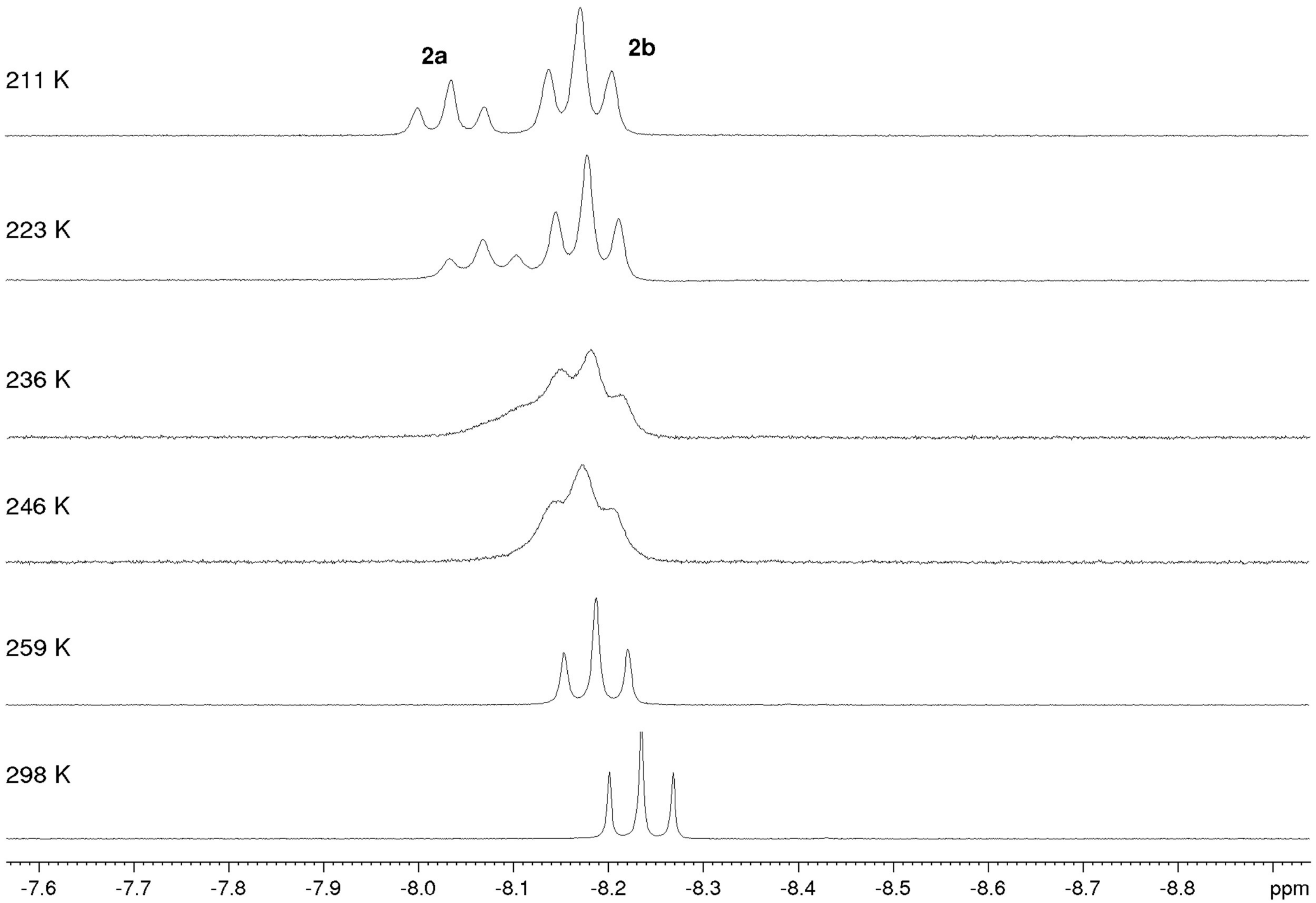

Figure S18. Ru-H region of the variable temperature ^{1}H NMR spectra (400 MHz, [D_8]-THF) of **2a** and **2b**.

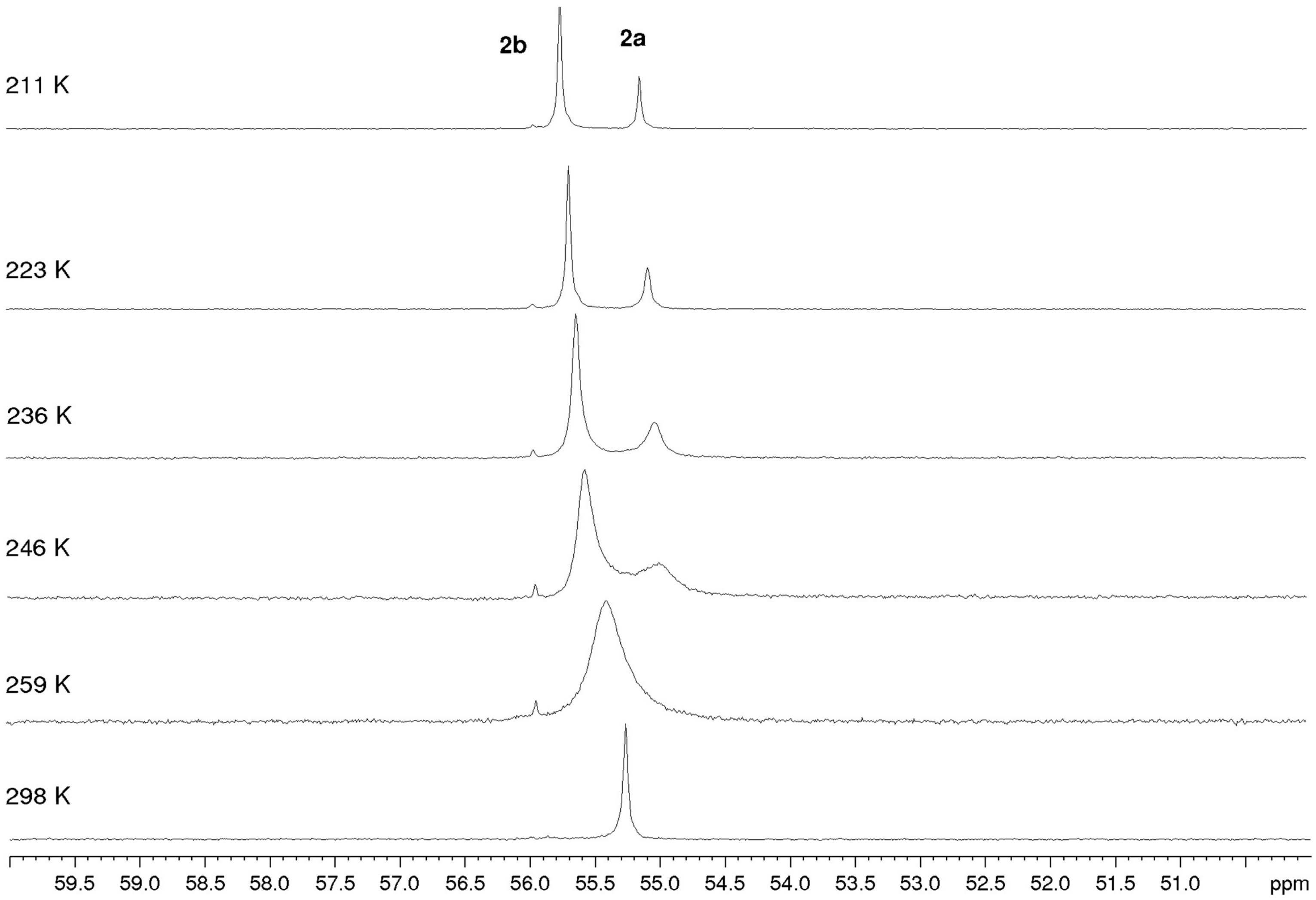

Figure S19. Variable temperature $^{31}P\{^1H\}$ NMR spectra (162 MHz, [D8]-THF) of **2a** and **2b**.

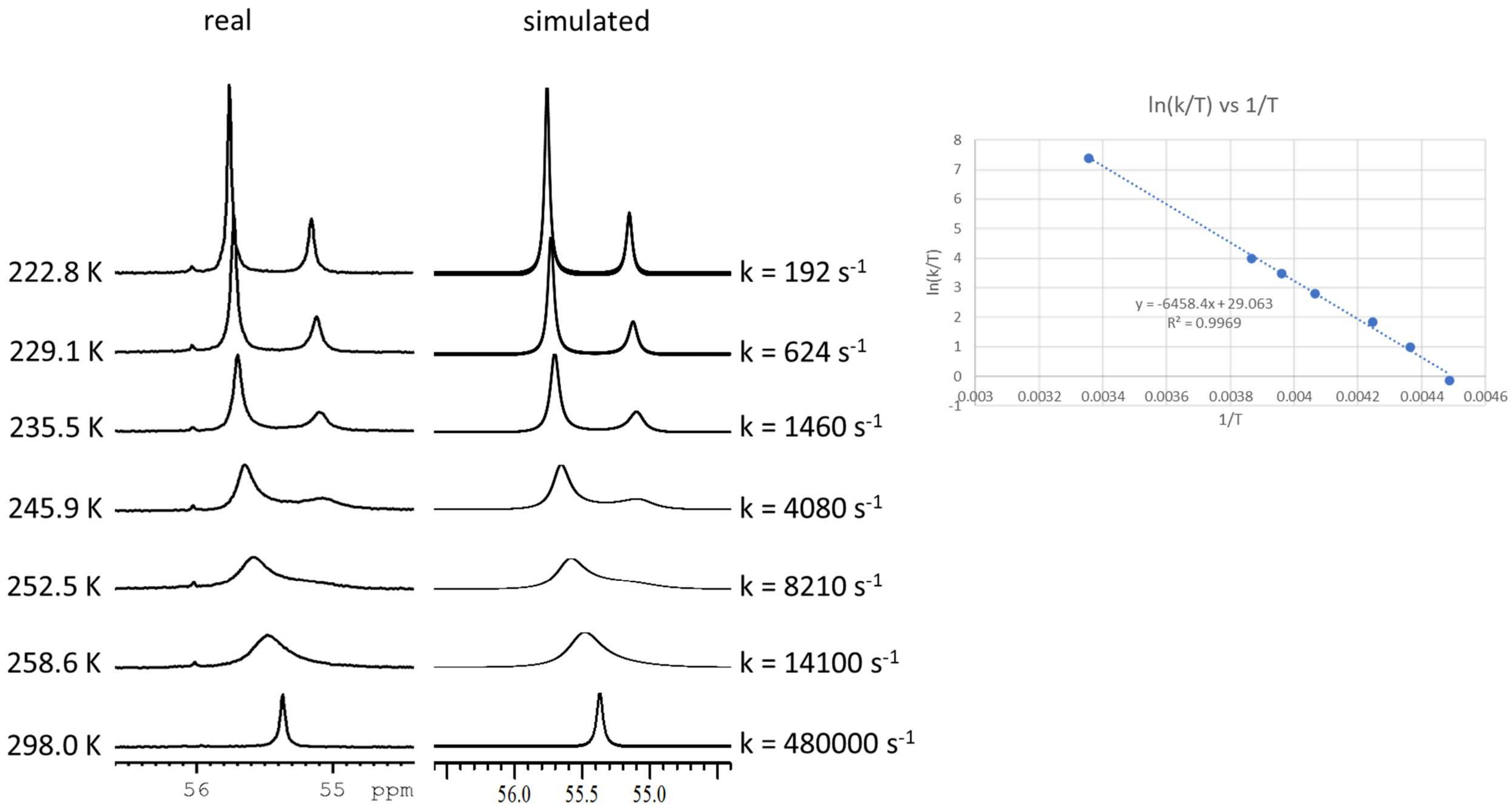

Figure S20. Experimental and simulated variable temperature $^{31}P\{^{1}H\}$ NMR spectra of **2** in [D8]-THF showing the presence of **2a** and **2b** (see Figure S19). Rate constants obtained from individual fits are given, alongside the corresponding Eyring plot.

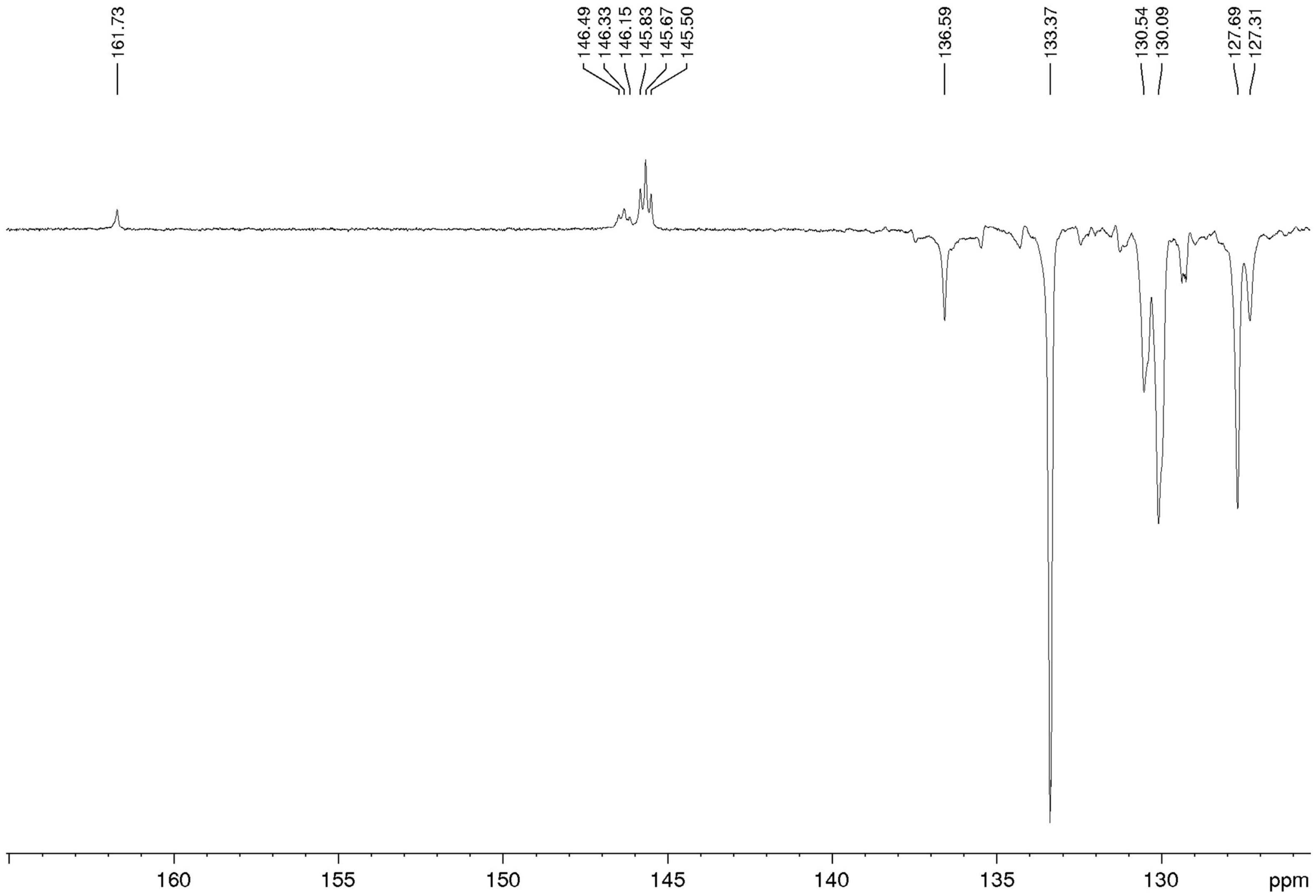

Figure S21. $^{13}C\{^{1}H\}$ DEPTQ NMR spectrum (126 MHz, [D8]-THF, 233 K) showing aromatic resonances arising from **2a** and **2b**

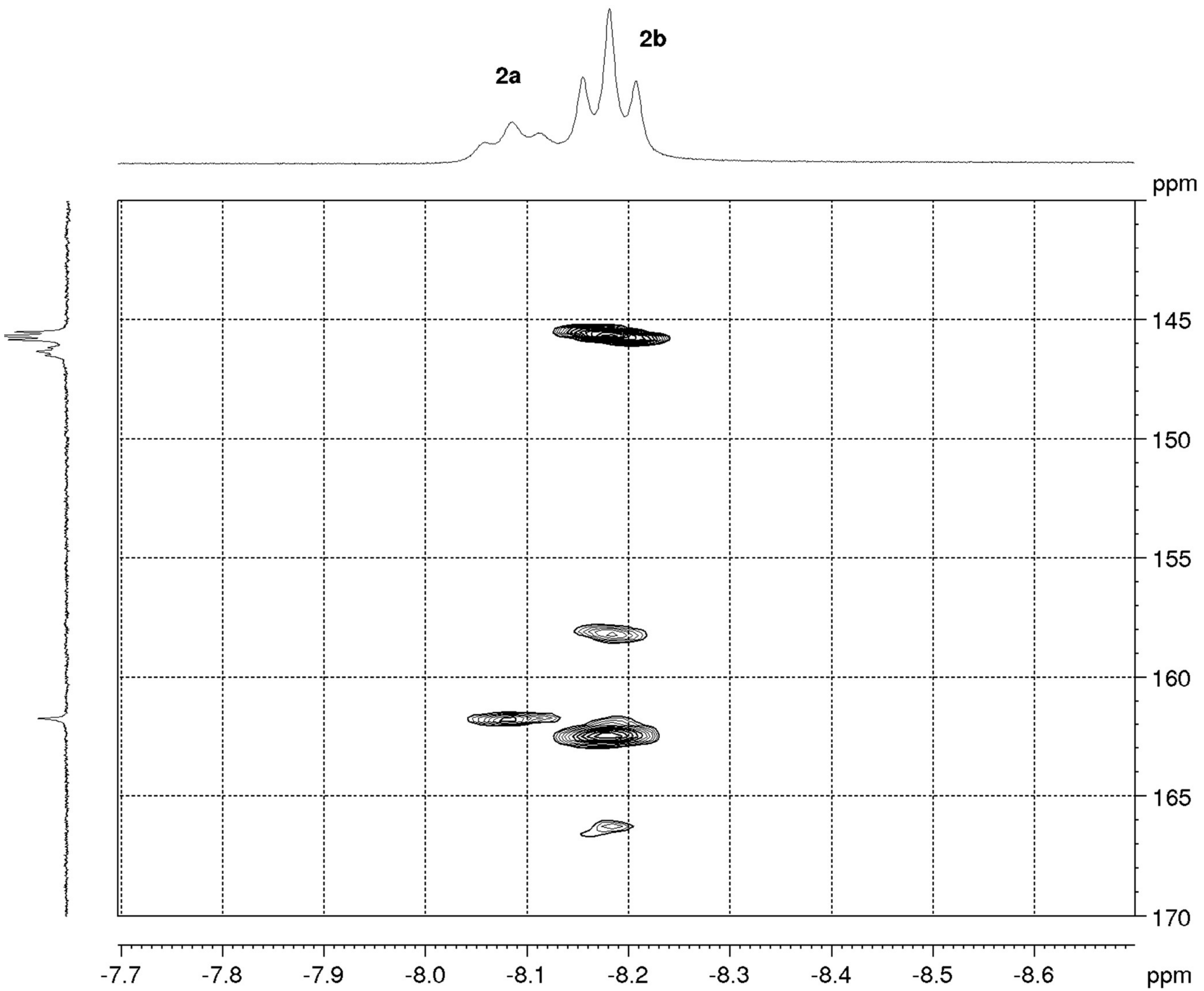

Figure S22. ^{1}H-^{13}C HMBC (500 MHz, [D_8]-THF, 233 K) revealing correlations of Ru-H signals of **2a** and **2b** to ZnPh *ipso*-C resonances

Table S1. Thermodynamic parameters for the isomerisations of **1a**/**1b** and **2a**/**2b** obtained from Eyring plot analysis.

Complex	Solvent	Nuclei	$\Delta H^{\ddagger}$ (kcal mol^{-1})	$\Delta S^{\ddagger}$ (cal K^{-1} mol^{-1})	$\Delta G^{\ddagger}_{298K}$ (kcal mol^{-1})
**1**	THF-d_8	^{31}P	10.9 ± 0.4	5.5 ± 1.7	9.3 ± 0.6
**1**	Tol-d_8	^{31}P	13.5 ± 0.5	13.1 ± 2.2	9.6 ± 0.8
**2**	THF-d_8	^{31}P	12.8 ± 0.4	10.5 ± 1.3	9.7 ± 0.6

S-2 Crystallographic Details

X-ray crystallography. Data for **1a**, **2b** and [Ru(PPh_3)$_2$(ZnMe)$_3${Li(THF)$_2$}H_2] were obtained using an Agilent SuperNova instrument and a Cu-Kα source while those for the P$\bar{1}$ polymorph of **1a** and [Ru(PPh_3)$_2$(ZnMe)$_3${Li(OEt$_2$)}H_2] (**3**) were collected on an Agilent Xcalibur diffractometer (using a Mo-Kα radiation). All experiments were conducted at 150 K with the exception of the data collection for [Ru(PPh_3)$_2$(ZnMe)$_3${Li(THF)$_2$}H_2] which was achieved at 240 K (Table S2). Structures were solved using SHELXT[4] and refined using SHELXL[5] via the Olex2[6] interface. Only points of merit follow.

Half of one molecule equates to the asymmetric unit in **1a** where the central ruthenium is co-incident with a crystallographic inversion centre that serves to generate the remainder of the complex. In **2b**, the asymmetric unit plays host to one molecule of benzene in addition to one molecule of the complex. The asymmetric unit in P$\bar{1}$ polymorph of **1a** was seen to contain half of one molecule. The remainder arises by virtue of a crystallographic inversion centre that is coincident with the ruthenium centre. Hydride ligands were located and refined without restraints in **1a**, **2b**, P$\bar{1}$ polymorph of **1a** and **3**.

Obtaining a final model for the structure of [Ru(PPh_3)$_2$(ZnMe)$_3${Li(THF)$_2$}H_2] was a somewhat tortuous path. An initial data collection suggested either modulation, some twinning of the sample, a phase transition – or some combination of these possibilities. Thus, a second data collection was brokered at a higher temperature (240 K vs 150 K) in the hope of negating the diffraction artefacts. However, it was found that the satellite diffraction peaks still persisted at 240 K (at lower resolutions in particular). Thus, the data were integrated to take account of 1-dimensional modulation (vector = 8.00e-004, 0.2618, 0.0013). Solution and refinement was then effected in Jana where it became quickly evident that refinement involving the satellite data was not converging with any success. What this endeavour did

achieve, however, was to indicate that the disorder in the motif is ‘real’ rather than a function of the potential modulation. The results presented here represent an optimal model based on a typical integration of diffraction data (as a single crystal) – and they are unambiguous in terms of chemical characterisation. The asymmetric unit in $[Ru(PPh_3)_2(ZnMe)_3\{Li(THF)_2\}H_2]$ comprises half of one molecule wherein Ru1, Zn1 C1 and Li1 are coincident with a crystallographic 2-fold rotation axis. 55:35 disorder was modelled for 5 of the phenyl carbons in the rings based on C9 and C15, and the THF ligand was treated for 50:50 disorder. Distance and ADP restraints were employed in disordered regions to assist convergence. While the esds pertaining to the metric data are acceptable, no major claims will be made on the basis of distance and angle data given the features of the raw diffraction data in tandem with the combination of crystallographic and positional disorder. There is clearly additional smearing of the election density in the region of the THF groups which did not lend itself to successful modelling.

Crystallographic data for all compounds have been deposited with the Cambridge Crystallographic Data Centre as supplementary publications CCDC 2119510 (**1a**), 2119509 (**2b**), 211508 ($P\overline{1}$ polymorph of **1a**), 211511 (**3**) and 211512 ($[Ru(PPh_3)_2(ZnMe)_3\{Li(THF)_2\}H_2]$). Copies of these data can be obtained free of charge on application to CCDC, 12 Union Road, Cambridge CB2 1EZ, UK [fax(+44) 1223 336033, e-mail: deposit@ccdc.cam.ac.uk.

Identification code	**1a**	**2b**	**1a** (polymorph)	**3**	$[Ru(PPh_3)_2(ZnMe)_3\{Li(THF)_2\}H_2]$
Empirical formula	$C_{40}H_{44}P_2RuZn_4$	$C_{66}H_{58}P_2RuZn_4$	$C_{40}H_{44}P_2RuZn_4$	$C_{43}H_{51}LiOP_2RuZn_3$	$C_{47}H_{55}LiO_2P_2RuZn_3$
Formula weight	949.24	1275.61	949.24	949.90	1017.97
Temperature/ K	150.01(10)	150.00(10)	150.01(10)	150.00(10)	240.00(10)
Crystal system	monoclinic	triclinic	triclinic	monoclinic	monoclinic
Space group	$P2_1/c$	$P-1$	$P-1$	$P2_1/c$	$C2/c$
a/ Å	11.5032(3)	12.7497(3)	9.3754(4)	12.8703(2)	16.0723(2)
b/ Å	16.8019(3)	15.0545(3)	10.1136(5)	18.7512(2)	13.1885(1)
c/ Å	11.1990(3)	15.7170(3)	11.4001(5)	18.4990(2)	21.5154(2)
α/ °	90	94.169(2)	106.799(4)	90	90
β/ °	116.007(3)	95.178(2)	109.907(4)	108.503(1)	90.501(1)
γ/ °	90	110.978(2)	94.877(4)	90	90
U/ Å^3	1945.32(9)	2787.33(11)	952.57(8)	4233.65(9)	4560.44(8)
Z	2	2	1	4	4
ρ_{calc}/ g cm^{-3}	1.621	1.520	1.655	1.490	1.483
μ/ mm^{-1}	2.919	4.907	2.980	2.135	5.351
F(000)	956.0	1296.0	478.0	1936.0	2080.0
Crystal size/ mm^3	0.261 × 0.206 × 0.148	0.155 × 0.106 × 0.086	0.699 × 0.218 × 0.192	0.369 × 0.315 × 0.131	0.268 × 0.266 × 0.222
2θ range for data collection/°	6.248 to 58.67	5.682 to 147.07	6.784 to 60.494	6.676 to 60.312	8.22 to 146.37
Index ranges	$-15 \le h \le 14$, $-22 \le k \le 22$, $-13 \le l \le 14$	$-15 \le h \le 15$, $-18 \le k \le 17$, $-19 \le l \le 18$	$-10 \le h \le 12$, $-11 \le k \le 14$, $-16 \le l \le 12$	$-16 \le h \le 18$, $-24 \le k \le 26$, $-25 \le l \le 24$	$-19 \le h \le 16$, $-16 \le k \le 15$, $-26 \le l \le 26$
Reflections collected	15775	35609	8755	47231	28735
Independent reflections, R_{int}	4613, 0.0240	11147, 0.0234	4787, 0.0154	11178, 0.0217	4546, 0.0309
Data/restraints/parameters	4613/0/220	11147/0/666	4787/0/220	11178/0/473	4546/74/380
Goodness–of–fit on F^2	1.051	1.075	1.044	1.029	1.033
Final $R1$, $wR2$ [I>=2σ(I)]	0.0241, 0.0575	0.0319, 0.0854	0.0215, 0.0489	0.0223, 0.0495	0.0442, 0.1102
Final $R1$, $wR2$ [all data]	0.0284, 0.0597	0.0323, 0.0858	0.0259, 0.0512	0.0275, 0.0515	0.0456, 0.1115
Largest diff. peak/hole/ e Å^{-3}	0.41/−0.69	0.71/−0.83	0.37/−0.37	0.40/−0.40	0.56/−1.39

Table S2. Crystallographic details for 1a, 2b, P $\overline{1}$ polymorph of **1a**, **3** and $[Ru(PPh_3)_2(ZnMe)_3\{Li(THF)_2\}H_2]$.

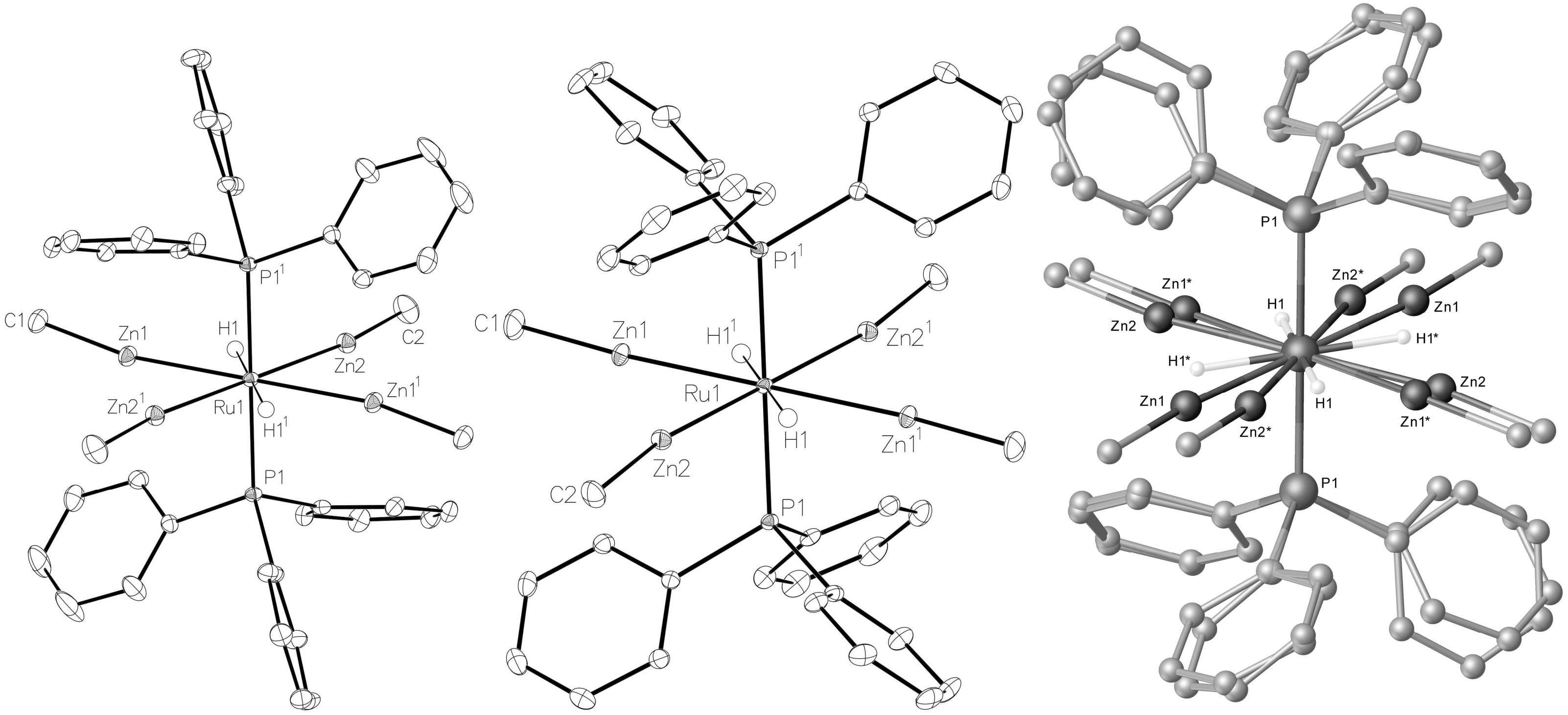

Figure S23. X-ray crystal structures of (top left) **1a** (labels superscripted with '1' are related to those in the asymmetric unit by the $1 - x$, $1 - y$, $1 - z$ symmetry operation) and (top right) P$\bar{1}$ polymorph of **1a** (labels superscripted with '1' are related to those in the asymmetric unit by the $1 - x$, $1 - y$, $2 - z$ symmetry operation). Overlay of the two structures (bottom), with asterisked labels for the P$\bar{1}$ polymorph. Ellipsoids are represented at 30% probability. Hydrogen atoms, with the exception of Ru-H, have also been omitted for clarity.

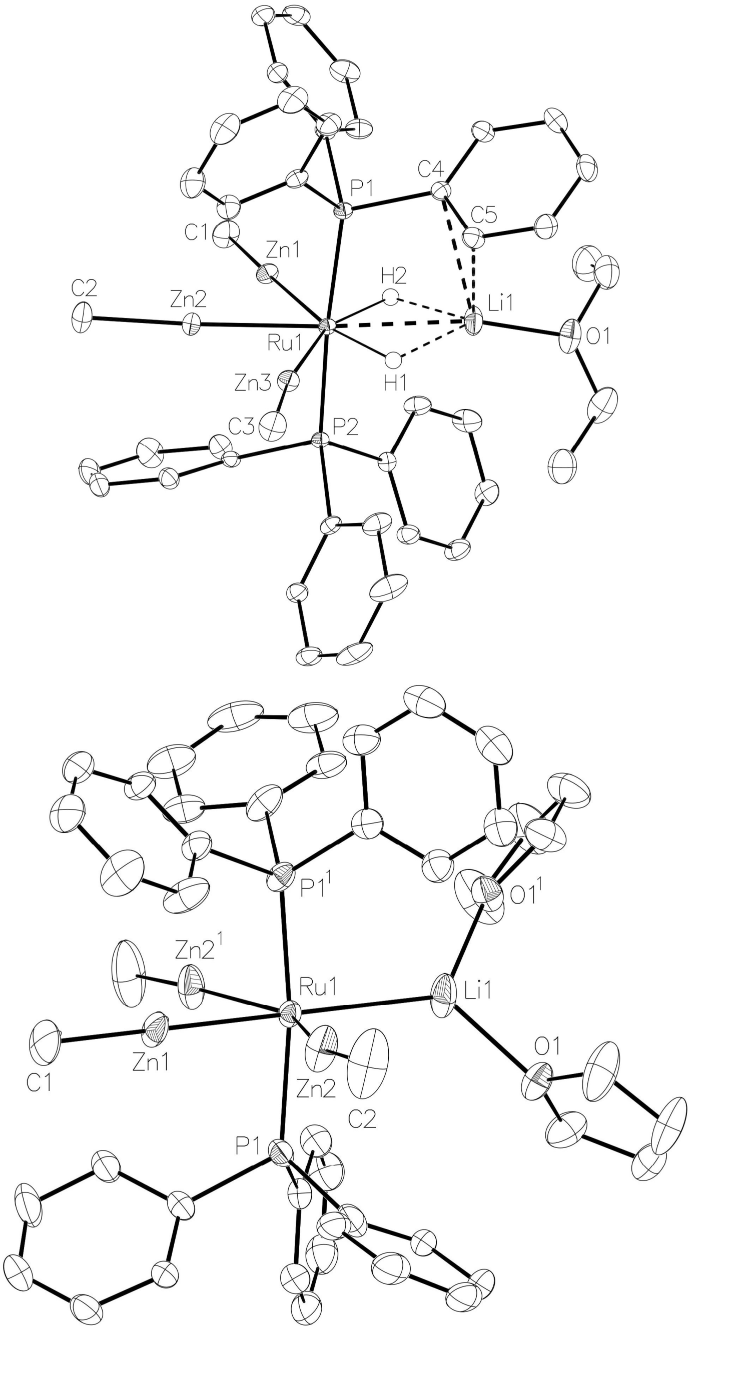

Figure S24. (Top) X-ray crystal structure of $[Ru(PPh_3)_2(ZnMe)_3\{Li(OEt_2)\}H_2]$ (**3**). Ellipsoids are represented at 30% probability with solvent omitted from the plot for clarity. (Bottom) X-ray crystal structure of $[Ru(PPh_3)_2(ZnMe)_3\{Li(THF)_2\}H_2]$. Ellipsoids are represented at 25% probability. Only one component of disorder shown and labels superscripted with '1' are related to those in the asymmetric unit by the $1 - x$, y, $½ - z$ symmetry operation.

S-3 Computational Studies

Computational Details. DFT calculations were run with Gaussian 16 (Revision C.01).[7] Ru, Zn and P centres were described with Stuttgart RECPs and associated basis sets[8] with 6-31G** basis sets used for all other atoms.[9,10] A set of d-orbital polarisation functions was also added to P (ζ^{d}=0.387).[11] Optimisations employed the BP86[12,13] functional and all stationary points were fully characterised via analytical frequency calculations as either minima (all positive eigenvalues) or transition states (one negative eigenvalue). Transition states were also characterised via IRC calculations and subsequent geometry optimisations confirmed they linked to the minima as reported in the text or in the Supporting Information. The electronic energies were then recomputed with the ωB97X-D functional[14] and the basis set def2TZVP[15] (that has previous proved effective in related studies)[3] and corrected for the effects of toluene solvent using the PCM approach.[16] The corrected electronic energies were then combined with the thermochemical corrections from the BP86-optimised geometries to give the free energies quoted in the text.

Electronic structure analyses were performed on structures using the heavy atom positions derived from the crystallographic studies with H atom positions optimised with the BP86 functional. Quantum theory of atoms in molecules (QTAIM)[17] used the AIMALL program.[18] NCI calculations were based on the promolecular densities and used NCIPLOT[19] with visualisation via VMD.[20] Natural orbitals for chemical valence (NOCV) analyses[21] were run using the Amsterdam Modeling Suite (AMS) package.[22] Conformational searches were carried out on Tinker Molecular Modeling[23] using the MM3[24] force field using our previous published protocol.[25] Computed geometries are displayed with ChemCraft[26] with all geometries supplied as a separate XYZ file.

S-2.1 Isomers of 1 and 2

For the two isomers of **1** and **2**, a functional screening was carried out on the BP86 optimised structures using a range of functionals, including GGA functionals (PBE,[27] BP86,[12,13] BLYP,[28] B97D,[29] TPSS[30]), hybrid GGA functionals (PBE0,[31] B3LYP,[32] ωB97X-D[14]) and a Minnesota functional (M06).[33] The relative energy between the isomers for each functional is displayed in Table S3 where the geometries are either based directly on the experimental structures of **1a** and **2b** or adapted from those. An alternative 4:0 isomer of **1** where the two hydrides are *cis* to each other, **1c**, was also computed but was found to be higher in energy with all of the tested functionals.

Table S3. Results of the functional screening conducted on the isomers of **1** and **2**. Energies in kcal/mol.

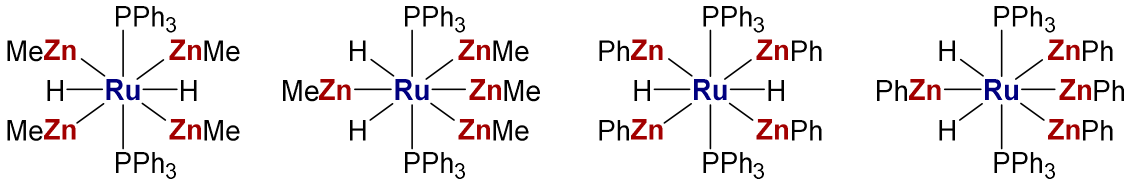

1a **1b** **2a** **2b**

Relative energy	BP86	BLYP	B3LYP	PBE	PBE0	B97D3	M06	TPSS	ωB97XD
**1** (**1b**-**1a**)	-2.5	-2.7	-2.2	-2.7	-2.1	-2.8	-0.5	-1.9	-2.3
**2** (**2b**-**2a**)	-1.7	-2.1	-1.6	-2.1	-1.4	-2.2	-1.2	-0.9	-1.7

In the case of **2**, the relative energy is in agreement with the experimentally observed ratio (ratio **2b**:**2a** = 3.2:1 at 193 K in [D_8]-THF), while in the case of **1**, a small yet positive value would be expected (ratio **1a**:**1b** = 2.7:1 at 193 K in [D_8]-THF). In light of isomer **1b** being predicted to be slightly more stable than **1a** a conformational study was conducted using Tinker in both isomers of **1** and **2** and the results are shown in Table S4. While these still correctly predict **2b** to be somewhat more stable than **2a**, with the exception of the M06 functional, **1b** remains more stable than **1a**.

Table S4. Results of the functional screening conducted on the isomers of **1** and **2** after performing a conformational study. Energies in kcal/mol.

Relative energy	BP86	BLYP	B3LYP	PBE	PBE0	B97D3	M06	TPSS	ωB97XD
**1** (**1b**-**1a**)	-1.9	-2.1	-1.5	-2.1	-1.4	-2.1	0.4	-1.3	-1.5
**2** (**2b**-**2a**)	-1.7	-2.1	-1.6	-2.1	-1.5	-2.3	-1.2	-0.9	-1.7

In Table S5 the free energies and enthalpies of activation of the **1a↔1b** and **1a↔1b** isomerisations are displayed. In order to compare the results with the experimental data, the corresponding energies using THF as solvent were also computed.

Table S5. Thermodynamic parameters for the isomerisations of **1a**/**1b** and **2a**/**2b** obtained in the ωB97x-D(PCM=solvent)/def2-TZVP//BP86/6-31G**,SDD(Ru,Zn,P) theory level.

Complex	Solvent	$\Delta G^{\ddagger}$ (kcal/mol)	$\Delta H^{\ddagger}$ (kcal/mol)
**1**	THF	14.7	13.6
**1**	Toluene	16.2	15.1
**2**	THF	16.9	16.2
**2**	Toluene	16.3	15.6

S-2.2 ZnMe/ZnPh exchange reactions

The alternative reaction profile for the first ZnMe/ZnPh exchange where $ZnPh_2$ approaches between two ZnMe ligands is shown in Figure S25.

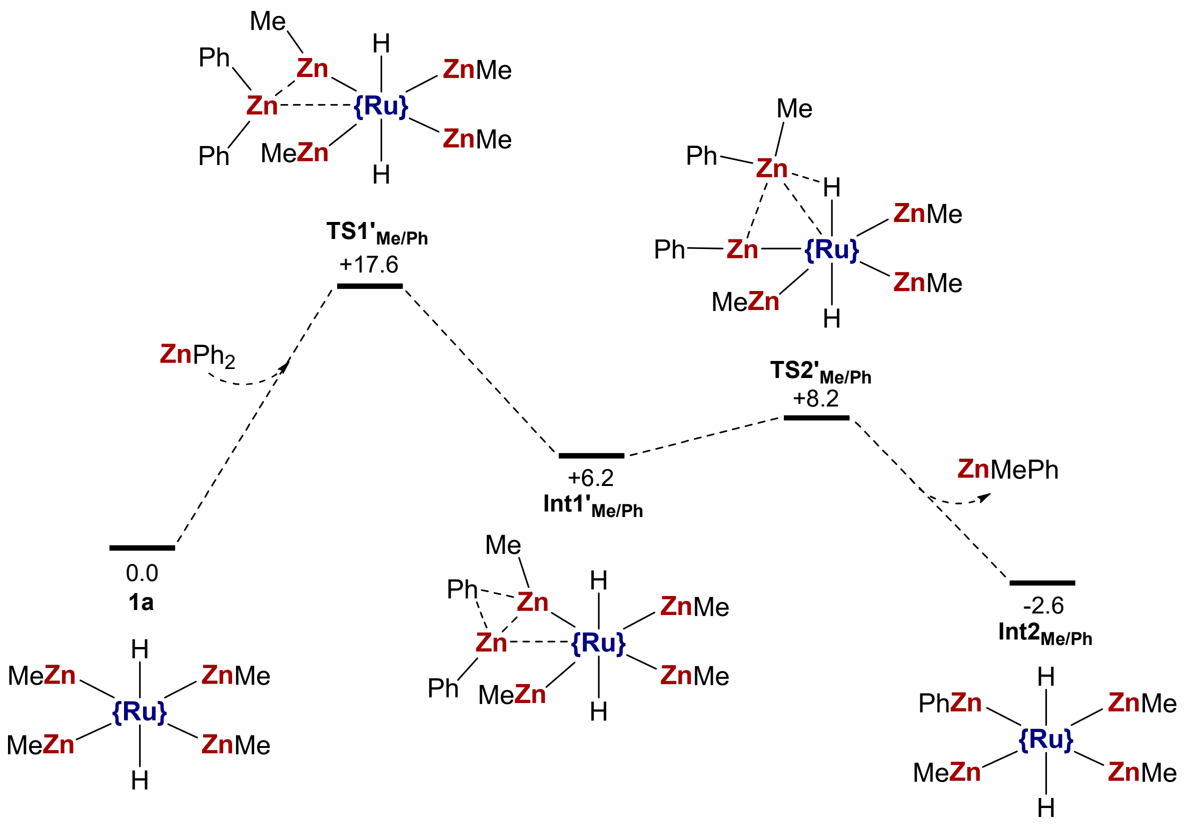

Figure S25. Reaction mechanism of the alternative phenyl exchange in **1a**. {Ru} = [Ru(PPh_3)$_2$]. Energies in kcal/mol.

The thermodynamics for the subsequent ZnMe/ZnPh exchanges are shown in Figure S26. For the third and fourth exchanges ZnMePh was taken as the source of the Ph group, as only 2.5 equiv of $ZnPh_2$ was employed experimentally. $ZnMe_2$ is therefore expelled in these processes.

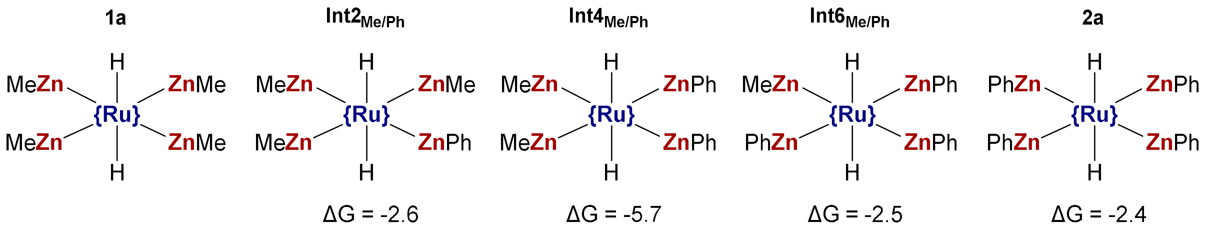

Figure S26. Computed free energy changes for each ZnMe/ZnPh exchange (**Int2$_{Me/Ph}$** and **Int4$_{Me/Ph}$** using $ZnPh_2$ and **Int6$_{Me/Ph}$** and **2a** using ZnMePh). Energies in kcal/mol.

DFT calculations indicate that the driving force of this reaction is the dispersive stabilization that occurs after each of these ZnMe/ZnPh exchanges, as shown in Table S6. The data in columns 1-5 are computed with the BP86 functional as this allows a D3BJ dispersion correction to be added in separately and allows this effect to be quantified. The final free energy computed with the BP86 functional (with all corrections) is similar to that reported in the main text based on the ωB97X-D//Def2TZVPP energies (column 6).

	Electronic energy	Free energy (BP86)	Solvent corrections	**Dispersion corrections**	Final free energy (BP86)	Free energy (ωB97X-D)
**Int2Me/Ph**	-0.6	-0.9	-0.4	**-3.5**	-3.4	-2.6
**Int4Me/Ph**	-1.0	-1.5	-1.2	**-6.1**	-6.4	-5.7
**Int6Me/Ph**	-0.6	-0.2	-0.4	**-4.1**	-3.3	-2.5
**2a**	-1.3	-1.4	-0.4	**-5.2**	-4.1	-2.4

Table S6. Effect of dispersion in the free energies of ZnMe/ZnPh exchanges from **1a** to **2a**. Energies in kcal/mol relative to previous intermediate, starting on **1a**.

The computed reaction profile for the last exchange in shown in Figure S27.

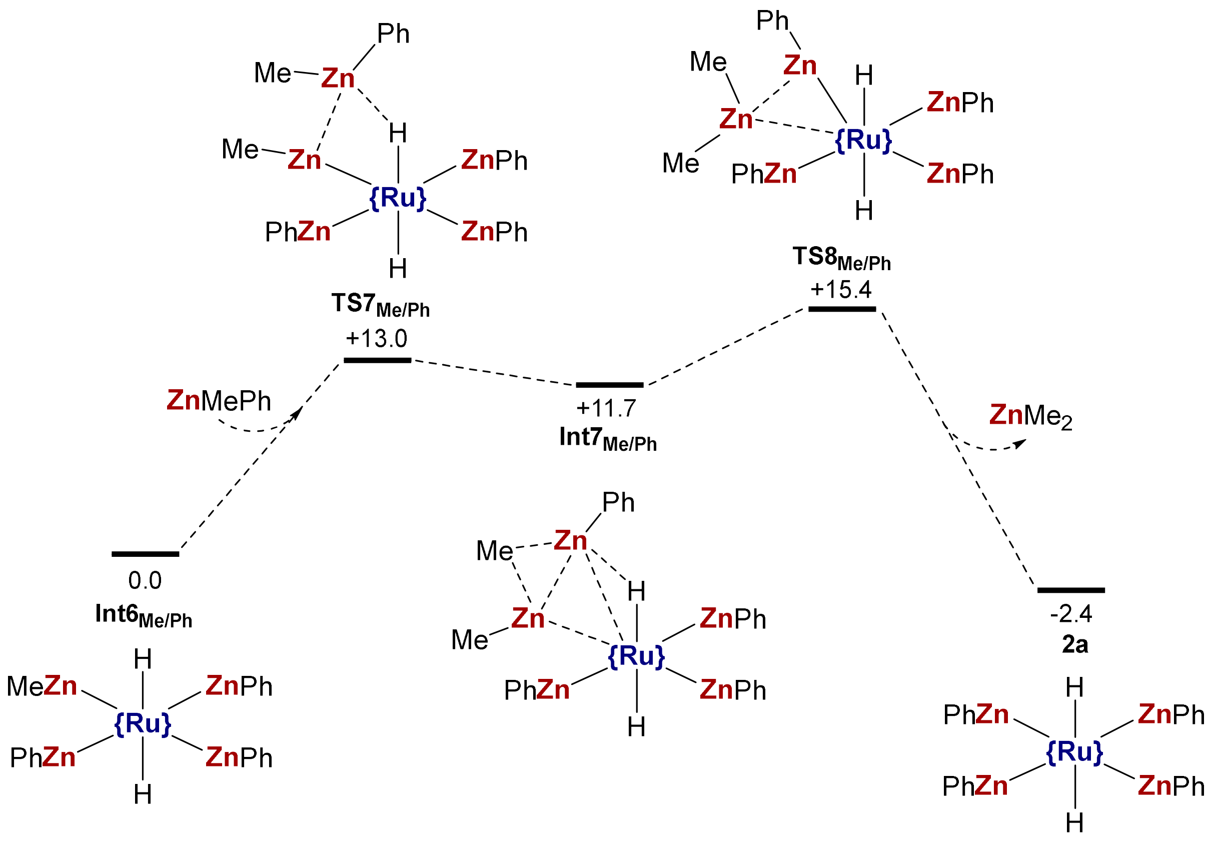

Figure S27. Reaction mechanism of the last phenyl exchange to obtain **2a**. {Ru} = [Ru(PPh_3)$_2$]. Energies in kcal/mol.

S3-2 Electronic Structure Analyses

Complex **1a** was fully optimised using the BP86 functional, Stuttgart RECPs and associated basis sets for Ru, Zn and P centres and 6-31G** basis set for all other atoms, with a set of d-orbital polarisation functions on P (ζ^d=0.387). The resulting structures were in good agreement with the crystallographic data, but the electronic structure analyses were performed fixing all heavy atoms (all but H) for the sake of consistency with our previous work.

Computed QTAIM Metrics

Structures were optimised using the heavy atom positions derived from the crystallographic studies with H atom positions optimised. Relevant bond critical point data associated with the computed complexes are provided in atomic units. Selected parameters are defined as: ρ(r) electron density, $\nabla^2\rho$(r) Laplacian of electron density, ε ellipticity, H(r) local energy density, δ(A,B) delocalisation index.

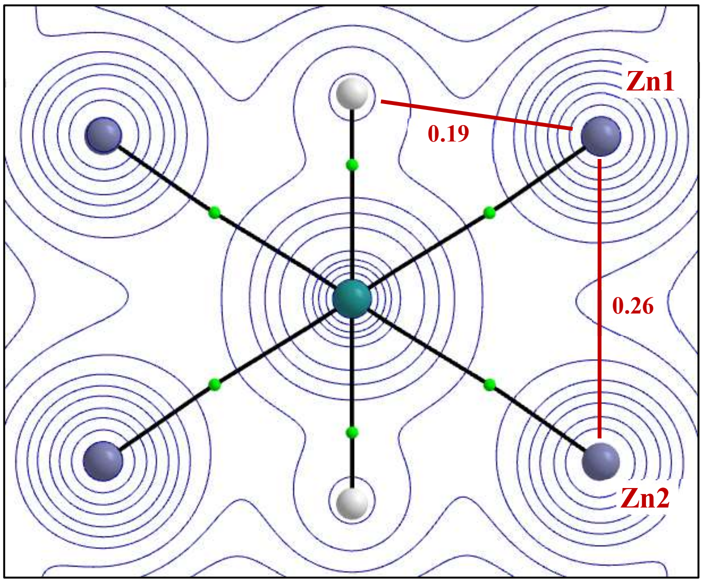

Bond	ρ(r)	$\nabla^2\rho$(r)	ε	H (r)	δ (A,B)
**Ru–H$_A$**	0.105	0.189	0.060	-0.041	0.700
**Ru–Zn1**	0.064	0.083	0.147	-0.021	0.625
**Ru–Zn2**	0.063	0.079	0.170	-0.021	0.621

Figure S28. Molecular graph and associated bond critical point data for complex **1a**. Selected delocalisation indices between atoms not linked by a bond path are shown in red.

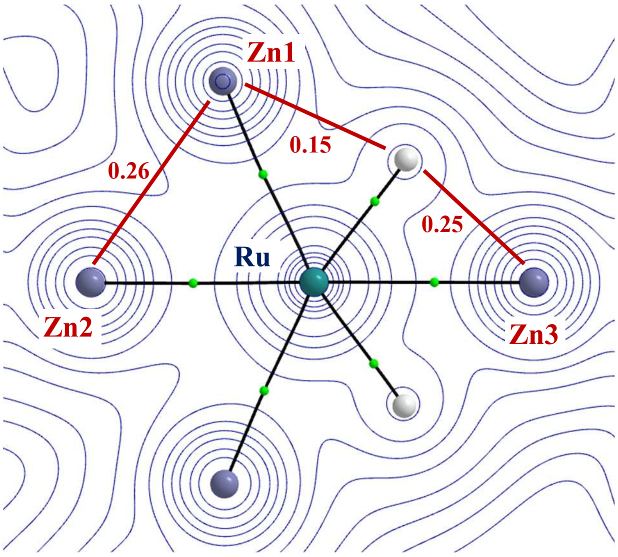

Bond	ρ(r)	$\nabla^2\rho$(r)	ε	H (r)	δ (A,B)
**Ru–H**	0.107	0.178	0.088	0.043	0.665
**Ru–Zn1**	0.065	0.079	0.079	0.022	0.677
**Ru–Zn2**	0.062	0.075	0.229	0.020	0.676
**Ru–Zn3**	0.066	0.094	0.299	0.022	0.518

Figure S29. Molecular graph and associated bond critical point data for complex **2b**. Selected delocalisation indices between atoms not linked by a bond path are shown in red.

ETS-NOCV analyses

The ETS-NOCV method was employed to assess the interaction between a $\{ZnMe\}^+$ fragment and the remaining Ru-based fragment within complexes **1a** and **2b**. The results of energy decomposition analysis are displayed in Table S7. The major deformation density channel of each structure is shown in Figures S30-33, along with their interaction energy, ΔE_{Orb} and charge transfer, Δq. An isovalue of 0.0025 for chosen for the contour plots so that they could be compared to our previous work. Electron flow is shown from red to blue (all H atoms except the ruthenium hydrides were omitted for clarity).

	**1a**	**2b**		
	**{ZnMe}**	**{Zn1Ph}**	**{Zn2Ph}**	**{Zn3Ph}**
ΔE_{Pauli}	139.62	148.62	176.59	140.05
ΔV_{elec}	-195.11	-196.13	-211.45	-187.54
ΔE_{oi}	-131.69	-136.30	-143.98	-125.73
ΔE_{tot}	-187.20	-183.82	-178.83	-173.23

Table S7. Energy decomposition analysis of the fragmentation of **1a** and **2b**. Energies in kcal/mol correspond to the Pauli repulsion, ΔE_{Pauli}, electrostatic interaction, ΔV_{elec}, orbital interaction, ΔE_{oi}, and total bonding energy, ΔE_{tot}.

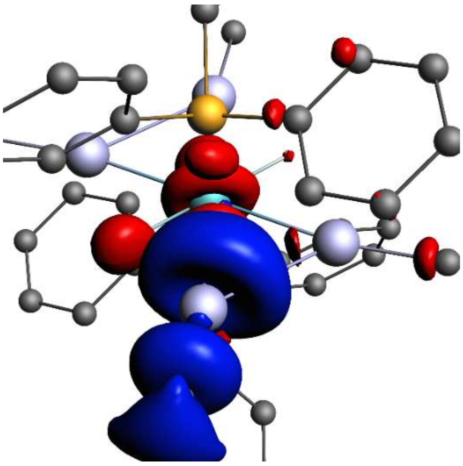

Main NOCV pair

ΔE_{Orb} = -78.0 kcal/mol

Δq = -0.92

Figure S30. Main ETS-NOCV deformation density channel plot of **1a** and associative metrics.

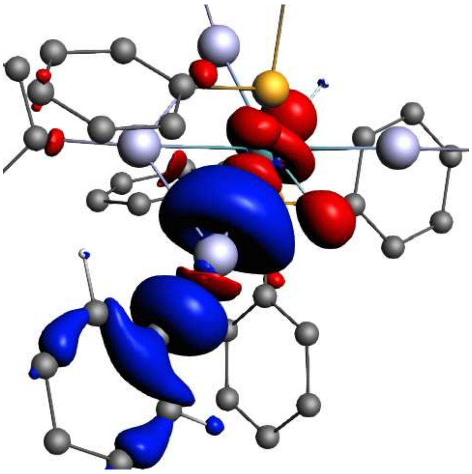

Total ΔE_{Orb} = -136.9 kcal/mol

Main NOCV pair ΔE_{Orb} = -80.6 kcal/mol

Δq = -0.97

Figure S31. Main ETS-NOCV deformation density channel plot of the fragmentation of {Zn1Me} on **2b** and associative metrics.

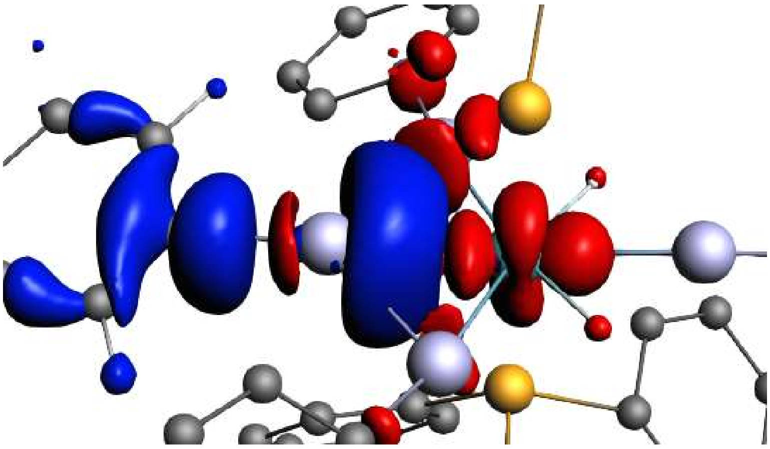

Total ΔE_{Orb} = -144.6 kcal/mol

Main NOCV pair ΔE_{Orb} = -79.8 kcal/mol

Δq = -0.96

Figure S32. Main ETS-NOCV deformation density channel plot of the fragmentation of {Zn2Me} on **2b** and associative metrics.

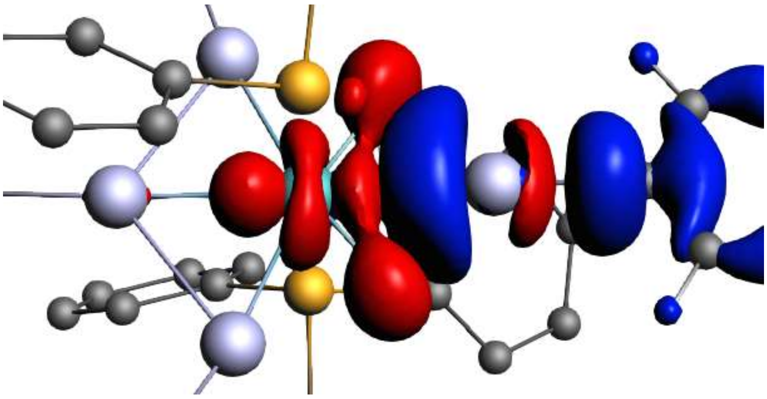

Total ΔE_{Orb} = -126.1 kcal/mol

Main NOCV pair ΔE_{Orb} = -65.1 kcal/mol

Δq = -0.88

Figure S33. Main ETS-NOCV deformation density channel plot of the fragmentation of {Zn2Me} on **2b** and associative metrics.

NCI plots

NCI calculations were performed based on the promolecular densities and used NCIPLOT with visualisation via VMD. The resulting figures for **1a** and **2b** are displayed in Figures S34 and S35.

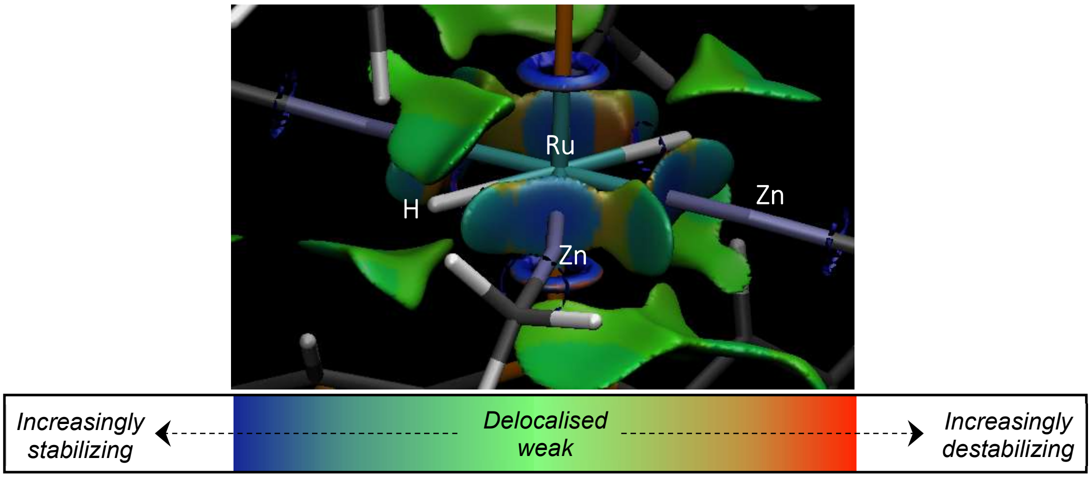

Figure S34. NCI plot of **1a** centred on a Zn–Ru vector. Isosurfaces are generated for σ = 0.3 au and −0.07 < ρ < 0.07 au; a key showing the colour scheme employed is also provided.

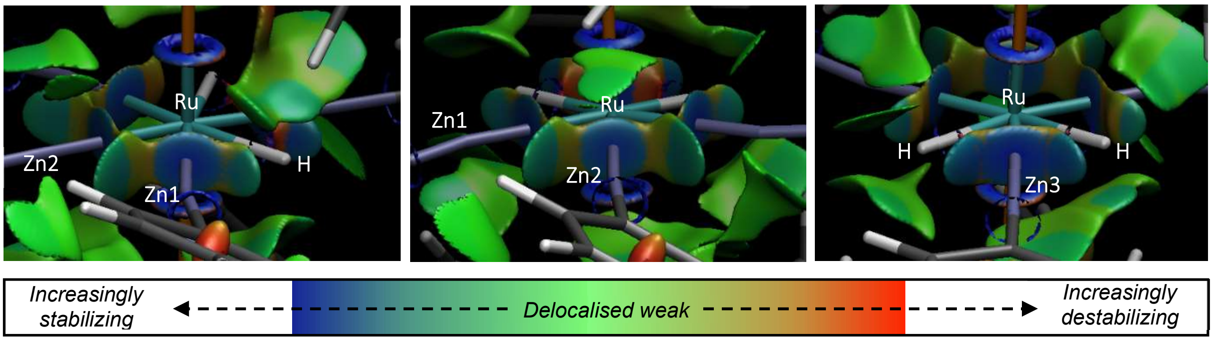

Figure S35. NCI plots of **2b** centred on, from left to right, the vectors Zn1–Ru, Zn2–Ru and Zn3–Ru. Isosurfaces are generated for σ = 0.3 au and −0.07 < ρ < 0.07 au; a key showing the colour scheme employed is also provided.

S2-3 Computed Structures (A) and Energies (atomic units).

1a (H atoms optimised)
BP86 energy = -2567.98202449

```
Ru -0.00000  0.00000 -0.00000
H   0.09060 -0.08018 -1.69081
Zn -0.20875 -2.09776 -1.26106
Zn  0.13764  1.99316 -1.44606
P   2.31865 -0.10132 -0.01745
C  -0.57913 -3.56544 -2.50573
H  -1.66336 -3.75956 -2.59236
H  -0.09192 -4.49769 -2.16864
H  -0.20243 -3.33235 -3.51811
C   0.23754  3.32175 -2.88824
H  -0.53190  3.13804 -3.65936
H   1.23006  3.28274 -3.37033
H   0.08702  4.34298 -2.49553
C   3.15506  1.25807 -0.95660
C   3.09485  2.55084 -0.43392
H   2.62954  2.72426  0.54429
C   3.61719  3.63126 -1.12315
H   3.54728  4.63538 -0.69150
C   4.22529  3.43398 -2.35372
H   4.63364  4.28619 -2.90808
C   4.32934  2.16061 -2.86392
H   4.82743  1.99316 -3.82588
C   3.80178  1.07336 -2.17583
H   3.90838  0.07175 -2.60065
C   3.28027 -0.00129  1.55395
C   2.69309 -0.27246  2.78358
H   1.62269 -0.49493  2.83636
C   3.45190 -0.24732  3.95421
H   2.96871 -0.45924  4.91408
C   4.80012  0.04346  3.89874
H   5.39419  0.06261  4.81955
C   5.39528  0.31781  2.68662
H   6.46375  0.55567  2.63620
C   4.64434  0.29988  1.52391
H   5.13052  0.52224  0.56791
C   3.05232 -1.60332 -0.79981
C   3.96517 -2.42387 -0.15066
H   4.31109 -2.17684  0.85667
C   4.43827 -3.57045 -0.76391
H   5.14811 -4.20948 -0.22598
C   4.02444 -3.91770 -2.03199
H   4.40399 -4.83056 -2.50425
C   3.13522 -3.11168 -2.69537
H   2.79798 -3.36749 -3.70583
C   2.64904 -1.96801 -2.08863
H   1.93965 -1.33443 -2.63474
H  -0.09061  0.08018  1.69080
Zn  0.20875  2.09776  1.26105
Zn -0.13763 -1.99315  1.44607
P  -2.31864  0.10132  0.01746
C   0.57913  3.56543  2.50572
H   1.66330  3.76023  2.59161
H   0.09109  4.49744  2.16917
H   0.20331  3.33191  3.51833
C  -0.23755 -3.32176  2.88825
H   0.53224 -3.13836  3.65910
H  -1.22988 -3.28237  3.37069
H  -0.08757 -4.34303  2.49546
C  -3.15506 -1.25807  0.95660
C  -3.09484 -2.55084  0.43393
H  -2.62953 -2.72427 -0.54429
C  -3.61720 -3.63126  1.12316
H  -3.54728 -4.63538  0.69151
C  -4.22529 -3.43398  2.35372
H  -4.63365 -4.28619  2.90807
C  -4.32933 -2.16061  2.86392
H  -4.82742 -1.99316  3.82589
C  -3.80178 -1.07336  2.17583
H  -3.90837 -0.07175  2.60065
C  -3.28027  0.00129 -1.55396
C  -2.69309  0.27246 -2.78359
H  -1.62269  0.49493 -2.83637
C  -3.45190  0.24732 -3.95421
H  -2.96872  0.45924 -4.91409
C  -4.80012 -0.04346 -3.89873
H  -5.39419 -0.06261 -4.81954
C  -5.39528 -0.31781 -2.68661
H  -6.46375 -0.55567 -2.63620
C  -4.64434 -0.29988 -1.52390
H  -5.13051 -0.52224 -0.56790
C  -3.05232  1.60332  0.79980
C  -3.96517  2.42388  0.15065
H  -4.31110  2.17685 -0.85667
C  -4.43826  3.57045  0.76391
H  -5.14810  4.20949  0.22599
C  -4.02445  3.91770  2.03199
H  -4.40400  4.83055  2.50424
C  -3.13522  3.11168  2.69537
H  -2.79798  3.36750  3.70583
C  -2.64904  1.96801  2.08863
H  -1.93965  1.33443  2.63474
```

1a (fully optimised)
BP86 energy = -2568.00514498
Enthalpy 0K = -2567.316704
Enthalpy 298K = -2567.259012
Free energy 298K = -2567.418155
Low Freq. = 55.9691 cm-1

```
Ru  0.00001  0.00429 -0.00005
H   0.09232  0.02789  1.69290
Zn -0.26368  2.11524  1.31571
Zn -0.06168 -2.05540  1.38810
P   2.35493 -0.01132  0.05866
C  -0.61598  3.56745  2.60030
H  -0.36195  3.26190  3.63114
H  -1.68165  3.85496  2.57899
H  -0.01308  4.45634  2.34502
C  -0.21095 -3.51086  2.70698
H  -1.17561 -3.45895  3.24198
H   0.60281 -3.43711  3.44961
H  -0.13914 -4.49825  2.21776
C   3.13598  1.52674  0.81656
C   3.98706  2.38435  0.09058
H   4.27290  2.13257 -0.93457
C   4.46977  3.57124  0.67059
```

H	5.11975	4.23027	0.08503
C	4.12645	3.90956	1.98761
H	4.50652	4.83231	2.43791
C	3.28559	3.05788	2.72437
H	3.00789	3.31028	3.75300
C	2.78429	1.88481	2.13969
H	2.11531	1.23758	2.71862
C	3.20167	-1.38615	1.04787
C	3.00848	-2.71821	0.61573
H	2.39899	-2.92140	-0.27343
C	3.60310	-3.79109	1.29530
H	3.44263	-4.81336	0.93734
C	4.39546	-3.55302	2.43162
H	4.85187	-4.38986	2.97035
C	4.60119	-2.23584	2.86524
H	5.22451	-2.03656	3.74367
C	4.01755	-1.15808	2.17378
H	4.20972	-0.13752	2.51581
C	3.34114	-0.18203	-1.53814
C	2.72554	-0.01364	-2.79316
H	1.65259	0.19722	-2.84267
C	3.47006	-0.13080	-3.98006
H	2.97119	-0.00030	-4.94601
C	4.84125	-0.42037	-3.92505
H	5.42158	-0.51667	-4.84870
C	5.46602	-0.59366	-2.67781
H	6.53524	-0.82523	-2.62549
C	4.72271	-0.47758	-1.49404
H	5.21816	-0.62289	-0.52856
H	-0.09219	0.02738	-1.69305
Zn	0.06118	-2.05523	-1.38841
Zn	0.26399	2.11530	-1.31564
P	-2.35491	-0.01113	-0.05865
C	0.21003	-3.51129	-2.70665
H	1.17389	-3.45882	-3.24304
H	0.13990	-4.49839	-2.21661
H	-0.60485	-3.43889	-3.44817
C	0.61632	3.56753	-2.60021
H	0.36136	3.26241	-3.63095
H	0.01419	4.45677	-2.34429
H	1.68219	3.85435	-2.57957
C	-3.34104	-0.18195	1.53818
C	-2.72538	-0.01376	2.79319
H	-1.65241	0.19702	2.84267
C	-3.46985	-0.13103	3.98011
H	-2.97093	-0.00068	4.94606
C	-4.84106	-0.42050	3.92513
H	-5.42136	-0.51688	4.84880
C	-5.46590	-0.59358	2.67790
H	-6.53514	-0.82507	2.62560
C	-4.72264	-0.47740	1.49410
H	-5.21815	-0.62256	0.52863
C	-3.20165	-1.38592	-1.04791
C	-4.01681	-1.15788	-2.17434
H	-4.20855	-0.13734	-2.51667
C	-4.60032	-2.23564	-2.86591
H	-5.22308	-2.03638	-3.74474
C	-4.39516	-3.55278	-2.43190
H	-4.85148	-4.38962	-2.97071
C	-3.60351	-3.79081	-1.29507
H	-3.44350	-4.81305	-0.93683
C	-3.00902	-2.71795	-0.61538
H	-2.40010	-2.92111	0.27417
C	-3.13600	1.52699	-0.81641
C	-3.98731	2.38439	-0.09045
H	-4.27329	2.13244	0.93461
C	-4.47007	3.57130	-0.67039
H	-5.12021	4.23018	-0.08483
C	-4.12657	3.90985	-1.98729
H	-4.50667	4.83262	-2.43754
C	-3.28547	3.05838	-2.72403
H	-3.00761	3.31096	-3.75257
C	-2.78411	1.88530	-2.13943
H	-2.11494	1.23824	-2.71833

TS(1a-1b)

BP86 energy = -2567.98599833
Enthalpy 0K = -2567.297382
Enthalpy 298K = -2567.240316
Free energy 298K = -2567.397689
Low Freq. = -110.8178 cm-1
Second Freq. = 10.1313 cm-1

Ru	0.05670	-0.29425	-0.14993
H	0.62424	-1.15885	-1.45801
Zn	0.14135	1.32568	-2.04973
Zn	-1.08565	-1.48298	-2.04101
P	-2.22835	0.08368	0.33788
C	0.32583	2.48810	-3.64215
H	0.17573	3.55060	-3.37986
H	-0.41980	2.21811	-4.41155
H	1.32893	2.37948	-4.09343
C	-2.03966	-2.35749	-3.51064
H	-2.08550	-1.70052	-4.39680
H	-3.06826	-2.61965	-3.21284
H	-1.51216	-3.28398	-3.79908
C	-3.50761	-1.23819	-0.14080
C	-3.28903	-2.55672	0.31789
H	-2.40870	-2.78103	0.93235
C	-4.18726	-3.58758	0.00839
H	-3.99697	-4.60061	0.37798
C	-5.32291	-3.31957	-0.77575
H	-6.02015	-4.12488	-1.02919
C	-5.55851	-2.01258	-1.22622
H	-6.44420	-1.79032	-1.83119
C	-4.66360	-0.97572	-0.90412
H	-4.87126	0.03870	-1.25558
C	-2.73233	0.25500	2.15419
C	-1.77631	0.48640	3.16071
H	-0.71732	0.53515	2.88524
C	-2.16605	0.63863	4.50278
H	-1.40545	0.81832	5.27008
C	-3.52120	0.55707	4.85586
H	-3.82679	0.67070	5.90132
C	-4.48474	0.32355	3.85993
H	-5.54498	0.25321	4.12556
C	-4.09445	0.17283	2.52079
H	-4.85524	-0.01644	1.75680
C	-3.01500	1.62826	-0.40343
C	-3.46843	2.69911	0.39378
H	-3.42873	2.62514	1.48399
C	-3.96623	3.87248	-0.20099

```
H  -4.29975  4.69797  0.43678
C  -4.03648  3.98687 -1.59680
H  -4.42701  4.90010 -2.05748
C  -3.59771  2.92135 -2.40179
H  -3.64878  2.99506 -3.49302
C  -3.08067  1.75928 -1.80995
H  -2.72938  0.94306 -2.45314
H   0.06985 -0.81697  1.42496
Zn  0.19117 -2.64957  0.58364
Zn  0.09534  2.04202  0.73632
P   2.40082 -0.06895  0.12196
C   0.37232 -4.52883  1.14137
H  -0.08850 -5.20808  0.40242
H   1.43728 -4.80186  1.24370
H  -0.11714 -4.69784  2.11743
C   0.09020  3.83324  1.56158
H  -0.28932  4.58056  0.84306
H  -0.55650  3.85143  2.45611
H   1.11435  4.11821  1.85705
C   3.13367  1.64724  0.41578
C   3.00557  2.63711 -0.58726
H   2.53540  2.39048 -1.54698
C   3.48428  3.93882 -0.37951
H   3.37951  4.68556 -1.17342
C   4.08516  4.28290  0.84380
H   4.44885  5.30190  1.01142
C   4.21352  3.31150  1.84731
H   4.68062  3.56756  2.80435
C   3.75030  2.00114  1.63356
H   3.86941  1.25391  2.42304
C   3.48207 -0.68114 -1.30113
C   3.18675 -1.93140 -1.88704
H   2.32341 -2.50474 -1.53426
C   3.98172 -2.44879 -2.92015
H   3.73400 -3.42196 -3.35726
C   5.08530 -1.72013 -3.39453
H   5.70027 -2.11791 -4.20856
C   5.39436 -0.48092 -2.81524
H   6.25507  0.09489 -3.17213
C   4.60517  0.03188 -1.77031
H   4.86995  0.99406 -1.32356
C   3.19786 -1.00775  1.55783
C   4.50563 -1.52978  1.45856
H   5.06972 -1.41925  0.52798
C   5.09297 -2.19851  2.54514
H   6.10412 -2.60720  2.44398
C   4.39050 -2.34535  3.75149
H   4.84889 -2.87002  4.59632
C   3.09397 -1.81862  3.86563
H   2.53447 -1.92731  4.80089
C   2.50203 -1.15976  2.77557
H   1.48393 -0.76654  2.86244
```

1b

```
BP86 energy =      -2568.00514880
Enthalpy 0K =      -2567.316768
Enthalpy 298K =    -2567.258900
Free energy 298K = -2567.421020
Low Freq. = 7.9580 cm-1

Ru  0.00374 -0.18841 -0.00296
H  -0.00294 -1.17526  1.36541
H   0.03100 -1.20929 -1.35508
Zn  0.03653 -2.66207 -0.05590
Zn -0.01244  0.81669  2.27373
Zn  0.01379  2.32810 -0.05931
Zn -0.03862  0.78901 -2.29532
P   2.36127 -0.16546  0.01455
P  -2.35430 -0.17303  0.03354
C  -0.06830  1.25582 -4.21227
C   3.21104 -1.42284 -1.11622
C   4.29816 -2.21400 -0.68824
H   4.68429 -2.11007  0.32925
C   4.89161 -3.14732 -1.55566
H   5.72573 -3.76008 -1.19721
C   4.42227 -3.29508 -2.86925
H   4.88726 -4.02170 -3.54368
C   3.34677 -2.50706 -3.31090
H   2.96920 -2.61039 -4.33376
C   2.74191 -1.58897 -2.43869
H   1.88733 -1.00085 -2.79032
C  -3.89526  1.52146 -1.72553
C  -4.46209  2.74471 -2.12401
C  -3.22189  1.41261 -0.49152
C  -4.38001  3.86989 -1.28982
H  -4.82676  4.82029 -1.59953
C  -3.13807  2.55414  0.33823
C  -3.72119  3.76912 -0.05295
H  -2.62729  2.49108  1.30619
H  -3.65248  4.63858  0.60896
H  -4.97621  2.81105 -3.08882
H  -3.98318  0.64855 -2.37925
C  -4.50124 -1.98569 -0.68261
C  -5.15799 -2.90324 -1.51866
C  -3.25585 -1.43489 -1.05523
C  -4.58958 -3.27869 -2.74609
H  -5.10261 -3.99579 -3.39548
C  -2.69354 -1.82330 -2.28982
C  -3.35594 -2.73169 -3.13199
H  -1.72217 -1.41879 -2.59169
H  -2.89972 -3.01539 -4.08636
H  -6.11811 -3.32678 -1.20472
H  -4.96160 -1.70458  0.26878
C  -2.65759 -1.46579  2.56519
C  -3.29069 -1.78226  3.77746
C  -3.21019 -0.51087  1.68545
C  -4.48742 -1.14206  4.13706
H  -4.97690 -1.38027  5.08716
C  -4.42192  0.11599  2.05158
C  -5.05044 -0.19324  3.26984
H  -4.88044  0.85147  1.38454
H  -5.98508  0.31124  3.53760
H  -2.84128 -2.52609  4.44394
H  -1.71710 -1.95986  2.30168
C   0.05978 -4.61685 -0.00726
C  -0.02088  1.41273  4.15155
C   0.02519  4.30452 -0.13851
C   4.54545 -0.00929  1.90311
C   5.21010 -0.31715  3.10131
C   3.25471 -0.52189  1.64172
C   4.60160 -1.14827  4.05557
H   5.11894 -1.38370  4.99154
```

```
C   2.65807 -1.36014  2.60572
C   3.32500 -1.67317  3.80181
H   1.65813 -1.76496  2.42025
H   2.83919 -2.32272  4.53760
H   6.20730  0.09610  3.28713
H   5.03631  0.63474  1.16759
C   3.16583  2.53844  0.38659
C   3.75642  3.76178  0.03576
C   3.23442  1.42705 -0.48451
C   4.41102  3.90083 -1.19972
H   4.86471  4.85776 -1.47765
C   3.90494  1.57296 -1.71572
C   4.48151  2.80475 -2.07259
H   3.98679  0.72262 -2.39916
H   4.99483  2.89994 -3.03545
H   3.69809  4.60771  0.72845
H   2.66237  2.44362  1.35578
H  -0.96235  1.86043 -4.44554
H  -0.08726  0.35446 -4.85118
H   0.82386  1.84917 -4.47966
H   0.02560  4.74467  0.87547
H  -0.86883  4.66648 -0.67448
H   0.92564  4.65618 -0.67043
H  -0.85331  2.11468  4.33604
H   0.92431  1.92528  4.40351
H  -0.13936  0.55332  4.83468
H   0.40770 -5.02079 -0.97351
H  -0.95162 -5.01242  0.18973
H   0.73682 -4.98285  0.78397
```

TS(1b-1c)

```
BP86 energy =      -2567.98829962
Enthalpy 0K =      -2567.298912
Enthalpy 298K =    -2567.242193
Free energy 298K = -2567.397525
Low Freq. =  -87.9604 cm-1
Second Freq. = 7.6188 cm-1

Ru -0.06879  0.23142  0.29323
H  -0.01028 -0.28123  1.87434
H  -0.66211  1.62347  1.02837
Zn  0.93492  2.32666  1.30316
Zn -0.16441 -2.14154  0.95688
Zn -0.08712 -1.05784 -1.84661
Zn -0.24176  1.73362 -1.69909
P  -2.43096  0.07335  0.26047
P   2.27395 -0.11645  0.12431
C  -0.38683  3.03767 -3.18378
C  -3.41231  1.49145 -0.51841
C  -4.57938  1.29042 -1.28650
H  -4.93270  0.27658 -1.49474
C  -5.29982  2.38443 -1.79622
H  -6.19764  2.20447 -2.39740
C  -4.87657  3.69617 -1.53603
H  -5.43829  4.54713 -1.93504
C  -3.72627  3.90888 -0.75911
H  -3.38562  4.92718 -0.54430
C  -2.99957  2.81701 -0.26008
H  -2.10166  2.99720  0.33970
C   3.63819 -0.90070 -2.32580
C   4.04950 -1.83229 -3.29617
C   2.91330 -1.31879 -1.19222
C   3.75775 -3.19499 -3.14070
H   4.08121 -3.91885 -3.89579
C   2.62521 -2.69568 -1.04581
C   3.04834 -3.62545 -2.00618
H   2.07837 -3.05327 -0.16490
H   2.81814 -4.68696 -1.86794
H   4.60621 -1.48467 -4.17299
H   3.89343  0.15445 -2.45656
C   3.08712  2.24212 -1.29056
C   3.87145  3.37404 -1.56043
C   3.39717  1.37730 -0.21535
C   4.97593  3.67474 -0.74608
H   5.58188  4.56473 -0.94524
C   4.51773  1.68437  0.58568
C   5.29317  2.82761  0.32624
H   4.78465  1.03794  1.42558
H   6.14881  3.05403  0.97142
H   3.61083  4.02253 -2.40329
H   2.21982  2.03343 -1.92830
C   2.62339 -0.62765  2.92612
C   3.28917 -1.11373  4.06388
C   3.14974 -0.84008  1.63629
C   4.49052 -1.82609  3.92726
H   5.00582 -2.21368  4.81243
C   4.36501 -1.55019  1.51073
C   5.02659 -2.04237  2.64694
H   4.79777 -1.72152  0.52044
H   5.96347 -2.59739  2.52903
H   2.86081 -0.94030  5.05687
H   1.67275 -0.09447  3.03232
C   1.80770  3.84467  2.17315
C  -0.22655 -3.98374  1.64815
C  -0.08282 -2.05205 -3.55291
C  -2.58776 -0.01356  3.13622
C  -3.25345 -0.03526  4.37487
C  -3.31503  0.03393  1.93281
C  -4.65459 -0.00909  4.42324
H  -5.17405 -0.02418  5.38728
C  -4.72656  0.06725  1.99143
C  -5.39013  0.04230  3.22621
H  -5.31279  0.11887  1.06843
H  -6.48482  0.06759  3.25356
H  -2.67049 -0.07069  5.30161
H  -1.49382 -0.02874  3.09302
C  -3.20446 -1.52814 -1.98162
C  -3.66988 -2.69249 -2.61281
C  -3.18412 -1.43170 -0.57123
C  -4.10510 -3.78601 -1.84644
H  -4.46141 -4.69639 -2.33943
C  -3.62879 -2.53455  0.18930
C  -4.08054 -3.70389 -0.44548
H  -3.62905 -2.48065  1.28212
H  -4.41785 -4.55014  0.16228
H  -3.68391 -2.74339 -3.70633
H  -2.86684 -0.68534 -2.59461
H  -0.11084  4.05545 -2.85297
H  -1.42330  3.07288 -3.56341
H   0.27532  2.75686 -4.02315
H  -0.52946 -3.05293 -3.42336
H   0.95358 -2.17168 -3.91232
```

```
H  -0.66031 -1.50944 -4.32275
H  -0.99837 -4.07464  2.43290
H   0.74471 -4.26591  2.09232
H  -0.46529 -4.70210  0.84476
H   2.15210  3.56827  3.18479
H   1.09730  4.68478  2.26803
H   2.67868  4.18254  1.58793
```

1c

```
BP86 energy =      -2568.00197954
Enthalpy 0K =      -2567.312488
Enthalpy 298K =    -2567.254895
Free energy 298K = -2567.414909
Low Freq. = 10.6650 cm-1

Ru -0.00001 -0.00008 -0.17253
H   0.09776  1.01456 -1.49999
H  -0.09780 -1.01475 -1.49997
Zn -0.22239  2.49660 -0.35485
Zn  0.49541  1.25387  1.96318
Zn -0.49566 -1.25404  1.96309
Zn  0.22265 -2.49673 -0.35482
P  -2.35298  0.00450 -0.32912
P   2.35298 -0.00454 -0.32907
C   0.55500 -4.41885 -0.57983
C  -3.43126  0.40454  1.16569
C  -4.66537 -0.23899  1.40224
H  -5.01519 -1.01856  0.71945
C  -5.45140  0.10975  2.51372
H  -6.40087 -0.40823  2.68671
C  -5.02524  1.11423  3.39667
H  -5.63772  1.38160  4.26409
C  -3.80782  1.77373  3.16110
H  -3.46474  2.56162  3.83960
C  -3.01865  1.41681  2.05702
H  -2.06558  1.93041  1.88392
C   4.28567 -1.89146 -1.38491
C   4.79673 -2.75631 -2.36749
C   3.08635 -1.18357 -1.60797
C   4.12747 -2.91429 -3.59071
H   4.52711 -3.58990 -4.35426
C   2.41807 -1.35130 -2.84159
C   2.93883 -2.20408 -3.82764
H   1.47904 -0.81741 -3.01911
H   2.40742 -2.32015 -4.77832
H   5.72227 -3.30822 -2.17145
H   4.82308 -1.77464 -0.43952
C   3.02849  2.75172 -0.02157
C   3.52546  4.00220 -0.41955
C   3.10938  1.63250 -0.88177
C   4.10066  4.15985 -1.69215
H   4.48008  5.13731 -2.00752
C   3.70397  1.79831 -2.14812
C   4.18821  3.05548 -2.55206
H   3.79125  0.94508 -2.82676
H   4.64009  3.16479 -3.54385
H   3.45791  4.85315  0.26610
H   2.59263  2.64706  0.97980
C   4.66557  0.23875  1.40201
C   5.45169 -0.10999  2.51342
C   3.43132 -0.40460  1.16570
C   5.02547 -1.11429  3.39655
H   5.63803 -1.38166  4.26392
C   3.01866 -1.41668  2.05721
C   3.80791 -1.77361  3.16122
H   2.06548 -1.93014  1.88428
H   3.46478 -2.56135  3.83987
H   6.40127  0.40784  2.68623
H   5.01543  1.01819  0.71908
C  -0.55455  4.41877 -0.57979
C   1.03583  2.17095  3.63173
C  -1.03622 -2.17086  3.63174
C  -3.02835 -2.75183 -0.02199
C  -3.52546 -4.00223 -0.42005
C  -3.10951 -1.63245 -0.88195
C  -4.10106 -4.15964 -1.69249
H  -4.48058 -5.13704 -2.00794
C  -3.70451 -1.79801 -2.14813
C  -4.18888 -3.05511 -2.55216
H  -3.79199 -0.94466 -2.82659
H  -4.64107 -3.16422 -3.54383
H  -3.45769 -4.85332  0.26542
H  -2.59216 -2.64738  0.97926
C  -4.28552  1.89168 -1.38475
C  -4.79655  2.75665 -2.36724
C  -3.08628  1.18369 -1.60792
C  -4.12733  2.91465 -3.59049
H  -4.52693  3.59036 -4.35397
C  -2.41806  1.35143 -2.84156
C  -2.93878  2.20433 -3.82753
H  -1.47910  0.81744 -3.01918
H  -2.40742  2.32040 -4.77823
H  -5.72202  3.30864 -2.17112
H  -4.82290  1.77484 -0.43934
H  -0.04186 -4.81136 -1.42152
H   0.28378 -4.98159  0.33013
H   1.62253 -4.59842 -0.79552
H  -0.51050 -1.73440  4.49970
H  -0.79527 -3.24864  3.59245
H  -2.12182 -2.06546  3.80575
H   0.79516  3.24878  3.59208
H   2.12136  2.06534  3.80601
H   0.50982  1.73487  4.49970
H   0.04223  4.81123 -1.42155
H  -0.28312  4.98145  0.33015
H  -1.62209  4.59847 -0.79530
```

2a

```
BP86 energy =      -3334.92676340
Enthalpy 0K =      -3334.027168
Enthalpy 298K =    -3333.956942
Free energy 298K = -3334.151118
Low Freq. = 7.6439 cm-1

Ru  0.00002 -0.00002 -0.00023
H  -0.00119 -0.80228  1.49618
Zn  2.09768 -0.87459  0.99901
Zn -2.05828 -0.78755  1.13827
P  -0.14126 -2.00345 -1.25665
C  -1.54739 -2.13218 -2.51372
C  -1.33970 -2.25370 -3.90251
H  -0.32435 -2.30113 -4.30493
```

C -2.43263 -2.32630 -4.78581
H -2.24841 -2.41299 -5.86208
C -3.74572 -2.29690 -4.29503
H -4.59463 -2.35331 -4.98409
C -3.96477 -2.19973 -2.91044
H -4.98329 -2.18477 -2.50998
C -2.87618 -2.11404 -2.03035
H -3.07239 -2.05569 -0.95243
C 1.35511 -2.39555 -2.33145
C 2.09950 -3.58388 -2.18630
H 1.78543 -4.34265 -1.46442
C 3.25369 -3.80153 -2.96001
H 3.82402 -4.72689 -2.82547
C 3.67138 -2.84611 -3.89856
H 4.57007 -3.01857 -4.49961
C 2.93159 -1.66253 -4.05938
H 3.24892 -0.90437 -4.78196
C 1.78920 -1.43613 -3.27599
H 1.22527 -0.50532 -3.40903
C -0.37741 -3.64292 -0.36133
C 0.05747 -3.80250 0.96979
H 0.49244 -2.95363 1.50607
C -0.06044 -5.04235 1.62108
H 0.29143 -5.14498 2.65275
C -0.62549 -6.13819 0.95109
H -0.72445 -7.10314 1.45915
C -1.06520 -5.99021 -0.37494
H -1.50977 -6.83901 -0.90528
C -0.93955 -4.75464 -1.02823
H -1.28344 -4.65400 -2.06212
H 0.00109 0.80215 -1.49664
Zn -2.09779 0.87470 -0.99903
Zn 2.05838 0.78741 -1.13857
P 0.14138 2.00338 1.25625
C 0.37654 3.64306 0.36105
C -0.05782 3.80243 -0.97025
H -0.49187 2.95326 -1.50681
C 0.05952 5.04242 -1.62139
H -0.29191 5.14488 -2.65323
C 0.62346 6.13861 -0.95104
H 0.72194 7.10368 -1.45897
C 1.06268 5.99083 0.37518
H 1.50642 6.83990 0.90578
C 0.93764 4.75511 1.02829
H 1.28120 4.65459 2.06231
C 1.54829 2.13243 2.51242
C 1.34150 2.25325 3.90140
H 0.32641 2.30003 4.30456
C 2.43503 2.32598 4.78397
H 2.25151 2.41211 5.86040
C 3.74778 2.29740 4.29226
H 4.59715 2.35387 4.98075
C 3.96590 2.20095 2.90747
H 4.98415 2.18665 2.50629
C 2.87674 2.11514 2.02810
H 3.07223 2.05736 0.95001
C -1.35445 2.39481 2.33204
C -1.78767 1.43503 3.27664
H -1.22341 0.50434 3.40904
C -2.92959 1.66093 4.06084
H -3.24624 0.90252 4.78345
C -3.66982 2.84434 3.90080
H -4.56816 3.01640 4.50248
C -3.25303 3.80009 2.96219
H -3.82371 4.72532 2.82824
C -2.09930 3.58294 2.18766
H -1.78591 4.34198 1.46574
H -4.41384 -4.48846 3.83702
H -2.48279 -3.43288 2.69833
C -4.53826 -3.51388 3.34993
C -3.44544 -2.90617 2.70622
C -5.78911 -2.87583 3.36581
H -6.64290 -3.34841 3.86446
C -3.56702 -1.64733 2.06723
C -5.93992 -1.62988 2.73511
C -4.84154 -1.02806 2.09665
H -6.91436 -1.12697 2.73961
H -4.98663 -0.05258 1.61441
H -4.73909 2.35717 -5.15282
H -2.95760 1.12361 -3.95095
C -4.66539 2.43706 -4.06153
C -3.65428 1.74431 -3.37287
C -5.58306 3.22948 -3.35232
H -6.37317 3.76955 -3.88619
C -3.52671 1.82373 -1.96379
C -5.48269 3.32397 -1.95482
C -4.46639 2.63000 -1.27450
H -6.19660 3.93945 -1.39434
H -4.40843 2.72398 -0.18215
H 6.19975 -3.93482 1.39663
H 4.41238 -2.71952 0.18314
C 5.48398 -3.32125 1.95681
C 4.46811 -2.62737 1.27576
C 5.58154 -3.22909 3.35467
H 6.37131 -3.76908 3.88911
C 3.52607 -1.82353 1.96464
C 4.66149 -2.43910 4.06350
C 3.65081 -1.74644 3.37411
H 4.73299 -2.36103 5.15507
H 2.95221 -1.12767 3.95194
H 4.41439 4.49073 -3.83364
H 2.48349 3.43472 -2.69512
C 4.53853 3.51532 -3.34815
C 3.44578 2.90737 -2.70452
C 5.78892 2.87643 -3.36600
H 6.64265 3.34919 -3.86459
C 3.56699 1.64746 -2.06757
C 5.93935 1.62940 -2.73737
C 4.84105 1.02735 -2.09899
H 6.91343 1.12581 -2.74343
H 4.98582 0.05101 -1.61841

TS(2a-2b)
BP86 energy = -3334.90748332
Enthalpy 0K = -3334.007990
Enthalpy 298K = -3333.938257
Free energy 298K = -3334.131468
Low Freq. = -106.8244 cm-1
Second Freq. = 8.8119 cm-1

Ru -0.20512 0.29745 -0.06794
H -0.94324 0.28193 1.42491

```
Zn  1.23864 -1.12488  1.38257
Zn -1.72925 -1.33852  1.03937
P  -0.44198 -1.37850 -1.74067
C  -2.13284 -2.20246 -1.99656
C  -3.25066 -1.35933 -2.18482
H  -3.12882 -0.26980 -2.15113
C  -4.52292 -1.89110 -2.44096
H  -5.37041 -1.21339 -2.58633
C  -4.70136 -3.28392 -2.50845
H  -5.69407 -3.70354 -2.70174
C  -3.59868 -4.13175 -2.32853
H  -3.72581 -5.21830 -2.37908
C  -2.32111 -3.59755 -2.08233
H  -1.47304 -4.27592 -1.95521
C  -0.16054 -0.88387 -3.54392
C   0.36170  0.37473 -3.89161
H   0.59528  1.08965 -3.09545
C   0.57019  0.72064 -5.23831
H   0.97825  1.70579 -5.48750
C   0.25693 -0.19230 -6.25537
H   0.41670  0.07527 -7.30510
C  -0.26783 -1.45234 -5.91989
H  -0.52027 -2.17072 -6.70703
C  -0.47811 -1.79487 -4.57647
H  -0.89926 -2.77502 -4.33121
C   0.69106 -2.87050 -1.58272
C   1.76310 -3.08488 -2.47339
H   1.91301 -2.41586 -3.32512
C   2.65591 -4.15193 -2.26885
H   3.49070 -4.29254 -2.96323
C   2.48153 -5.02623 -1.18593
H   3.17520 -5.85928 -1.03190
C   1.41229 -4.82466 -0.29577
H   1.26533 -5.49672  0.55551
C   0.53131 -3.74911 -0.48586
H  -0.29165 -3.60467  0.22540
H  -0.61117  1.28825 -1.33500
Zn -2.38407  1.39056 -0.36263
Zn  2.11342  0.21731 -0.96989
P   0.63671  2.28117  0.93948
C   2.50311  2.51697  1.06794
C   3.26728  1.59418  1.82031
H   2.77541  0.77903  2.36483
C   4.66289  1.70929  1.89422
H   5.23157  0.98423  2.48522
C   5.32275  2.74168  1.20616
H   6.41343  2.82356  1.25102
C   4.57555  3.66083  0.45638
H   5.07993  4.46721 -0.08632
C   3.17487  3.55677  0.39294
H   2.60777  4.28763 -0.19008
C   0.09624  2.60293  2.71844
C  -1.27255  2.47861  3.04333
H  -1.99290  2.17835  2.27479
C  -1.73010  2.73930  4.34301
H  -2.79682  2.64023  4.56947
C  -0.82396  3.12129  5.34673
H  -1.17843  3.31462  6.36455
C   0.53636  3.25673  5.03416
H   1.25201  3.55911  5.80615
C   0.99398  3.00831  3.72762
H   2.05650  3.13025  3.50068
C   0.14483  3.92289  0.14702
C  -0.01796  5.08867  0.92633
H   0.10656  5.04250  2.01208
C  -0.34623  6.31245  0.32076
H  -0.48043  7.20331  0.94342
C  -0.50477  6.39485 -1.07165
H  -0.76297  7.34966 -1.54147
C  -0.33365  5.24377 -1.85757
H  -0.45367  5.29514 -2.94503
C  -0.01560  4.01728 -1.25097
H   0.10035  3.11807 -1.86417
H   2.22228 -2.52556  6.20935
H   1.02948 -1.30807  4.41174
C   2.52742 -2.66507  5.16535
C   1.85607 -1.97813  4.13762
C   3.58900 -3.52928  4.85271
H   4.11580 -4.06629  5.64955
C   2.21822 -2.13340  2.77560
C   3.97039 -3.70284  3.51165
C   3.29111 -3.01363  2.49170
H   4.79762 -4.37794  3.26096
H   3.60303 -3.17632  1.45179
H   6.89167 -1.50977 -1.74003
H   4.66350 -1.47423 -0.65869
C   6.14747 -0.76859 -2.05569
C   4.88059 -0.74121 -1.44655
H   7.44613  0.13438 -3.54629
C   6.46019  0.15312 -3.06818
C   3.89277  0.20021 -1.82554
C   5.50048  1.09862 -3.46428
C   4.23666  1.11731 -2.84812
H   5.73680  1.82208 -4.25401
H   3.50646  1.86798 -3.17817
H  -5.75022 -4.47466  1.73125
H  -4.43861 -2.76506  0.50421
C  -4.78308 -4.14446  2.12866
C  -4.03742 -3.17214  1.44008
H  -4.87176 -5.45240  3.86163
C  -4.29126 -4.69409  3.32417
C  -2.78584 -2.72168  1.92667
C  -3.05096 -4.26812  3.82573
C  -2.31014 -3.29391  3.13365
H  -2.65859 -4.69455  4.75635
H  -1.34247 -2.98107  3.54736
H  -7.45750  1.76196  0.23909
H  -5.24581  0.65955  0.42220
C  -6.56395  2.27261 -0.13921
C  -5.30508  1.65456 -0.03906
H  -7.65477  4.03045 -0.80290
C  -6.67556  3.54511 -0.72274
C  -4.12770  2.28428 -0.51463
C  -5.52495  4.19334 -1.20095
C  -4.26979  3.56890 -1.09589
H  -5.60571  5.18825 -1.65491
H  -3.38546  4.09973 -1.47078
```

2b (H atoms optimised)

BP86 energy =   -3334.76860775

```
Ru  0.42112  0.01272 -0.01490
```

H	1.45498	0.47581	1.24668
H	1.40136	-0.56678	-1.27244
Zn	2.85354	-0.08173	-0.01164
Zn	-0.55849	1.00414	1.99163
Zn	-2.05506	-0.08155	0.04861
Zn	-0.59923	-0.97454	-2.01025
P	0.37965	2.09806	-1.07089
P	0.43267	-2.05083	1.05194
C	-1.62781	-1.87672	-3.41756
C	-2.73145	-2.62016	-2.98494
H	-2.93390	-2.72972	-1.91063
C	-3.60169	-3.23294	-3.87753
H	-4.45624	-3.80613	-3.49714
C	-3.39049	-3.11889	-5.23785
H	-4.07398	-3.59947	-5.94783
C	-2.31489	-2.38583	-5.69319
H	-2.14105	-2.28115	-6.77185
C	-1.44648	-1.77350	-4.79729
H	-0.61020	-1.18559	-5.19769
C	0.32365	2.11969	-2.91998
C	-0.45401	3.01815	-3.64670
H	-1.08841	3.74910	-3.14480
C	-0.44028	2.99768	-5.03490
H	-1.06784	3.70137	-5.59337
C	0.36896	2.10254	-5.70982
H	0.37572	2.09095	-6.80589
C	1.16629	1.22844	-5.00646
H	1.82057	0.52405	-5.53264
C	1.13523	1.23092	-3.61494
H	1.76772	0.52752	-3.06233
C	-1.29550	-4.15225	0.34738
C	-2.48582	-4.77250	0.28505
C	-1.15131	-3.00495	1.07454
C	-3.57076	-4.23053	0.95084
H	-4.54583	-4.72831	0.89487
C	-2.22896	-2.45558	1.74325
C	-3.44428	-3.06642	1.68457
H	-2.08820	-1.55465	2.35331
H	-4.31023	-2.64592	2.20287
H	-2.60641	-5.69667	-0.29151
H	-0.44196	-4.58736	-0.17661
C	1.54042	-3.56840	-1.04423
C	2.48596	-4.34959	-1.59301
C	1.51171	-3.36294	0.30587
C	3.42251	-4.95890	-0.77711
H	4.19983	-5.58855	-1.22577
C	2.44284	-3.96703	1.12953
C	3.40320	-4.76808	0.59136
H	2.45045	-3.77333	2.20352
H	4.16004	-5.24285	1.22537
H	2.51519	-4.51362	-2.67609
H	0.81189	-3.06340	-1.69229
C	1.11355	-1.14389	3.71362
C	1.24397	-1.35706	5.03384
C	0.71708	-2.15573	2.88607
C	0.98903	-2.61597	5.54789
H	1.09428	-2.78940	6.62524
C	0.45993	-3.41685	3.38979
C	0.59461	-3.65276	4.72387
H	0.13299	-4.22561	2.73107
H	0.39414	-4.64507	5.14299

H	1.54952	-0.54676	5.70453
H	1.34295	-0.15969	3.28613
C	5.51951	0.41242	0.94831
C	6.85827	0.30483	0.98816
C	4.81076	-0.15421	-0.07268
C	7.51656	-0.37161	-0.02337
H	8.60969	-0.45863	0.00733
C	5.45901	-0.83201	-1.08789
C	6.81569	-0.94409	-1.06766
H	4.88575	-1.29255	-1.90319
H	7.35388	-1.47767	-1.86118
H	7.44046	0.74820	1.80652
H	4.99822	0.95139	1.75195
C	-0.26556	2.31056	4.70291
C	-0.66320	3.21369	5.61487
C	-1.05710	2.01862	3.62864
C	-1.89025	3.83527	5.46623
H	-2.21573	4.57234	6.21070
C	-2.28458	2.63409	3.47112
C	-2.70683	3.54600	4.38965
H	-2.92150	2.40647	2.60534
H	-3.67575	4.05121	4.28739
H	-0.03378	3.46400	6.47904
H	0.71438	1.82374	4.81639
C	-4.36159	-0.07240	1.51917
C	-5.65187	-0.00099	1.88731
C	-4.01740	-0.04321	0.19760
C	-6.63259	0.08599	0.91534
H	-7.68644	0.14036	1.21531
C	-4.98958	0.04358	-0.78097
C	-6.30241	0.10883	-0.42629
H	-4.70651	0.05325	-1.84196
H	-7.09277	0.17769	-1.18450
H	-5.94465	-0.00694	2.94601
H	-3.59450	-0.11081	2.30705
C	2.32125	2.81481	0.22121
C	3.34469	3.52852	0.71986
C	2.11118	2.75718	-1.12725
C	4.17624	4.21952	-0.14335
H	5.00564	4.81196	0.26083
C	2.93627	3.44380	-1.99794
C	3.97331	4.17839	-1.50959
H	2.71589	3.43352	-3.07209
H	4.64048	4.72031	-2.18823
H	3.51403	3.60102	1.80167
H	1.64863	2.32466	0.92307
C	-2.22020	3.17253	-1.10017
C	-3.11583	4.17215	-1.03638
C	-0.90537	3.40318	-0.81065
C	-2.69284	5.44317	-0.69045
H	-3.42220	6.26019	-0.64401
C	-0.47280	4.66929	-0.46399
C	-1.36520	5.69562	-0.40206
H	0.58710	4.84968	-0.25490
H	-1.04178	6.70632	-0.13014
H	-4.17121	3.98656	-1.26259
H	-2.54837	2.16673	-1.39373

2b (fully optimised)

BP86 energy = -3334.92823470

Enthalpy 0K = -3334.028152

```
Enthalpy 298K =     -3333.957956
Free energy 298K = -3334.152269
Low Freq. = 9.6415 cm-1

Ru -0.36393  0.00000 -0.00002
H  -1.33956  1.36596 -0.18018
H  -1.33960 -1.36593  0.18010
Zn -2.81982  0.00005 -0.00004
Zn  0.64263  2.24308 -0.32867
Zn  2.14793 -0.00004  0.00003
Zn  0.64252 -2.24312  0.32866
P  -0.38240  0.31349  2.34563
P  -0.38233 -0.31349 -2.34566
C   1.26615 -4.09803  0.53701
C   2.20908 -4.63928 -0.37353
H   2.58024 -4.02672 -1.20556
C   2.69054 -5.95372 -0.24117
H   3.41938 -6.34559 -0.96067
C   2.23895 -6.76402  0.81315
H   2.61310 -7.78845  0.92035
C   1.30682 -6.25272  1.73103
H   0.95235 -6.87960  2.55812
C   0.82952 -4.93742  1.59233
H   0.10393 -4.56213  2.32503
C  -0.76980 -1.18810  3.42210
C  -0.14435 -1.39715  4.67069
H   0.61822 -0.69746  5.02369
C  -0.49153 -2.49930  5.47027
H   0.01290 -2.64828  6.43089
C  -1.47887 -3.40117  5.04455
H  -1.74914 -4.25869  5.66950
C  -2.11890 -3.19503  3.81132
H  -2.89556 -3.88696  3.46915
C  -1.76269 -2.10135  3.00610
H  -2.26414 -1.95798  2.04384
C  -0.14409  1.39713 -4.67072
C  -0.49117  2.49931 -5.47031
C  -0.76959  1.18812 -3.42214
C  -1.47847  3.40124 -5.04462
H  -1.74865  4.25878 -5.66958
C  -1.76243  2.10145 -3.00617
C  -2.11854  3.19515 -3.81140
H  -2.26391  1.95811 -2.04392
H  -2.89516  3.88714 -3.46926
H   0.01330  2.64825 -6.43091
H   0.61845  0.69739 -5.02370
C   2.34608 -0.13027 -3.13718
C   3.55539 -0.58463 -3.68447
C   1.18914 -0.94217 -3.16496
C   3.63056 -1.86366 -4.26041
H   4.57668 -2.22337 -4.67763
C   1.27173 -2.21901 -3.75728
C   2.48763 -2.67695 -4.29521
H   0.38767 -2.86131 -3.80195
H   2.53410 -3.67375 -4.74662
H   4.43986  0.05819 -3.64218
H   2.30386  0.87301 -2.69691
C  -1.87994 -2.75558 -2.33536
C  -2.80218 -3.69556 -2.82243
C  -1.65614 -1.53840 -3.01639
C  -3.53111 -3.42654 -3.99272
H  -4.25908 -4.15336 -4.36798
C  -2.38482 -1.28592 -4.19841
C  -3.31996 -2.21937 -4.67633
H  -2.22916 -0.35428 -4.74911
H  -3.88425 -1.99734 -5.58833
H  -2.95122 -4.63565 -2.28104
H  -1.33082 -2.97496 -1.41366
C  -5.50686  0.91147  0.79787
C  -6.91256  0.91336  0.79986
C  -4.77124  0.00008 -0.00006
C  -7.61845  0.00012 -0.00007
H  -8.71428  0.00014 -0.00008
C  -5.50688 -0.91129 -0.79799
C  -6.91258 -0.91314 -0.79999
H  -4.97881 -1.63380 -1.43289
H  -7.45786 -1.62828 -1.42722
H  -7.45783  1.62851  1.42708
H  -4.97878  1.63396  1.43277
C   2.20918  4.63919  0.37368
C   2.69069  5.95362  0.24139
C   1.26632  4.09798 -0.53696
C   2.23921  6.76395 -0.81297
H   2.61341  7.78838 -0.92011
C   0.82981  4.93740 -1.59231
C   1.30716  6.25269 -1.73094
H   0.10429  4.56214 -2.32508
H   0.95279  6.87959 -2.55805
H   3.41947  6.34546  0.96096
H   2.58025  4.02661  1.20574
C   4.86302 -1.19313  0.19576
C   6.26950 -1.19764  0.19822
C   4.12458 -0.00006  0.00006
C   6.97647 -0.00008  0.00010
H   8.07248 -0.00009  0.00011
C   4.86305  1.19300 -0.19562
C   6.26953  1.19748 -0.19804
H   4.33707  2.14548 -0.34633
H   6.81405  2.13686 -0.35296
H   6.81400 -2.13703  0.35315
H   4.33702 -2.14561  0.34645
C  -1.87987  2.75567  2.33526
C  -2.80208  3.69570  2.82230
C  -1.65616  1.53848  3.01631
C  -3.53106  3.42673  3.99256
H  -4.25900  4.15359  4.36780
C  -2.38490  1.28604  4.19831
C  -3.32001  2.21955  4.67620
H  -2.22931  0.35439  4.74901
H  -3.88433  1.99756  5.58817
H  -2.95104  4.63580  2.28090
H  -1.33071  2.97501  1.41358
C   2.34597  0.13014  3.13724
C   3.55528  0.58444  3.68459
C   1.18907  0.94209  3.16499
C   3.63048  1.86345  4.26055
H   4.57660  2.22312  4.67781
C   1.27169  2.21891  3.75734
C   2.48759  2.67680  4.29532
H   0.38766  2.86126  3.80198
H   2.53408  3.67359  4.74674
H   4.43972 -0.05842  3.64232
```

H   2.30373 -0.87313 2.69695

ZnPh2
BP86 energy =   -690.513854392
Enthalpy 0K =   -690.339001
Enthalpy 298K =   -690.326145
Free energy 298K = -690.380333
Low Freq. = 18.1563 cm-1

C -2.66885 -0.85746 -0.85543
C -4.07386 -0.86000 -0.85759
C -4.77879 -0.00033 0.00039
C -4.07383 0.85955 0.85815
C -1.93291 0.00004 -0.00000
C -2.66883 0.85738 0.85560
H -5.87430 -0.00047 0.00056
H -4.61874 1.53255 1.52980
H -2.14351 -1.53933 -1.53601
H -4.61880 -1.53313 -1.52908
H -2.14345 1.53945 1.53595
C   1.93291 0.00045 -0.00035
C   2.66954 0.85753 -0.85557
C   2.66814 -0.85728 0.85548
C   4.07454 0.85928 -0.85736
C   4.77879 -0.00079 0.00079
C   4.07315 -0.86024 0.85840
H   2.14473 1.53974 -1.53623
H   4.62001 1.53211 -1.52872
H   5.87430 -0.00124 0.00121
H   4.61752 -1.53352 1.53021
H   2.14224 -1.53900 1.53579
Zn 0.00000 0.00047 -0.00062

ZnMePh
BP86 energy =   -498.784814066
Enthalpy 0K =   -498.662836
Enthalpy 298K =   -498.652947
Free energy 298K = -498.699824
Low Freq. = 7.5188 cm-1

H -3.09830 2.16479 0.00007
H -0.62407 2.17488 -0.00033
C -2.55316 1.21402 0.00003
C -1.14805 1.21041 0.00002
H -4.35372 -0.00081 -0.00007
C -3.25817 -0.00048 0.00003
C -0.40986 0.00043 0.00013
C -2.55246 -1.21454 -0.00003
Zn 1.52765 0.00017 -0.00008
C -1.14732 -1.21002 0.00009
H   3.86194 -0.25675 -0.99781
H -3.09701 -2.16565 -0.00029
H   3.86049 0.99220 0.27734
C   3.46619 -0.00056 -0.00017
H -0.62268 -2.17413 0.00003
H   3.86070 -0.73513 0.72275

ZnMe2
BP86 energy =   -307.055861443
Enthalpy 0K =   -306.986821
Enthalpy 298K =   -306.979865
Free energy 298K = -307.016989
Low Freq. = 14.1502 cm-1

Zn -0.00000 -0.00064 0.00006
C -1.94170 0.00098 -0.00009
H -2.33881 1.03084 0.01522
H -2.33989 -0.52692 0.88387
H -2.33968 -0.50033 -0.89949
C   1.94169 0.00087 -0.00018
H   2.33886 0.71740 -0.74006
H   2.34047 -0.99769 -0.24970
H   2.33910 0.28466 0.98995

TS1Me/Ph
BP86 energy =   -3258.50508937
Enthalpy 0K =   -3257.641171
Enthalpy 298K =   -3257.570622
Free energy 298K = -3257.757939
Low Freq. =   -33.3258 cm-1
Second Freq. = 10.4026 cm-1

Ru -0.21921 -0.61258 -0.12665
H -0.72688 -2.04545 -0.89062
Zn -0.45479 -1.14355 -2.59585
Zn -0.21399 -2.89787 0.92541
P -2.50789 -0.37983 0.41576
C -0.61161 -1.73206 -4.46283
H   0.38197 -2.01040 -4.85334
H -1.02490 -0.92288 -5.08801
H -1.27306 -2.61296 -4.54262
C -0.17756 -4.80662 1.40808
H   0.66622 -5.33086 0.92682
H -1.11895 -5.29485 1.10148
H -0.06898 -4.91848 2.50084
C -3.34642 -1.93601 1.10162
C -2.95899 -2.37188 2.39043
H -2.23114 -1.79078 2.96989
C -3.51647 -3.52626 2.95957
H -3.20505 -3.83860 3.96169
C -4.46670 -4.27608 2.24527
H -4.89680 -5.18228 2.68405
C -4.86563 -3.84839 0.97121
H -5.61497 -4.41639 0.40938
C -4.31767 -2.68189 0.40586
H -4.66530 -2.35272 -0.57690
C -3.06757 0.81605 1.75945
C -2.19214 1.78441 2.28327
H -1.16914 1.84113 1.89630
C -2.60860 2.66448 3.29789
H -1.90760 3.40752 3.69158
C -3.91548 2.58405 3.80043
H -4.24326 3.26517 4.59260
C -4.80106 1.62058 3.28646
H -5.82193 1.54868 3.67644
C -4.38092 0.74159 2.27748
H -5.07598 -0.01215 1.89348
C -3.66863 0.07354 -0.99927
C -4.45919 1.24090 -0.99316
H -4.45732 1.89923 -0.12021
C -5.24854 1.57671 -2.10736
H -5.84413 2.49546 -2.08668

```
C  -5.27386  0.74642 -3.23694
H  -5.89078  1.00971 -4.10215
C  -4.49179 -0.42109 -3.25517
H  -4.49526 -1.07610 -4.13246
C  -3.68884 -0.74620 -2.15215
H  -3.06834 -1.64925 -2.19251
H   0.27520  0.75295  0.76898
Zn  0.21238 -0.70142  2.35269
Zn -0.78156  1.30312 -1.77703
P   2.06197 -1.01714 -0.63786
C   0.35231 -0.64602  4.31973
H  -0.48649 -0.07877  4.76065
H   0.32695 -1.67491  4.72155
H   1.29824 -0.17424  4.63152
C  -1.39810  2.28195 -3.39086
H  -0.58903  2.30538 -4.14334
H  -2.26341  1.75646 -3.83042
H  -1.68344  3.31710 -3.14710
C   3.16515  0.39244 -1.23883
C   2.64605  1.35131 -2.13377
H   1.59338  1.29670 -2.43672
C   3.46736  2.35675 -2.67289
H   3.04292  3.07969 -3.37704
C   4.82392  2.42369 -2.31556
H   5.46503  3.20850 -2.73031
C   5.35251  1.47348 -1.42711
H   6.40967  1.51360 -1.14390
C   4.53273  0.46370 -0.89611
H   4.96304 -0.27370 -0.21301
C   2.40444 -2.28593 -1.99722
C   1.72355 -3.52536 -1.98151
H   1.00808 -3.74974 -1.18298
C   1.95050 -4.48829 -2.97700
H   1.41364 -5.44179 -2.93720
C   2.85332 -4.22480 -4.01997
H   3.02325 -4.96996 -4.80397
C   3.53372 -2.99827 -4.04937
H   4.23968 -2.77986 -4.85768
C   3.31871 -2.04013 -3.04419
H   3.86654 -1.09517 -3.08259
C   3.11951 -1.68857  0.76987
C   3.67706 -2.98277  0.76441
H   3.56077 -3.62859 -0.11065
C   4.38702 -3.45606  1.88315
H   4.80550 -4.46817  1.86584
C   4.56489 -2.63814  3.00871
H   5.12057 -3.00822  3.87663
C   4.03109 -1.33768  3.01277
H   4.17472 -0.67886  3.87499
C   3.30710 -0.86773  1.90646
H   2.91611  0.15712  1.92056
Zn  0.90626  2.61650  0.46626
H  -2.01700  3.70237  0.40312
H  -3.09402  5.64279 -0.68417
C  -1.44462  4.27344 -0.33815
C  -2.06536  5.37515 -0.95292
C  -0.11577  3.89224 -0.65448
C  -1.36930  6.13275 -1.90866
H  -1.85077  6.98984 -2.39226
C   0.56512  4.68482 -1.61364
C  -0.04798  5.78515 -2.23611
H   1.60011  4.44254 -1.88493
H   0.50607  6.37451 -2.97620
H   0.84984  2.21863  3.54252
H   2.48899  2.51320  5.36435
C   1.87906  2.49448  3.27902
C   2.80700  2.65617  4.32483
C   2.24164  2.67146  1.92064
C   4.13696  3.00235  4.03567
H   4.86384  3.12927  4.84576
C   3.58780  3.03321  1.66183
C   4.52480  3.19187  2.69888
H   3.92308  3.19077  0.62951
H   5.55929  3.46857  2.46289
```

Int1Me/Ph

```
BP86 energy =      -3258.50887320
Enthalpy 0K =      -3257.644036
Enthalpy 298K =    -3257.572838
Free energy 298K = -3257.762203
Low Freq. = 10.8804 cm-1

Ru -0.18679 -0.66727 -0.03660
H  -0.73529 -1.97575 -0.95875
Zn -0.31690 -1.02468 -2.57969
Zn -0.44794 -3.03192  0.79120
P  -2.50668 -0.25328  0.21460
C  -0.30535 -1.78725 -4.39494
H   0.73042 -1.97540 -4.72640
H  -0.77918 -1.08956 -5.10538
H  -0.85012 -2.74783 -4.42303
C  -0.62599 -4.96348  1.11623
H   0.23487 -5.51347  0.69798
H  -1.54993 -5.35021  0.65158
H  -0.67263 -5.16947  2.19963
C  -3.57031 -1.69538  0.84323
C  -3.29751 -2.18958  2.13928
H  -2.50862 -1.73057  2.74815
C  -4.04221 -3.24574  2.68406
H  -3.81221 -3.60468  3.69256
C  -5.07541 -3.83763  1.93759
H  -5.65212 -4.66880  2.35624
C  -5.36681 -3.34717  0.65731
H  -6.17856 -3.78959  0.06978
C  -4.62800 -2.27815  0.11691
H  -4.89183 -1.89808 -0.87326
C  -3.14070  1.05004  1.41676
C  -2.25921  1.90694  2.09983
H  -1.17989  1.82459  1.93515
C  -2.73998  2.86412  3.01095
H  -2.02886  3.51474  3.52966
C  -4.11759  2.97306  3.24832
H  -4.49718  3.71563  3.95792
C  -5.00995  2.11821  2.57675
H  -6.08683  2.19204  2.76225
C  -4.52691  1.16124  1.67291
H  -5.23062  0.49126  1.16809
C  -3.41270  0.20729 -1.36917
C  -3.99539  1.47573 -1.56563
H  -3.98594  2.21342 -0.75828
C  -4.59162  1.80724 -2.79532
H  -5.02824  2.80242 -2.92989
```

```
C   -4.62871  0.87417 -3.84129
H   -5.09651  1.13348 -4.79638
C   -4.05059 -0.39377 -3.65838
H   -4.06644 -1.13094 -4.46770
C   -3.43845 -0.71923 -2.43864
H   -2.98510 -1.71005 -2.31688
H    0.28747  0.42663  1.18357
Zn  -0.03974 -1.03493  2.46738
Zn  -0.30699  1.41129 -1.85846
P    2.11249 -1.15249 -0.33558
C   -0.07968 -1.12304  4.43541
H   -0.88061 -0.48165  4.84377
H   -0.26248 -2.16083  4.76622
H    0.88417 -0.79374  4.85783
C   -0.73175  1.82341 -3.77692
H    0.14932  1.64739 -4.41985
H   -1.57172  1.23285 -4.17655
H   -0.98852  2.89533 -3.82368
C    3.22115  0.19013 -1.07717
C    2.91544  0.64434 -2.38039
H    2.04802  0.23666 -2.91429
C    3.71932  1.59922 -3.02007
H    3.46649  1.92505 -4.03436
C    4.83987  2.13277 -2.35884
H    5.46469  2.88453 -2.85216
C    5.15224  1.69203 -1.06502
H    6.02319  2.09808 -0.53990
C    4.35694  0.71954 -0.43095
H    4.63197  0.37474  0.56905
C    2.64760 -2.58049 -1.44724
C    1.74852 -3.59338 -1.83265
H    0.70131 -3.54277 -1.51906
C    2.17384 -4.67207 -2.62779
H    1.45486 -5.44509 -2.91761
C    3.50827 -4.74936 -3.05197
H    3.84054 -5.58546 -3.67609
C    4.41563 -3.74446 -2.67516
H    5.45938 -3.79415 -3.00312
C    3.99015 -2.66994 -1.88052
H    4.70707 -1.89310 -1.59684
C    3.02659 -1.58947  1.24982
C    3.54116 -2.88163  1.48550
H    3.47983 -3.64770  0.70730
C    4.13386 -3.19780  2.72045
H    4.51910 -4.20927  2.88853
C    4.23916 -2.22659  3.72800
H    4.70867 -2.47416  4.68569
C    3.73465 -0.93543  3.49988
H    3.80846 -0.16286  4.27235
C    3.12073 -0.62160  2.27680
H    2.72460  0.38947  2.12662
Zn   0.70120  2.03497  0.36571
H   -1.84002  3.72075 -0.19140
H   -2.11355  6.09077 -0.85280
C   -1.03065  4.21160 -0.74745
C   -1.19218  5.55805 -1.11609
C    0.14578  3.48361 -1.06890
C   -0.17183  6.22213 -1.81666
H   -0.29373  7.27170 -2.10592
C    1.16207  4.18432 -1.77111
C    1.00806  5.53081 -2.14112
H    2.08933  3.66568 -2.04386
H    1.81046  6.04328 -2.68502
H    0.83850  1.52319  3.46516
H    1.93320  2.66267  5.36587
C    1.46844  2.40080  3.25756
C    2.09043  3.03829  4.34748
C    1.63033  2.85667  1.92674
C    2.91208  4.15555  4.12730
H    3.40419  4.65411  4.97008
C    2.45098  3.99603  1.74074
C    3.09221  4.63273  2.81838
H    2.58685  4.40497  0.73135
H    3.72633  5.50921  2.63790
```

TS2Me/Ph

```
BP86 energy =       -3258.49867021
Enthalpy 0K =       -3257.634078
Enthalpy 298K =     -3257.563740
Free energy 298K = -3257.751002
Low Freq. =  -36.1058 cm-1
Second Freq. = 9.2010 cm-1

Ru   0.08175 -0.67398 -0.08600
H   -0.04287 -1.73856 -1.39085
Zn   0.07100 -0.04900 -2.53019
Zn   0.52728 -3.12377  0.14933
P   -2.21888 -1.13953  0.15865
C    0.61619 -0.29035 -4.42168
H    0.72728  0.67805 -4.93826
H   -0.14602 -0.87524 -4.96782
H    1.57434 -0.83488 -4.48363
C    0.92192 -5.04814  0.07010
H    1.84536 -5.23201 -0.50498
H    0.08888 -5.58807 -0.41255
H    1.05455 -5.45582  1.08723
C   -2.68556 -2.96997  0.36809
C   -2.27716 -3.60611  1.56382
H   -1.74186 -3.03616  2.33286
C   -2.56996 -4.95637  1.80249
H   -2.24585 -5.42186  2.73901
C   -3.27328 -5.70521  0.84349
H   -3.49625 -6.76189  1.02342
C   -3.69475 -5.08388 -0.34002
H   -4.25586 -5.65142 -1.09025
C   -3.41252 -3.72491 -0.57335
H   -3.77740 -3.25853 -1.49183
C   -3.18114 -0.49716  1.64438
C   -2.59728  0.39417  2.56345
H   -1.58069  0.75902  2.38822
C   -3.30823  0.82232  3.69928
H   -2.83413  1.51281  4.40414
C   -4.61615  0.36897  3.92238
H   -5.17076  0.70090  4.80629
C   -5.21150 -0.51835  3.00827
H   -6.23146 -0.87982  3.17664
C   -4.49811 -0.95390  1.88270
H   -4.96395 -1.66151  1.18902
C   -3.32036 -0.65408 -1.28624
C   -4.36852  0.28346 -1.16911
H   -4.58413  0.74907 -0.20356
C   -5.15620  0.61939 -2.28642
```

H	-5.95673	1.35814	-2.17537
C	-4.92457	0.00965	-3.52707
H	-5.54656	0.26326	-4.39141
C	-3.87408	-0.91527	-3.65914
H	-3.67177	-1.38523	-4.62725
C	-3.06703	-1.22787	-2.55596
H	-2.23163	-1.92621	-2.68097
H	0.12826	0.28126	1.32358
Zn	0.40871	-1.44819	2.27980
Zn	-1.64591	2.04871	-1.84934
P	2.45582	-0.48745	-0.23856
C	0.47902	-1.92194	4.18601
H	-0.15916	-2.79517	4.40663
H	1.51684	-2.16553	4.46872
H	0.13541	-1.07641	4.80691
C	-1.80213	1.98484	-3.82765
H	-0.94692	2.51490	-4.28154
H	-1.88786	0.99879	-4.30350
H	-2.72101	2.56074	-4.04343
C	3.33450	1.09197	-0.81690
C	2.93584	1.66624	-2.04278
H	2.10709	1.22965	-2.61407
C	3.60712	2.77812	-2.57372
H	3.28169	3.19855	-3.53109
C	4.69189	3.34156	-1.88211
H	5.21431	4.21295	-2.29044
C	5.10132	2.77705	-0.66557
H	5.94453	3.20737	-0.11499
C	4.43425	1.65768	-0.13936
H	4.77529	1.23394	0.80880
C	3.41636	-1.68320	-1.34056
C	2.75914	-2.56393	-2.21926
H	1.66583	-2.57076	-2.26109
C	3.49150	-3.41974	-3.06173
H	2.95891	-4.09399	-3.74029
C	4.89290	-3.40402	-3.03405
H	5.46488	-4.06992	-3.68862
C	5.56083	-2.52086	-2.16759
H	6.65547	-2.49416	-2.14529
C	4.82989	-1.66329	-1.33379
H	5.36329	-0.96782	-0.67803
C	3.26566	-0.76800	1.43518
C	3.90387	-1.98340	1.76341
H	4.00546	-2.77012	1.00963
C	4.41749	-2.19238	3.05492
H	4.90624	-3.14326	3.29248
C	4.31230	-1.18917	4.03120
H	4.72175	-1.35060	5.03374
C	3.67100	0.02064	3.71577
H	3.57666	0.80937	4.46930
C	3.14011	0.22878	2.43267
H	2.63507	1.17556	2.20694
Zn	0.18351	1.87213	0.11753
H	-3.23124	2.35588	0.96176
H	-4.00996	4.34089	2.20925
C	-2.91773	3.34736	0.61791
C	-3.36220	4.47268	1.33450
C	-2.08326	3.47031	-0.51801
C	-2.97596	5.76127	0.93284
H	-3.31669	6.63992	1.49160
C	-1.71083	4.78501	-0.90196
C	-2.14769	5.91523	-0.19129
H	-1.06336	4.93806	-1.77542
H	-1.83976	6.91674	-0.51347
H	0.38139	2.57068	3.03324
H	1.43528	4.34593	4.40086
C	0.94499	3.36477	2.52229
C	1.54156	4.36773	3.30933
C	1.04964	3.35672	1.10942
C	2.26902	5.39795	2.69332
H	2.73692	6.18260	3.29870
C	1.78870	4.40854	0.51863
C	2.39167	5.41452	1.29375
H	1.90890	4.44563	-0.57082
H	2.96005	6.21498	0.80459

TS1′Me/Ph

BP86 energy = -3258.49770145
Enthalpy 0K = -3257.632820
Enthalpy 298K = -3257.562695
Free energy 298K = -3257.746844
Low Freq. = -29.7700 cm-1
Second Freq. = 12.6260 cm-1

Ru	-0.51411	0.40791	0.12744
H	-0.06625	0.33143	1.78422
Zn	-1.67316	1.74200	1.90206
P	-2.37026	-1.08013	0.09196
C	-2.43994	2.61148	3.49739
H	-2.31708	3.70689	3.43401
H	-1.94914	2.26148	4.42330
H	-3.51892	2.39167	3.57643
C	-3.11051	-1.78774	1.68124
C	-2.76345	-1.24932	2.93525
H	-2.02265	-0.44497	2.99769
C	-3.33592	-1.74236	4.12001
H	-3.04521	-1.30774	5.08175
C	-4.26321	-2.79323	4.06615
H	-4.70695	-3.18492	4.98735
C	-4.61094	-3.34695	2.82269
H	-5.32679	-4.17413	2.76964
C	-4.03971	-2.85064	1.64119
H	-4.31389	-3.30306	0.68383
C	-3.89742	-0.27906	-0.67103
C	-3.89811	0.02077	-2.05422
H	-3.03123	-0.24498	-2.66903
C	-5.00980	0.63394	-2.65166
H	-4.99720	0.84696	-3.72570
C	-6.12950	0.97812	-1.87511
H	-6.99482	1.46088	-2.34072
C	-6.12901	0.70335	-0.49898
H	-6.99464	0.97145	0.11599
C	-5.02436	0.07229	0.09966
H	-5.04707	-0.15488	1.16973
C	-2.29646	-2.65704	-0.95985
C	-3.41037	-3.12074	-1.69611
H	-4.34435	-2.55204	-1.69609
C	-3.33376	-4.30625	-2.44603
H	-4.20823	-4.64064	-3.01467
C	-2.14507	-5.05171	-2.47510
H	-2.08309	-5.96898	-3.06988
C	-1.03418	-4.60812	-1.74190

```
H  -0.09159 -5.16369 -1.76306
C  -1.11353 -3.42343 -0.99200
H  -0.23388 -3.09841 -0.42407
H  -0.97567  0.65788 -1.46181
Zn -2.14088  2.19678 -0.63527
Zn  0.47799 -0.89524 -1.80473
P   1.11146  2.08389 -0.33176
C  -3.34743  3.61951 -1.24911
H  -4.38151  3.24158 -1.30493
H  -3.31650  4.46558 -0.54082
H  -3.04225  3.98535 -2.24381
C   0.58810 -1.33884 -3.72948
H   0.80919 -2.41044 -3.85798
H  -0.36606 -1.10763 -4.23657
H   1.39448 -0.76302 -4.21617
C   2.79990  1.64831 -1.08263
C   3.78937  1.13449 -0.20861
H   3.59181  1.06283  0.86484
C   5.06399  0.78344 -0.68769
H   5.80919  0.39143  0.01188
C   5.37778  0.94860 -2.04628
H   6.37149  0.68151 -2.41966
C   4.40780  1.46681 -2.91847
H   4.64299  1.61326 -3.97818
C   3.12937  1.81096 -2.44466
H   2.40339  2.23489 -3.14256
C   1.76551  3.22847  1.01773
C   1.28793  3.15324  2.33788
H   0.53500  2.40138  2.59818
C   1.78065  4.01118  3.33728
H   1.39336  3.93080  4.35807
C   2.76673  4.95737  3.02450
H   3.15382  5.62688  3.79966
C   3.26431  5.03490  1.71180
H   4.04172  5.76412  1.46030
C   2.77363  4.17477  0.71902
H   3.17900  4.23695 -0.29609
C   0.48129  3.32476 -1.61046
C   0.34847  4.70222 -1.33572
H   0.62894  5.09513 -0.35491
C  -0.14979  5.58388 -2.31062
H  -0.25370  6.64717 -2.06999
C  -0.51143  5.10995 -3.58001
H  -0.89200  5.80029 -4.33982
C  -0.39445  3.73908 -3.86384
H  -0.68337  3.35156 -4.84650
C   0.08306  2.85328 -2.88584
H   0.13399  1.78330 -3.11534
H   4.07231 -5.55242 -3.76833
C   3.75929 -4.74818 -3.09325
C   3.05225 -5.04558 -1.91705
C   4.06655 -3.41058 -3.39359
H   4.62079 -3.16944 -4.30861
C   3.66483 -2.37853 -2.52729
C   2.94792 -2.64610 -1.33690
H   3.91677 -1.34529 -2.78942
C   3.11607 -1.31212  2.03304
C   2.91615 -0.29094  2.99242
H   2.29781  0.58055  2.74017
C   3.92091 -2.41216  2.42672
C   3.48844 -0.35451  4.27607
H   4.10951 -3.22928  1.71843
C   4.49779 -2.48567  3.70699
H   3.31461  0.45655  4.99315
C   4.28146 -1.45554  4.63742
H   5.11764 -3.34840  3.97848
H   4.72847 -1.51061  5.63622
H   0.53424 -2.97405  3.64544
Zn  2.50760 -1.36488  0.11928
Zn  0.33166 -1.52678  1.55519
C   0.25686 -3.19695  2.60044
H  -0.75031 -3.64437  2.58287
C   2.65337 -4.00620 -1.05631
H   0.98343 -3.92915  2.20916
H   2.81873 -6.08690 -1.66375
H   2.10961 -4.27115 -0.13872
```

Int1’Me/Ph

```
BP86 energy =      -3258.50645993
Enthalpy 0K =      -3257.641093
Enthalpy 298K =    -3257.570074
Free energy 298K = -3257.757329
Low Freq. = 9.6870 cm-1

Ru  0.58352  0.37763  0.16714
H   0.97864  0.56820  1.78550
Zn  2.64787  1.52650  1.03678
P  -0.71044  2.36847  0.24552
C   4.17494  2.27575  2.02517
H   5.11658  1.80690  1.69116
H   4.07304  2.11056  3.11248
H   4.24876  3.36292  1.84697
C  -0.58456  3.51031  1.74607
C   0.28097  3.23473  2.82096
H   0.89520  2.32976  2.80445
C   0.35217  4.09505  3.93111
H   1.03070  3.85573  4.75631
C  -0.44537  5.24677  3.98042
H  -0.39294  5.91753  4.84430
C  -1.32059  5.52989  2.91767
H  -1.95497  6.42205  2.94972
C  -1.39398  4.66774  1.81451
H  -2.09297  4.89343  1.00302
C  -0.29525  3.54943 -1.16049
C  -0.59467  3.15926 -2.48801
H  -1.09434  2.20263 -2.67753
C  -0.26611  3.98820 -3.57157
H  -0.51494  3.67001 -4.58919
C   0.38519  5.21359 -3.35051
H   0.64641  5.85836 -4.19583
C   0.70655  5.59995 -2.04039
H   1.22261  6.54847 -1.85752
C   0.36529  4.77839 -0.95201
H   0.61307  5.10055  0.06351
C  -2.60137  2.34339  0.13695
C  -3.33995  3.16213 -0.74430
H  -2.82328  3.82824 -1.44073
C  -4.74623  3.13288 -0.73907
H  -5.29980  3.77102 -1.43648
C  -5.43562  2.29798  0.15302
H  -6.53029  2.27491  0.15383
C  -4.71115  1.49227  1.04688
```

H	-5.22979	0.83056	1.74715
C	-3.30846	1.51572	1.03453
H	-2.76653	0.87755	1.74334
H	0.48555	0.48663	-1.53567
Zn	2.07337	1.63055	-1.50671
Zn	-0.57560	-0.83674	-2.21585
P	1.94178	-1.56425	0.08386
C	3.12603	2.51220	-2.91079
H	2.84368	3.57551	-2.98507
H	4.20149	2.43948	-2.67544
H	2.94763	2.03102	-3.88815
C	0.15782	-1.07838	-4.04725
H	-0.67407	-0.98506	-4.76753
H	0.93328	-0.34035	-4.31761
H	0.58320	-2.09134	-4.16093
C	1.25120	-3.14620	-0.69004
C	0.10341	-3.71068	-0.09105
H	-0.35040	-3.22851	0.78214
C	-0.45412	-4.90475	-0.57219
H	-1.34303	-5.31529	-0.08267
C	0.12909	-5.55645	-1.67259
H	-0.30472	-6.48553	-2.05667
C	1.27401	-5.01038	-2.27229
H	1.74083	-5.51366	-3.12574
C	1.83852	-3.81884	-1.78169
H	2.74218	-3.42225	-2.25176
C	2.58700	-2.31210	1.69341
C	2.62642	-1.57052	2.89036
H	2.24244	-0.54638	2.90913
C	3.14405	-2.13224	4.07070
H	3.15867	-1.53722	4.98961
C	3.62870	-3.44827	4.07046
H	4.02546	-3.88999	4.99047
C	3.59781	-4.19780	2.88247
H	3.97249	-5.22683	2.87081
C	3.08304	-3.63570	1.70474
H	3.06417	-4.23283	0.78784
C	3.55044	-1.35965	-0.87256
C	4.81377	-1.43313	-0.24988
H	4.88732	-1.67859	0.81337
C	5.98706	-1.19272	-0.98635
H	6.95850	-1.24622	-0.48329
C	5.91767	-0.89483	-2.35564
H	6.83326	-0.71520	-2.92848
C	4.66449	-0.82528	-2.98676
H	4.59568	-0.59267	-4.05435
C	3.48905	-1.04141	-2.24994
H	2.51835	-0.97986	-2.75598
H	-5.97738	-2.16568	-3.87704
C	-5.04039	-1.96130	-3.34734
C	-4.74140	-0.66107	-2.91425
C	-4.12816	-3.00269	-3.09480
H	-4.35589	-4.02171	-3.42898
C	-2.92923	-2.74103	-2.41607
C	-2.59376	-1.43508	-1.96719
H	-2.23668	-3.57010	-2.22933
C	-3.01991	-2.22829	1.18878
C	-2.65997	-3.05952	2.27719
H	-1.61058	-3.12591	2.59914
C	-4.39653	-2.18521	0.85650
C	-3.60909	-3.81523	2.99075
H	-4.72967	-1.57275	0.00938
C	-5.35828	-2.92157	1.57183
H	-3.28710	-4.45141	3.82395
C	-4.96678	-3.74325	2.64178
H	-6.41563	-2.86228	1.28556
H	-5.71258	-4.32288	3.19743
H	-0.73378	-1.41378	4.62574
Zn	-1.69345	-1.18355	0.07059
Zn	-0.62089	-0.36285	2.28630
C	-1.15700	-0.49837	4.17650
H	-0.79177	0.37220	4.74956
C	-3.53443	-0.40471	-2.23571
H	-2.25429	-0.54993	4.26900
H	-5.44597	0.15790	-3.09953
H	-3.33396	0.62160	-1.90385

TS2′Me/Ph

BP86 energy = -3258.50495663
Enthalpy 0K = -3257.640761
Enthalpy 298K = -3257.570206
Free energy 298K = -3257.757134
Low Freq. = -32.4064 cm-1
Second Freq. = 9.8400 cm-1

Ru	0.48630	0.47004	0.16034
H	1.06308	0.87490	1.68777
Zn	2.35379	2.10593	0.59204
P	-1.14111	2.18468	0.31201
C	3.79907	3.24964	1.28230
H	4.73432	3.05312	0.73058
H	3.98483	3.06520	2.35558
H	3.53757	4.31500	1.15677
C	-1.05219	3.42698	1.73625
C	-0.03138	3.37903	2.70402
H	0.73557	2.60148	2.64390
C	0.00792	4.30590	3.76101
H	0.81120	4.24448	4.50248
C	-0.97740	5.29749	3.86421
H	-0.94989	6.01960	4.68688
C	-2.00657	5.35310	2.90861
H	-2.78661	6.11821	2.98351
C	-2.04685	4.42451	1.85892
H	-2.86493	4.47225	1.13346
C	-1.14555	3.33369	-1.18008
C	-1.55786	2.82286	-2.43420
H	-1.90642	1.78736	-2.51431
C	-1.53687	3.63065	-3.58152
H	-1.87022	3.21715	-4.53896
C	-1.08128	4.95746	-3.50180
H	-1.05936	5.58681	-4.39733
C	-0.64539	5.46629	-2.26862
H	-0.27861	6.49577	-2.19713
C	-0.68195	4.66455	-1.11465
H	-0.35157	5.08188	-0.15892
C	-2.99218	1.80243	0.46237
C	-3.98303	2.45263	-0.30454
H	-3.69818	3.18839	-1.06183
C	-5.34528	2.16340	-0.10835
H	-6.09714	2.67541	-0.71884
C	-5.74022	1.23108	0.86298
H	-6.80111	1.00510	1.01250

```
C  -4.76439  0.59052  1.64377
H  -5.05261 -0.14167  2.40414
C  -3.40534  0.87485  1.44166
H  -2.66258  0.36496  2.06701
H   0.07521  0.28458 -1.48639
Zn  1.40371  1.76561 -1.82770
Zn -0.56016 -1.22274 -2.55039
P   2.20326 -1.17745  0.09686
C   2.07470  2.68354 -3.43425
H   1.50806  3.61539 -3.59741
H   3.14089  2.93634 -3.30121
H   1.97431  2.05121 -4.33366
C   0.73618 -1.18544 -4.03693
H   0.23439 -1.52572 -4.96014
H   1.13719 -0.17442 -4.22307
H   1.58134 -1.86710 -3.84062
C   1.98784 -2.78811 -0.86769
C   0.80712 -3.54289 -0.69378
H   0.03214 -3.21487  0.00945
C   0.61516 -4.75901 -1.37084
H  -0.30669 -5.32479 -1.20207
C   1.60334 -5.24094 -2.24410
H   1.45401 -6.18325 -2.78132
C   2.78953 -4.51014 -2.41230
H   3.57411 -4.88115 -3.08036
C   2.98534 -3.29994 -1.72493
H   3.92282 -2.75533 -1.86209
C   2.75594 -1.86063  1.76830
C   2.97792 -0.97338  2.84641
H   2.81843  0.10124  2.71078
C   3.40830 -1.44843  4.09536
H   3.57632 -0.74015  4.91332
C   3.61074 -2.82369  4.29540
H   3.93363 -3.19706  5.27268
C   3.39566 -3.71415  3.23299
H   3.55229 -4.78872  3.37513
C   2.98151 -3.23764  1.97738
H   2.83283 -3.94696  1.15902
C   3.85832 -0.59483 -0.60291
C   5.03669 -0.51775  0.16661
H   5.03643 -0.85677  1.20618
C   6.22143 -0.00143 -0.39005
H   7.12346  0.06016  0.22812
C   6.25148  0.42495 -1.72554
H   7.17497  0.82486 -2.15685
C   5.08824  0.33032 -2.50949
H   5.09918  0.64742 -3.55730
C   3.90203 -0.16825 -1.95139
H   3.00875 -0.24945 -2.58200
H  -6.17821 -2.90172 -2.88666
C  -5.14217 -2.60282 -2.69260
C  -4.84284 -1.28126 -2.32815
C  -4.10200 -3.54311 -2.80110
H  -4.32764 -4.57888 -3.08042
C  -2.77528 -3.15633 -2.55622
C  -2.43493 -1.82470 -2.19653
H  -1.98378 -3.90937 -2.65638
C  -2.41782 -2.78505  1.15142
C  -1.78571 -3.56078  2.15486
H  -0.72700 -3.38786  2.39873
C  -3.78568 -3.05175  0.90499
C  -2.47302 -4.55842  2.87149
H  -4.31718 -2.48429  0.13266
C  -4.48605 -4.03795  1.62271
H  -1.94966 -5.14205  3.63838
C  -3.83081 -4.79688  2.60732
H  -5.54654 -4.21968  1.40872
H  -4.37510 -5.56781  3.16465
H  -0.02457 -1.53226  4.70062
Zn -1.35871 -1.34788  0.23629
Zn -0.22700 -0.36588  2.43177
C  -0.66031 -0.71661  4.31588
H  -0.48551  0.18454  4.92978
C  -3.50797 -0.90347 -2.08504
H  -1.71475 -1.02049  4.42342
H  -5.64365 -0.53982 -2.22918
H  -3.31252  0.13679 -1.79764
```

Int4Me/Ph

```
BP86 energy =      -2951.46581942
Enthalpy 0K =      -2950.671615
Enthalpy 298K =    -2950.607763
Free energy 298K = -2950.782916
Low Freq. = 7.3814 cm-1

Ru -0.00006 -0.55541  0.00003
H   0.22706 -0.53271 -1.68054
Zn  0.52307  1.54691 -1.22880
Zn  0.32971 -2.62025 -1.34746
P  -2.30493 -0.60028 -0.50653
C   0.74403 -4.08474 -2.59608
H   1.81232 -4.07265 -2.87455
H   0.14249 -3.98049 -3.51598
H   0.51368 -5.06587 -2.14507
C  -2.97882  0.95769 -1.32091
C  -3.99788  1.73959 -0.74055
H  -4.47278  1.41920  0.19067
C  -4.40485  2.94323 -1.34352
H  -5.18772  3.54396 -0.86861
C  -3.81315  3.37443 -2.53954
H  -4.13250  4.31166 -3.00678
C  -2.79922  2.60081 -3.13018
H  -2.31901  2.93082 -4.05663
C  -2.37858  1.40942 -2.51988
H  -1.57401  0.82553 -2.98183
C  -2.89797 -1.94196 -1.70123
C  -2.79131 -3.28815 -1.28198
H  -2.39812 -3.52115 -0.28470
C  -3.20413 -4.33768 -2.11552
H  -3.11752 -5.37170 -1.76601
C  -3.72121 -4.06110 -3.39293
H  -4.03470 -4.87942 -4.04933
C  -3.83803 -2.72983 -3.81671
H  -4.24841 -2.50112 -4.80616
C  -3.43876 -1.67559 -2.97452
H  -3.56021 -0.64378 -3.31523
C  -3.56388 -0.88345  0.86623
C  -3.19855 -0.77077  2.22139
H  -2.16205 -0.53286  2.48136
C  -4.14450 -0.97910  3.24092
H  -3.83755 -0.89427  4.28855
C  -5.46983 -1.30272  2.91518
```

H	-6.20676	-1.46996	3.70766
C	-5.84589	-1.41884	1.56579
H	-6.87757	-1.67521	1.30236
C	-4.90114	-1.21378	0.54940
H	-5.20370	-1.31560	-0.49784
H	-0.22728	-0.53274	1.68056
Zn	-0.32987	-2.62055	1.34702
Zn	-0.52291	1.54688	1.22893
P	2.30482	-0.60043	0.50669
C	-0.74415	-4.08520	2.59547
H	-1.81220	-4.07247	2.87488
H	-0.51488	-5.06635	2.14394
H	-0.14173	-3.98167	3.51487
C	3.56383	-0.88335	-0.86605
C	3.19853	-0.77047	-2.22121
H	2.16204	-0.53255	-2.48118
C	4.14451	-0.97862	-3.24075
H	3.83758	-0.89364	-4.28837
C	5.46984	-1.30227	-2.91502
H	6.20680	-1.46937	-3.70750
C	5.84587	-1.41858	-1.56564
H	6.87754	-1.67498	-1.30222
C	4.90109	-1.21370	-0.54924
H	5.20361	-1.31568	0.49799
C	2.89777	-1.94239	1.70110
C	3.43843	-1.67635	2.97451
H	3.55982	-0.64462	3.31551
C	3.83766	-2.73079	3.81646
H	4.24796	-2.50233	4.80601
C	3.72093	-4.06195	3.39232
H	4.03439	-4.88044	4.04853
C	3.20396	-4.33821	2.11479
H	3.11741	-5.37214	1.76501
C	2.79118	-3.28848	1.28150
H	2.39805	-3.52122	0.28413
C	2.97867	0.95735	1.32144
C	3.99778	1.73937	0.74134
H	4.47276	1.41917	-0.18990
C	4.40470	2.94288	1.34459
H	5.18762	3.54371	0.86989
C	3.81288	3.37384	2.54065
H	4.13219	4.31098	3.00810
C	2.79892	2.60011	3.13104
H	2.31862	2.92992	4.05752
C	2.37833	1.40883	2.52047
H	1.57374	0.82484	2.98224
H	0.31744	6.29751	-3.14199
H	-0.42762	4.40794	-1.72418
C	0.85821	5.34424	-3.18102
C	0.44350	4.26801	-2.37704
C	1.96534	5.19918	-4.03302
H	2.29142	6.03561	-4.66156
C	1.11641	3.02067	-2.39660
C	2.65310	3.97559	-4.07199
C	2.23252	2.90551	-3.26205
H	3.51998	3.85473	-4.73284
H	2.79547	1.96431	-3.30766
H	-0.31763	6.29838	3.13991
H	0.42724	4.40841	1.72254
C	-0.85806	5.34494	3.17969
C	-0.44344	4.26848	2.37599
H	-2.29065	6.03653	4.66073
C	-1.96464	5.19991	4.03241
C	-1.11590	3.02091	2.39655
C	-2.65195	3.97609	4.07237
C	-2.23146	2.90577	3.26270
H	-3.51841	3.85526	4.73378
H	-2.79406	1.96439	3.30910

Int6Me/Ph

BP86 energy = -3143.19579182
Enthalpy 0K = -3142.348876
Enthalpy 298K = -3142.281865
Free energy 298K = -3142.466122
Low Freq. = 8.8369 cm-1

Ru	0.15132	0.13094	-0.25373
H	-0.44103	0.28428	-1.83368
Zn	-2.28166	-0.04221	-0.75932
Zn	1.52383	1.00189	-2.14381
P	0.71762	-2.07182	-0.89323
C	2.33671	1.76323	-3.76583
H	1.85106	2.71686	-4.03695
H	2.22485	1.06038	-4.60981
H	3.41432	1.95297	-3.61807
C	-0.74210	-3.19312	-1.28921
C	-0.98349	-4.39856	-0.59942
H	-0.28093	-4.74474	0.16330
C	-2.13327	-5.16033	-0.87378
H	-2.30964	-6.08613	-0.31601
C	-3.04896	-4.73925	-1.84887
H	-3.94286	-5.33487	-2.06065
C	-2.81797	-3.54073	-2.54523
H	-3.53066	-3.19150	-3.29883
C	-1.68111	-2.76938	-2.25915
H	-1.52556	-1.82611	-2.79542
C	1.78041	-2.25162	-2.44688
C	3.12132	-1.80671	-2.37821
H	3.52277	-1.41489	-1.43531
C	3.96271	-1.88554	-3.49728
H	4.99972	-1.54319	-3.41844
C	3.47574	-2.40024	-4.71141
H	4.12911	-2.45426	-5.58832
C	2.15054	-2.85125	-4.78792
H	1.76290	-3.26489	-5.72511
C	1.30950	-2.78694	-3.66159
H	0.28714	-3.16752	-3.73539
C	1.73346	-3.16013	0.25815
C	1.93116	-2.80787	1.60656
H	1.50138	-1.87616	1.98823
C	2.69052	-3.62831	2.45963
H	2.84157	-3.33097	3.50235
C	3.26062	-4.81304	1.97182
H	3.85709	-5.45001	2.63319
C	3.07101	-5.17435	0.62666
H	3.51715	-6.09513	0.23614
C	2.31670	-4.35385	-0.22463
H	2.18390	-4.63989	-1.27306
H	0.70424	-0.03247	1.34406
Zn	2.51540	0.48727	0.37752
Zn	-1.22208	-0.96441	1.51153
P	-0.35574	2.36233	0.33918

```
C  -0.87241  3.58892 -0.99409
C  -1.26919  3.15115 -2.27232
H  -1.25886  2.08099 -2.50253
C  -1.65911  4.07360 -3.25963
H  -1.95659  3.71221 -4.24949
C  -1.65705  5.44772 -2.97837
H  -1.95540  6.16788 -3.74738
C  -1.26270  5.89647 -1.70605
H  -1.25422  6.96779 -1.47882
C  -0.87132  4.97606 -0.72255
H  -0.55778  5.33812  0.26187
C   1.02665  3.38002  1.13002
C   0.98350  3.86773  2.45097
H   0.10554  3.68502  3.07645
C   2.06191  4.60332  2.97613
H   2.01255  4.96628  4.00837
C   3.18738  4.87652  2.18617
H   4.02589  5.44744  2.59783
C   3.23112  4.41423  0.85991
H   4.10072  4.62481  0.22937
C   2.16283  3.66935  0.34009
H   2.20779  3.32508 -0.70041
C  -1.74412  2.56394  1.59404
C  -2.92606  3.27827  1.31263
H  -3.04251  3.79102  0.35404
C  -3.97071  3.32823  2.25292
H  -4.88719  3.87575  2.00898
C  -3.84422  2.68097  3.49044
H  -4.65887  2.72241  4.22072
C  -2.66829  1.96930  3.78430
H  -2.55992  1.44879  4.74094
C  -1.63289  1.90237  2.83974
H  -0.72872  1.32904  3.07541
H  -7.10531 -1.72055 -1.19500
H  -4.74344 -1.82921 -0.45730
C  -6.41301 -0.91684 -1.47320
C  -5.07157 -0.96968 -1.05564
C  -6.86676  0.16515 -2.24525
H  -7.91157  0.20955 -2.57294
C  -4.14623  0.04983 -1.39217
C  -5.97287  1.19090 -2.59231
C  -4.63347  1.13113 -2.16762
H  -6.31956  2.04032 -3.19320
H  -3.96044  1.95193 -2.44710
H  -5.00648 -2.10869  4.77288
H  -3.93424 -0.86931  2.91587
C  -3.97716 -2.35109  4.48240
C  -3.36263 -1.65431  3.42720
H  -3.74931 -3.90328  5.98662
C  -3.27382 -3.35815  5.16338
C  -2.03512 -1.93888  3.01984
C  -1.95793 -3.66350  4.78021
C  -1.35218 -2.96311  3.72172
H  -1.40257 -4.45011  5.30515
H  -0.32579 -3.23024  3.43927
H   6.13737  0.35104  4.02678
C   5.95241  0.44972  2.95035
H   3.81295  0.29878  3.17400
C   4.63614  0.42013  2.45768
H   8.05849  0.62784  2.44501
C   7.03111  0.60564  2.06443
C   6.78445  0.73273  0.68797
C   5.46384  0.70270  0.20599
H   7.62116  0.85541 -0.01025
C   4.35574  0.54563  1.07476
H   5.30032  0.80792 -0.87528
```

TS7Me/Ph

```
BP86 energy =      -3641.96493303
Enthalpy 0K =      -3640.994824
Enthalpy 298K =    -3640.918309
Free energy 298K = -3641.122367
Low Freq. =  -28.1460 cm-1
Second Freq. = 8.7743 cm-1

Ru  0.25569 -0.04983  0.13825
H   1.39261  1.17803 -0.16222
Zn  2.76263 -0.17399  0.06872
Zn -0.08164  2.08103 -1.15468
P   0.22705  0.82458  2.35140
C   0.65714  2.65804  2.48211
C  -0.14340  3.57856  1.76883
H  -0.99887  3.22409  1.18128
C   0.12095  4.95519  1.81679
H  -0.51546  5.64510  1.25386
C   1.19880  5.43824  2.57852
H   1.41297  6.51152  2.61108
C   1.99123  4.53641  3.30294
H   2.82650  4.90133  3.91025
C   1.71865  3.15638  3.26355
H   2.33783  2.47319  3.85091
C  -1.37384  0.81242  3.34762
C  -2.33666 -0.19629  3.14799
H  -2.18196 -0.94927  2.36801
C  -3.50061 -0.25743  3.93218
H  -4.23289 -1.04940  3.74721
C  -3.71987  0.70257  4.93169
H  -4.62813  0.66412  5.54185
C  -2.77060  1.71781  5.14023
H  -2.93539  2.47351  5.91559
C  -1.60601  1.77238  4.35850
H  -0.87341  2.56528  4.53628
C   1.39642  0.05626  3.61786
C   0.92303 -0.59325  4.77712
H  -0.14853 -0.62033  4.99187
C   1.81844 -1.21700  5.66396
H   1.42795 -1.72610  6.55145
C   3.19913 -1.18689  5.41855
H   3.89390 -1.67137  6.11226
C   3.68442 -0.53479  4.27250
H   4.75820 -0.50472  4.06215
C   2.79003  0.07032  3.37739
H   3.19583  0.56280  2.48522
H  -1.16187 -0.97048  0.36882
Zn -2.08476  0.88821 -0.00364
Zn  1.42519 -2.11560  1.18830
P   0.35303 -1.05654 -2.01098
C   2.77524 -3.41531  1.85033
H   2.48021 -4.44315  1.57912
H   3.77303 -3.20763  1.42541
H   2.84782 -3.34400  2.94904
C   0.49748 -2.93794 -2.03179
```

C	1.71987	-3.51346	-1.61334
H	2.55875	-2.86919	-1.32581
C	1.88844	-4.90531	-1.58633
H	2.84617	-5.32649	-1.26423
C	0.83162	-5.75023	-1.96719
H	0.95892	-6.83737	-1.94050
C	-0.38380	-5.19027	-2.38595
H	-1.21300	-5.83654	-2.69192
C	-0.55098	-3.79338	-2.42489
H	-1.50155	-3.37960	-2.76978
C	1.76457	-0.63294	-3.18467
C	2.43708	0.60233	-3.09744
H	2.16676	1.32308	-2.31932
C	3.45436	0.93759	-4.00738
H	3.96306	1.90196	-3.91473
C	3.81626	0.03647	-5.01891
H	4.61434	0.29154	-5.72372
C	3.14932	-1.19599	-5.12234
H	3.42216	-1.90508	-5.91108
C	2.13064	-1.52757	-4.21645
H	1.61648	-2.48848	-4.31247
C	-1.13036	-0.75731	-3.13016
C	-1.00721	-0.19173	-4.41617
H	-0.02133	0.06251	-4.81447
C	-2.15107	0.05765	-5.19577
H	-2.03683	0.50731	-6.18794
C	-3.42659	-0.27225	-4.71314
H	-4.31365	-0.08568	-5.32750
C	-3.55771	-0.84276	-3.43527
H	-4.54172	-1.11428	-3.03983
C	-2.42042	-1.06843	-2.64397
H	-2.54839	-1.51244	-1.65006
Zn	-1.31737	-2.76415	1.12028
C	-0.39501	-3.70607	2.63217
H	-3.99578	-1.11133	0.62778
H	-6.24436	-1.70651	-0.17246
C	-4.17037	-2.14558	0.29939
C	-5.46032	-2.47273	-0.16000
C	-3.12009	-3.09545	0.33908
C	-5.73737	-3.77691	-0.60133
H	-6.73828	-4.03790	-0.96315
C	-3.43814	-4.40624	-0.10053
C	-4.72033	-4.74531	-0.56976
H	-2.66807	-5.18857	-0.08221
H	-4.92765	-5.76839	-0.90629
H	7.07939	2.56134	-0.07289
H	4.62917	2.22753	0.05201
C	6.66974	1.54446	-0.09295
C	5.27940	1.34537	-0.02147
H	8.61589	0.59115	-0.24800
C	7.53204	0.44060	-0.19122
C	4.71192	0.04680	-0.04334
C	6.99676	-0.85765	-0.21484
C	5.60595	-1.04709	-0.13914
H	7.66383	-1.72448	-0.29035
H	5.21609	-2.07272	-0.15341
H	1.72209	6.67340	-2.55366
H	1.59785	4.64945	-1.12962
C	0.99801	5.88548	-2.79309
C	0.92440	4.73183	-1.99255
H	0.19596	6.92783	-4.52244
C	0.14261	6.02927	-3.89755
C	0.00210	3.69286	-2.27220
C	-0.78317	5.01594	-4.19498
C	-0.84954	3.86449	-3.39133
H	-1.45576	5.12324	-5.05419
H	-1.58136	3.08788	-3.64626
H	-6.37782	2.75229	-2.09843
H	-4.21126	1.55283	-2.10758
C	-5.83350	2.60951	-1.15727
C	-4.60293	1.92983	-1.15470
H	-7.32259	3.63939	0.04471
C	-6.36419	3.10806	0.04349
C	-3.87052	1.72341	0.04120
C	-5.65597	2.92415	1.24198
C	-4.42582	2.24310	1.23743
H	-6.06142	3.31289	2.18372
H	-3.89768	2.11335	2.18961
H	0.26526	-3.14130	3.30951
H	0.14156	-4.61064	2.30439
H	-1.27616	-4.02902	3.22049

Int7Me/Ph

BP86 energy = -3641.96533387
Enthalpy 0K = -3640.994742
Enthalpy 298K = -3640.917509
Free energy 298K = -3641.123877
Low Freq. = 7.9378 cm-1

Ru	-0.20012	0.10669	0.09300
H	-1.39639	-1.05758	0.39170
Zn	-2.71974	0.32498	0.12145
Zn	-0.03749	-2.37926	-0.22776
P	-0.07224	0.25500	2.46681
C	-0.52232	-1.32265	3.40213
C	0.16962	-2.51016	3.07560
H	0.94837	-2.49814	2.30368
C	-0.10339	-3.71438	3.74104
H	0.44899	-4.61823	3.46559
C	-1.08233	-3.75458	4.74831
H	-1.30462	-4.69479	5.26355
C	-1.76493	-2.57954	5.09417
H	-2.52133	-2.59439	5.88618
C	-1.48196	-1.36971	4.43389
H	-2.01333	-0.46256	4.73312
C	1.58150	0.64179	3.28499
C	2.53162	1.45156	2.63101
H	2.34076	1.81907	1.61707
C	3.73968	1.80249	3.25677
H	4.45995	2.42544	2.71741
C	4.01634	1.34037	4.55241
H	4.95912	1.60541	5.04208
C	3.07905	0.52971	5.21543
H	3.28788	0.16116	6.22540
C	1.87044	0.18472	4.59056
H	1.14945	-0.44375	5.12155
C	-1.16180	1.53769	3.31643
C	-0.61443	2.64652	3.99526
H	0.47006	2.75309	4.08592
C	-1.45287	3.62436	4.55927
H	-1.00718	4.48220	5.07389
C	-2.84748	3.50091	4.47169

```
H  -3.49787  4.26107  4.91641
C  -3.40475  2.39737  3.80382
H  -4.49068  2.28878  3.72008
C  -2.56916  1.43282  3.22214
H  -3.03035  0.58709  2.69750
H   1.30818  0.86808 -0.13253
Zn  2.08518 -0.96164  0.28886
Zn -1.35323  2.50467  0.21846
P  -0.39696  0.14286 -2.27298
C  -2.89816  3.75668  0.38562
H  -2.81392  4.54421 -0.38339
H  -3.87963  3.26380  0.28774
H  -2.85345  4.23179  1.38125
C  -0.50366  1.86099 -3.04743
C  -1.71216  2.58197 -2.89842
H  -2.56762  2.11967 -2.39312
C  -1.84360  3.87895 -3.41488
H  -2.79099  4.41408 -3.29357
C  -0.76457  4.48558 -4.08132
H  -0.86395  5.50061 -4.47953
C   0.43669  3.77941 -4.23645
H   1.28372  4.23747 -4.75732
C   0.56759  2.47301 -3.72938
H   1.50715  1.93566 -3.87857
C  -1.87081 -0.66338 -3.12097
C  -2.60305 -1.68897 -2.49160
H  -2.34307 -2.00972 -1.47799
C  -3.67296 -2.32062 -3.14891
H  -4.22904 -3.11051 -2.63506
C  -4.02681 -1.93039 -4.44826
H  -4.86486 -2.41533 -4.95941
C  -3.30187 -0.91174 -5.09026
H  -3.56968 -0.60149 -6.10583
C  -2.23216 -0.28446 -4.43458
H  -1.67436  0.50516 -4.94719
C   1.03059 -0.63987 -3.21932
C   0.83776 -1.67874 -4.15356
H  -0.17072 -2.03267 -4.38351
C   1.93977 -2.27530 -4.79237
H   1.77054 -3.08742 -5.50755
C   3.24353 -1.83240 -4.52304
H   4.09831 -2.29404 -5.02828
C   3.44460 -0.79309 -3.59856
H   4.45298 -0.43005 -3.37579
C   2.34904 -0.21186 -2.94134
H   2.53233  0.59339 -2.22089
Zn  1.17684  2.73442 -0.28418
C   0.25399  4.47332  0.32155
H   3.91990  1.06016 -0.15984
H   6.21493  1.49159 -0.93841
C   4.11402  1.99618 -0.70498
C   5.43042  2.22889 -1.14642
C   3.06325  2.91596 -0.93817
C   5.73374  3.40462 -1.85176
H   6.75504  3.59172 -2.20234
C   3.40866  4.09772 -1.64230
C   4.71633  4.34089 -2.10002
H   2.63773  4.85340 -1.84415
H   4.94342  5.26442 -2.64655
H  -6.88034 -2.27683  1.53180
H  -4.45161 -1.82629  1.36400
C  -6.53076 -1.38474  0.99859
C  -5.15294 -1.11975  0.89944
H  -8.53135 -0.70947  0.48649
C  -7.45698 -0.50701  0.41288
C  -4.66093  0.02345  0.22111
C  -6.99740  0.63262 -0.26744
C  -5.61859  0.89128 -0.35722
H  -7.71457  1.32277 -0.72735
H  -5.28937  1.79215 -0.89003
H  -2.03039 -7.03788  0.54858
H  -1.74737 -4.61564  0.98616
C  -1.31875 -6.45805 -0.05121
C  -1.15516 -5.08220  0.18832
H  -0.69304 -8.16178 -1.24541
C  -0.56941 -7.08940 -1.05719
C  -0.24612 -4.30079 -0.56775
C   0.34094 -6.33891 -1.81909
C   0.49762 -4.96344 -1.57522
H   0.93066 -6.82632 -2.60446
H   1.21473 -4.40039 -2.18561
H   6.09192 -3.87115 -1.01009
H   3.94729 -2.69448 -1.39848
C   5.65604 -3.26065 -0.21032
C   4.43849 -2.59090 -0.42278
H   7.26077 -3.67223  1.19579
C   6.31222 -3.15036  1.02630
C   3.84504 -1.79175  0.58631
C   5.74331 -2.36922  2.04530
C   4.52537 -1.70170  1.82634
H   6.24868 -2.27966  3.01428
H   4.10633 -1.09688  2.63939
H  -0.30678  4.55055  1.26725
H  -0.31932  4.98995 -0.46476
H   1.20458  5.02215  0.46369
```

TS8Me/Ph

```
BP86 energy =      -3641.95976053
Enthalpy 0K =      -3640.990082
Enthalpy 298K =    -3640.913354
Free energy 298K = -3641.118895
Low Freq. =  -32.1484 cm-1
Second Freq. = 8.2491 cm-1

Ru  0.06505  0.01600  0.12809
H  -0.23710  1.68279  0.13197
Zn  1.75784  1.91564  0.34197
Zn -2.06142  1.11857 -0.58556
P  -0.63638  0.11076  2.39092
C  -2.37804  0.78373  2.71568
C  -3.46873  0.03302  2.21918
H  -3.29669 -0.90955  1.68492
C  -4.78960  0.45744  2.42145
H  -5.61345 -0.14381  2.02426
C  -5.04777  1.64657  3.12408
H  -6.07799  1.98423  3.27608
C  -3.97599  2.39277  3.63378
H  -4.16390  3.31450  4.19500
C  -2.65014  1.96268  3.43885
H  -1.83273  2.54888  3.86677
C  -0.77503 -1.46292  3.41192
C  -0.23921 -2.68307  2.96120
```

H	0.26657	-2.72656	1.99211
C	-0.35085	-3.84936	3.73789
H	0.07610	-4.78712	3.36805
C	-1.00370	-3.80720	4.97830
H	-1.09401	-4.71483	5.58408
C	-1.54695	-2.59503	5.43789
H	-2.06414	-2.55411	6.40231
C	-1.43708	-1.43292	4.66078
H	-1.87590	-0.49777	5.02341
C	0.39403	1.21976	3.50729
C	1.08724	0.73395	4.63638
H	1.01830	-0.32274	4.90805
C	1.86579	1.60314	5.42371
H	2.40270	1.20491	6.29113
C	1.95213	2.96585	5.10557
H	2.55466	3.63939	5.72339
C	1.27311	3.45985	3.97803
H	1.34438	4.51764	3.70572
C	0.51301	2.59255	3.18024
H	0.00316	2.99064	2.29573
H	0.06458	-1.68737	0.15266
Zn	-1.84063	-1.52660	-0.38377
Zn	2.91404	0.20895	1.98794
P	0.67095	-0.09563	-2.17779
C	4.01985	1.89238	2.03342
H	4.62033	2.04028	1.11961
H	3.52338	2.84276	2.27560
H	4.71990	1.64892	2.85566
C	2.45139	-0.48973	-2.69109
C	3.49365	0.27759	-2.12796
H	3.26369	1.04936	-1.38258
C	4.82716	0.08697	-2.51892
H	5.61562	0.70055	-2.07086
C	5.14460	-0.88630	-3.48077
H	6.18534	-1.04536	-3.78133
C	4.11818	-1.65042	-4.05436
H	4.35295	-2.41178	-4.80567
C	2.78118	-1.44990	-3.67023
H	1.99748	-2.05473	-4.13424
C	0.40176	1.37302	-3.32215
C	-0.05388	2.60964	-2.83284
H	-0.25566	2.73347	-1.76563
C	-0.22121	3.70643	-3.69678
H	-0.56291	4.66256	-3.28942
C	0.06426	3.57418	-5.06222
H	-0.06462	4.42707	-5.73673
C	0.52900	2.34412	-5.56148
H	0.76352	2.23487	-6.62569
C	0.70395	1.25415	-4.69835
H	1.08263	0.30730	-5.09638
C	-0.28879	-1.45161	-3.05974
C	-1.36055	-1.15448	-3.92846
H	-1.59606	-0.11331	-4.16983
C	-2.12712	-2.18780	-4.49414
H	-2.95887	-1.93830	-5.16135
C	-1.82818	-3.52922	-4.21159
H	-2.42644	-4.33214	-4.65364
C	-0.76368	-3.83484	-3.34785
H	-0.52248	-4.87733	-3.11644
C	-0.00400	-2.80651	-2.76773
H	0.82039	-3.06463	-2.09272
Zn	2.00871	-1.59913	0.34833
C	3.34181	-1.51340	2.92991
H	1.14412	-4.49990	0.09502
H	2.26131	-6.66031	-0.37000
C	2.22793	-4.50368	-0.09048
C	2.85249	-5.73669	-0.35568
C	2.95415	-3.28746	-0.05797
C	4.23372	-5.78159	-0.60056
H	4.72684	-6.73793	-0.80784
C	4.34551	-3.36571	-0.30762
C	4.97936	-4.59105	-0.57728
H	4.95329	-2.45266	-0.29766
H	6.05891	-4.61819	-0.76807
H	0.38654	6.78725	-0.29733
H	0.06945	4.39678	0.25339
C	1.23653	6.09798	-0.36966
C	1.07039	4.73777	-0.05148
H	2.63017	7.62588	-1.03853
C	2.49261	6.56897	-0.78416
C	2.13944	3.81061	-0.12199
C	3.57072	5.67330	-0.87242
C	3.39225	4.31837	-0.54071
H	4.55460	6.03128	-1.19853
H	4.25860	3.64879	-0.61114
H	-5.56667	4.87064	-0.30000
H	-3.62628	3.55571	0.50164
C	-5.20721	4.01962	-0.89079
C	-4.10257	3.27268	-0.44541
H	-6.71402	4.25590	-2.43863
C	-5.85239	3.67557	-2.09002
C	-3.60857	2.16894	-1.18318
C	-5.38779	2.58205	-2.83833
C	-4.28019	1.84282	-2.38734
H	-5.88716	2.30597	-3.77463
H	-3.93688	0.99337	-2.99254
H	-6.59608	-3.00020	-1.57388
H	-4.70677	-1.40332	-1.43010
C	-5.63923	-3.32943	-1.15156
C	-4.56214	-2.42946	-1.06761
H	-6.32845	-5.35145	-0.75432
C	-5.49030	-4.64823	-0.69261
C	-3.31062	-2.81968	-0.52968
C	-4.26187	-5.05987	-0.15021
C	-3.18938	-4.15438	-0.07129
H	-4.13961	-6.08708	0.21351
H	-2.24175	-4.50183	0.35985
H	3.63266	-1.17098	3.94160
H	4.21856	-1.99735	2.46835
H	2.55202	-2.27237	3.04022

S-4 References

[1] R. A. Schunn, E. R. Wonchoba, G. Wilkinson, *Inorg. Synth.* **1972**, *13*, 131–134.

[2] F. M. Miloserdov, C. J. Isaac, M. L. Beck, A. L. Burnage, J. C. B Farmer, S. A Macgregor, M. F. Mahon, M. K Whittlesey, *Inorg. Chem.* **2020**, *59*, 15606–15619.

[3] F. M. Miloserdov, N. A. Rajabi, J. P. Lowe, M. F. Mahon, S. A. Macgregor, M. K. Whittlesey, *J. Am. Chem. Soc.* **2020**, *13*, 6340–6349.

[4] G. M. Sheldrick, *Acta Cryst.* **2015**, *A71*, 3-8.

[5] G. M. Sheldrick, *Acta Cryst.* **2015**, *C71*, 3-8.

[6] O. V Dolomanov, L. J. Bourhis, R. J. Gildea, J. A. K. Howard, H. Puschmann, *J. Appl. Cryst.* **2009**, *42*, 339-341.

[7] M. J. Frisch, G. W. Trucks, H. B. Schlegel, G. E. Scuseria, M. A. Robb, J. R. Cheeseman, G. Scalmani, V. Barone, G. A. Petersson, H. Nakatsuji, X. Li, M. Caricato, A. V. Marenich, J. Bloino, B. G. Janesko, R. Gomperts, B. Mennucci, H. P. Hratchian, J. V. Ortiz, A. F. Izmaylov, J. L. Sonnenberg, D. Williams-Young, F. Ding, F. Lipparini, F. Egidi, J. Goings, B. Peng, A. Petrone, T. Henderson, D. Ranasinghe, V. G. Zakrzewski, J. Gao, N. Rega, G. Zheng, W. Liang, M. Hada, M. Ehara, K. Toyota, R. Fukuda, J. Hasegawa, M. Ishida, T. Nakajima, Y. Honda, O. Kitao, H. Nakai, T. Vreven, K. Throssell, J. A. Montgomery, Jr., J. E. Peralta, F. Ogliaro, M. J. Bearpark, J. J. Heyd, E. N. Brothers, K. N. Kudin, V. N. Staroverov, T. A. Keith, R. Kobayashi, J. Normand, K. Raghavachari, A. P. Rendell, J. C. Burant, S. S. Iyengar, J. Tomasi, M. Cossi, J. M. Millam, M. Klene, C. Adamo, R. Cammi, J. W.

Ochterski, R. L. Martin, K. Morokuma, O. Farkas, J. B. Foresman, D. J. Fox, Gaussian Inc. Wallingford CT, 2016.

[8] D. Andrae, U. Häußermann, M. Dolg, H. Stoll, H. Preuß, *Theor. Chimica Acta* **1990**, *77*, 123-141.

[9] W. J. Hehre, R. Ditchfield, J. A. Pople, *J. Chem. Phys.* **1972**, *56*, 2257-2261.

[10] P. C. Hariharan, J. A. Pople, *Theor. Chim. Acta* **1973**, *28*, 213-222.

[11] A. Höllwarth, M. Böhme, S. Dapprich, A. W. Ehlers, A. Gobbi, V. Jonas, K. F. Köhler, R. Stegmann, A. Veldkamp, G. Frenking, *Chem. Phys. Lett.* **1993**, *208*, 237-240.

[12] A. D. Becke, *Phys. Rev. A* **1988**, *38*, 3098-3100.

[13] J. P. Perdew, *Phys. Rev. B* **1986**, *33*, 8822-8824.

[14] J.-D. Chai, M. Head-Gordon, *Phys. Chem. Chem. Phys.* **2008**, *10*, 6615-6620.

[15] a) F. Weigend, R. Ahlrichs, *Phys. Chem. Chem. Phys.* **2005**, *7*, 3297-3305. b) F. Weigend, *Phys. Chem. Chem. Phys.* **2006**, *8*, 1057-1065

[16] J. Tomasi, B. Mennucci, R. Cammi, *Chem. Rev.* **2005**, *105*, 2999-3093.

[17] R. F. W. Bader, *Atoms in Molecules: A Quantum Theory*, Clarendon Press, 1994.

[18] T. A. Keith, AIMAll (Version 17.11.14), TK Gristmill Software: Overland Park KS, USA, 2017.

[19] J. Contreras-García, E. R. Johnson, S. Keinan, R. Chaudret, J.-P. Piquemal, D. N. Beratan, W. Yang, *J. Chem. Theory Comput.* **2011**, *7*, 625-632.

[20] W. Humphrey, A. Dalke, K. Schulten, *http://www.ks.uiuc.edu/Research/vmd/* **1996**, *14*, 33-38.

[21] M. P. Mitoraj, M. Parafiniuk, M. Srebro, M. Handzlik, A. Buczek, A. Michalak, *J. Mol. Model* **2011**, *17*, 2337.

[22] G. te Velde, F. M. Bickelhaupt, E. J. Baerends, C. F. Guerra, S. J. A. van Gisbergen, J. G. Snijders, T. Ziegler, *J. Comput. Chem.* **2001**, *22*, 931-967.

[23] J. A. Rackers, Z. Wang, C. Lu, M. L. Laury, L. Lagardere, M. J. Schnieders, J.-P. Piquemal, P. Ren, J. W. Ponder, *J. Chem. Theo. Comput.* **2018**, *14*, 5273-5289.

[24] N. L. Allinger, Y. H. Yuh, J.-H. Lii, *J. Am. Chem. Soc.* **1989**, *111*, 8551-8566.

[25] L. J. L. Häller, M. J. Page, S. Erhardt, S. A. Macgregor, M. F. Mahon, M. A. Naser, A. Vélez, M. K. Whittlesey, *J. Am. Chem. Soc.* **2010**, *132*, 18408-18416.

[26] Chemcraft - graphical software for visualization of quantum chemistry computations. https://www.chemcraftprog.com

[27] J. P. Perdew, K. Burke, M. Ernzerhof, *Phys. Rev. Lett.* **1996**, *77*, 3865-3868.

[28] C. Lee, W. Yang, R. G. Parr, *Phys. Rev. B* **1988**, *37*, 785-789.

[29] S. Grimme, *J. Comp. Chem.* **2006**, *27*, 1787-1799.

[30] J. Tao, J. P. Perdew, V. N. Staroverov, G. E. Scuseria, *Phys. Rev. Lett.* **2003**, *91*, 146401.

[31] C. Adamo, V. Barone, *J. Chem. Phys.* **1999**, *110*, 6158-6170.

[32] A. D. Becke, *J. Chem. Phys.* **1993**, *98*, 5648-5652.

[33] Y. Zhao, D. G. Truhlar, *Theor. Chem. Acc.* **2008**, *120*, 215-241.
